# Exposure Contrasts of Women Aged 40–79 Years during the Household Air Pollution Intervention Network Randomized Controlled Trial

**DOI:** 10.1021/acs.est.4c06337

**Published:** 2024-12-30

**Authors:** Wenlu Ye, Devan Campbell, Michael Johnson, Kalpana Balakrishnan, Jennifer L. Peel, Kyle Steenland, Lindsay J. Underhill, Ghislaine Rosa, Miles A. Kirby, Anaité Díaz-Artiga, John McCracken, Lisa M. Thompson, Maggie L. Clark, Lance A. Waller, Howard H. Chang, Jiantong Wang, Ephrem Dusabimana, Florien Ndagijimana, Sankar Sambandam, Krishnendu Mukhopadhyay, Marilu Chiang, Stella M Hartinger, Laura Nicolaou, Kendra Williams, Ricardo Piedrahita, Katherine A. Kearns, Jacob Kremer, Ahana Ghosh, Joshua P. Rosenthal, William Checkley, Thomas Clasen, Luke Naeher, Ajay Pillarisetti

**Affiliations:** † Division of Environmental Health Sciences, School of Public Health, University of California, Berkeley, California 94609, United States; ‡ Department of Environmental Health Sciences, University of Georgia, Athens, Georgia 30602, United States; § Berkeley Air Monitoring Group, Berkeley, California 94701, United States; ∥ Department of Environmental Health Engineering, ICMR Center for Advanced Research on Air Quality, Climate and Health, Sri Ramachandra Institute for Higher Education and Research (Deemed University), Chennai 600001, India; ⊥ Department of Environmental and Radiological Health Sciences, Colorado State University, Fort Collins, Colorado 80523, United States; # Gangarosa Department of Environmental Health, Rollins School of Public Health, Emory University, Atlanta, Georgia 30322, United States; ∇ Global Health Center, Institute for Public Health and Cardiovascular Division, Department of Medicine, Washington University, St. Louis, Missouri 63110, United States; ○ Clean Air (Africa) Global Health Research Group, University of Liverpool, Liverpool L69 3GF, U.K.; ◆ Department of Global Health and Population, Harvard T H Chan School of Public Health, Harvard University, Boston, Massachusetts 02115, United States; ¶ Center for Health Studies, Universidad del Valle de Guatemala, Guatemala City 01015, Guatemala; & Nell Hodgson Woodruff School of Nursing and Gangarosa Department of Environmental Health, Emory University, Atlanta 30322, Georgia, United States; ● Department of Biostatistics and Bioinformatics, Emory University, Atlanta, Georgia 30322, United States; ◊ Eagle Research Centre, Kigali, Rwanda; ▲ Biomedical Research Unit, AB PRISMA, Lima 32, Peru; □ Facultad de Salud Pública y Administración, Universidad Peruana Cayetano Heredia, Lima 15102, Peru; ^ Division of Pulmonary and Critical Care, School of Medicine and Center for Global Non-Communicable Disease Research and Training, Johns Hopkins University, Baltimore, Maryland 21205, United States; ¢ Division of Epidemiology and Population Studies, Fogarty International Center, National Institutes of Health, Bethesda, Maryland 20892, United States

**Keywords:** personal exposure, liquified petroleum gas, particulate matter, black carbon, carbon monoxide, clean fuels, intervention study

## Abstract

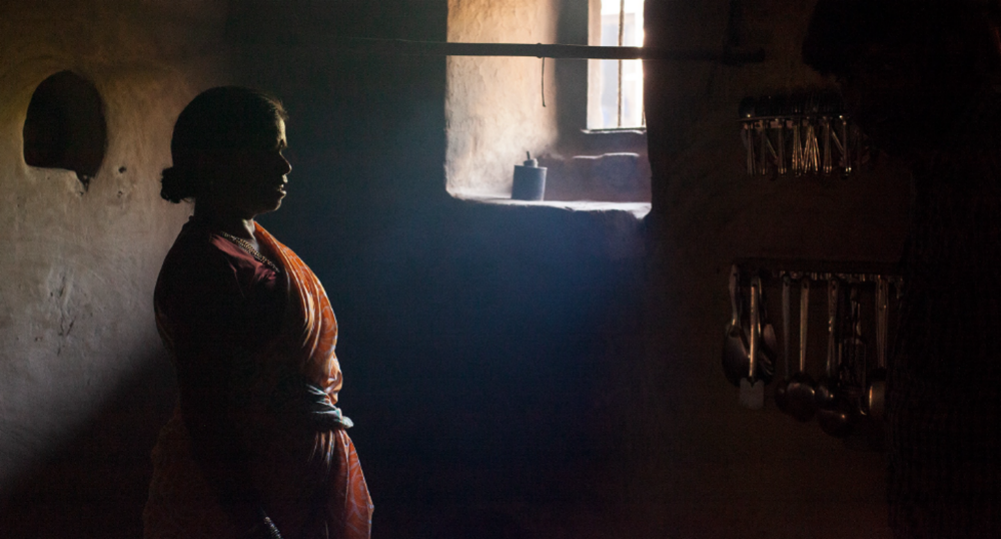

Exposure to household air pollution has been linked to adverse health outcomes among women aged 40–79. Little is known about how shifting from biomass cooking to a cleaner fuel like liquefied petroleum gas (LPG) could impact exposures for this population. We report 24-h exposures to particulate matter (PM_2.5_), black carbon (BC), and carbon monoxide (CO) among women aged 40 to <80 years participating in the Household Air Pollution Intervention Network trial. 209 participants were randomized to the intervention and received an LPG stove and continuous fuel supply; controls used biomass (*n* = 209). Exposures were measured up to six times; we used mixed-effects models to estimate differences between intervention and control groups. Preintervention exposures between groups were comparable; median postintervention exposures were 62% (76.3 vs 29.3 μg/m^3^), 73% (10.4 vs 2.8 μg/m^3^), and 57% (1.4 vs 0.6 ppm) lower for PM_2.5_, BC, and CO among LPG users than for controls. Reductions were similar across countries; 70% of PM_2.5_ exposures after intervention were below the annual WHO interim target I (IT-1) value of 35 μg/m^3^. We provide evidence that implementing an LPG intervention can reduce air pollution exposure over an 18-month period to at or below the annual WHO IT-1 guideline.

## Abstract 2

Little evidence on how shifting from biomass to clean fuel affects the household air pollution exposure of women aged 40−79. This study compares exposure between the intervention and control groups of a liquefied petroleum gas trial with implications for health and energy policy.

## Introduction

Approximately 2.3 billion people, primarily in low- and middle-income countries (LMICs), rely on polluting fuels like wood, dung, kerosene, and crop residues to meet daily cooking energy needs.^1^ Incomplete combustion of these fuels results in exposure to household air pollution (HAP), including particulate matter with an aerodynamic diameter of ≤2.5 μm (PM_2.5_), carbon monoxide (CO), and black carbon (BC), among other hazardous pollutants.^2^ Adverse cardiovascular, respiratory, and neurologic outcomes are associated with HAP exposure among women aged 40–79.^3−8^ HAP is also an important risk factor for hypertension,^9,10^ a major contributor to cardiovascular disease, and the leading risk factor for adverse health among those aged 55 years and older.^11^ In 2021, 3.1 million premature deaths globally were attributed to particulate exposure from HAP, with more than a third of these deaths (∼1.1 million) occurring in women over the age of 50.^11^

To date, many epidemiological studies linking HAP and cardiovascular health outcomes rely upon stove and fuel use categories (i.e., wood as primary fuel) or microenvironmental measures (i.e., kitchen levels) as proxies for exposure, providing effect estimates with less precision.^9,12,13^ For example, a subanalysis conducted by Katz et al. 2020 showed that kitchen concentrations overestimated personal exposure among women that used biomass fuels inside their homes.^14^ Moreover, findings from several studies suggest that women aged >50, compared to their younger counterparts, are more likely to experience increased risks to cardiovascular outcomes, including hypertension, from exposure to HAP.^15−19^

“Improved” biomass or cleaner fuel cookstoves have largely failed to substantially reduce HAP exposures or yield meaningful health benefits in randomized controlled trials.^14,20−24^ Cleaner fuels, like liquefied petroleum gas (LPG), can reduce HAP exposures more than improved biomass stoves; however, concomitant use of biomass, coupled with high levels of ambient background air pollution, may attenuate the potential of HAP-reducing interventions to achieve health-relevant exposure targets.^23^ Due to technological advancements, recent cookstove interventions have measured personal exposures to HAP; however, these measures are typically conducted for women of reproductive age because they are thought to be particularly at risk given the significant amount of time they spend indoors, cooking, or performing childrearing duties.^6,25^ Less is known about exposures and associated health risks among older-aged women, despite the substantial estimated health impact they experience.

To help fill this knowledge gap, we performed 24-h personal exposure assessment on women aged 40 to <80 engaged in cooking activities and residing in the same households as younger pregnant women as part of the multicountry Household Air Pollution Intervention Network (HAPIN) trial of an LPG stove and fuel intervention. Exposures were measured on enrollment (prior to intervention) and at an additional five time points throughout an 18-month period.^26^ Here, we report on the effect of the HAPIN intervention on personal PM_2.5_, BC, and CO exposures among these women.

## Methods

### HAPIN Trial and Study Overview

The HAPIN randomized controlled trial evaluated the health effects of a free LPG stove and continuous fuel intervention versus the use of traditional cookstoves in four countries: Guatemala, India, Peru, and Rwanda. The study design and site have been described in detail elsewhere.^23−26^ Briefly, we selected rural areas in each country with relatively low ambient air pollution, few other air pollution sources, and a high fraction of households that use traditional biomass stoves.^22,27,28^ Sites were chosen to maximize potential exposure reductions from a cleaner stove and fuel intervention. In Guatemala, wood is used indoors in stoves with chimneys and/or in open fires. In India, mud and clay cooking stoves fueled with wood were common. In Peru, households used open fires or chimney stoves fueled by wood and cow dung. In Rwanda, indoor cooking occurred on three-stone wood fires, simple open wood-fueled stoves, and/or charcoal-burning stoves.

### Enrollment

In each country, HAPIN recruited 800 pregnant women 18–35 years old. Participants were enrolled at 9 to <20 weeks gestation, with a viable, ultrasound-confirmed singleton pregnancy. Participants used biomass as a primary fuel for cooking and agreed to participate via informed consent. Among a subset of participating homes, and as the focus of this paper, we enrolled older, nonpregnant adult women (40 to <80 years of age) who lived in the same household as the pregnant HAPIN participant. Older participants were excluded if they used tobacco products or planned to move out of the household in the next year. Enrollment occurred between May 7, 2018 and February 29, 2020.

### Intervention Design

Following a baseline assessment, households were assigned one-to-one randomly to receive a free LPG stove, continuous fuel supply, and behavioral messaging, or to continue using biomass-fueled stoves. In India and Peru, to ensure balance between distinct geographies within each country (2 in India, 6 in Peru), stratified randomization was used. The intervention package was decided upon during formative research.^27,29^ Briefly, all LPG stoves had at least two burners, with additional components to meet cooking needs in each location. LPG stoves and continuous fuel were distributed to intervention households at no cost throughout follow-up. Behavioral messaging included safety training, nudges to exclusively use LPG and to discourage use of traditional stoves. When traditional stove use was detected in intervention homes, behavioral reinforcement visits were made. Participants in intervention homes pledged to use LPG for all cooking throughout the trial. Adherence was high, as reported elsewhere, with limited traditional stove use among households randomized to the intervention.^30,31^

### Air Pollutant Sampling Instrumentation

Exposures to PM_2.5_ were measured with the RTI Enhanced Children’s MicroPEM (ECM, RTI International, Research Triangle Park, USA).^32^ The ECM uses a 2.5 μm size-selective impactor at a flow rate of 300 mL per minute and collects gravimetric samples on 15 mm polytetrafluoroethylene (Teflon) filters (Measurement Technology Laboratories, USA). It also measures real-time PM_2.5_ concentrations via nephelometry and logs temperature, relative humidity, and triaxial accelerometry. The ECM weighs approximately 150 g, is 2.5 cm deep ×6.5 cm tall ×12.5 cm tall, and is nearly silent during use. BC concentrations from PM_2.5_ filter samples were estimated postsampling via transmissometry. We measured concentrations of CO every minute with the Lascar EL-USB-300 (Lascar Electronics); it is the size of a large marker (125 × 26.4 × 26.4 mm), weighs 42g, runs on  AA lithium batteries, measures between 0 and 300 ppm, and has been used previously in HAP assessment.^22,33,34^

### Sampling Strategy

Twenty-four hour personal exposure measurements were collected at six time points at each HAPIN site for each participant. Sampling was conducted during the exposure assessment visits for pregnant participants and their offspring. Measurements were made based on visits to the pregnant women: baseline (“BL”) occurred prior to randomization, from 9 to 20 weeks of pregnancy. Postrandomization follow-up measurements occurred at 24–28 weeks of gestation (Postintervention visit 1, “P1”) and 32–36 weeks of gestation (Postintervention visit 2, “P2”) and at <3 months (Postintervention visit 3, “B1”), ∼6 months (Postintervention visit 4, “B2”), and ∼12 months (Postintervention visit 5, “B4”) after the birth of the pregnant woman’s child. Because recruitment was rolling, measurements were made during most months and all seasons. At each visit, participants wore customized garments^35^ that placed air monitoring instrumentation near the breathing zone.^36,37^ If participants planned to perform activities that could lead to equipment damage (e.g., sleeping, water-intensive work, or bathing), study staff asked them to remove the garment but keep it nearby (within 1–2 m). At the end of the 24-h exposure monitoring period, we conducted a survey that included questions about family members who participated in cooking during that time and other potential sources of exposure.

### Determining PM_2.5_ Mass Concentrations

One μg resolution microbalances (Sartorius Cubis, MSA6.6s-000-DF) located at the University of Georgia (filters from Guatemala, Rwanda, and Peru) and at the Sri Ramachandra Institute for Higher Education and Research (filters from India) were used to assess mass changes pre- and postsampling on filters collected at each exposure visit. Gravimetric data were validated with a three-staged approach: (1) field technicians checked flow rates at the field office before and after sampling with a primary flowmeter to ensure flagging and removal of samples outside of expected ranges; (2) laboratory technicians marked as invalid any filters that were damaged; and (3) data that did not meet quality assurance criteria regarding sampling duration (24 h ± 6 h), flow rate (300 ± 100 mL/min), and inlet pressure (95th percentile, <5 in. H_2_O) were flagged and removed. For samples with invalid gravimetric but valid nephelometric measurements, we applied modeled correction factors obtained from regressions of all valid gravimetric and nephelometric pairs based on the study arm and site to the adjusted 24-h average nephelometer values, resulting in arm and site-specific nephelometric PM_2.5_ concentrations normalized to field-based filter samples. We collected 690 valid field blanks (Guatemala, 217; India, 134; Peru, 259; and Rwanda, 80) for country-specific median blank correction. Limits of detection (LOD) were estimated separately for each site as three times the standard deviation of the blank mass deposition.^38^ We replaced sample depositions below the LOD with LOD/(2^0.5^).^39^

### BC

BC concentrations were estimated from PM_2.5_ filter samples using the SootScan OT-21 Optical Transmissometer (Magee Scientific, USA) at either the University of Georgia (UGA, Athens, GA, USA) or at Sri Ramachandra Institute for Higher Education and Research (SRIHER, Chennai, India). We converted filter absorbance to mass deposition following previously published methods,^40^ using the BC attenuation cross-section value for similar Teflon filters (σ = 13.7 μg/m^2^). Filters collected in Guatemala, Peru, and Rwanda had both a preand postscan. For India, where no prescan occurred, we averaged blank filter postscan values as a substitute. LOD estimation and replacement was as above.

### CO

CO monitors were calibrated using zero air and CO span gas (from 40 to 80 ppm) and checked automatically and at regular intervals via a server-based quality assurance procedure, as well as visually inspected and rated following previous methods.^22^ Calibration occurred every 1–3 months^35^ and applied using the temporally closest calibration coefficient. Data outside sampling duration bounds (24 h ± 6 h) or otherwise flagged due to visually identified response artifacts were removed. Duplicate monitors were deployed to evaluate interunit performance in a subset of households.

### Statistical Analyses

All analyses were performed in R (versions 3.6 and 4.0; R Foundation for Statistical Computing).^41^ We provide summaries of household and participant characteristics by treatment arm and country collected using surveys during the baseline visit. Characteristics for participants with and without missing exposure data are described in the supplement.

For each pollutant, we calculated summaries of valid measurements by study arm (control and intervention), study visit (baseline and postintervention rounds), and by country. We estimated the Spearman correlation coefficient (1) for baseline and postintervention periods and (2) for pollutants by measurement period. These were evaluated overall and stratified by study group (intervention versus control). Differences in pollutant levels were evaluated with nonparametric Wilcoxon Rank Sum, Kruskal–Wallis, and Dunn’s tests.

We calculated the proportion of daily average exposure values that were below or equal to WHO guidelines values. For PM_2.5_, we compared the personal 24-h average measurements to the Annual Interim Target 1 (WHO IT-1) value of 35 μg/m^3^, an attainable target for LMICs.^42^ For CO, we compared personal 24-h averages with the WHO 24-h guideline value of 4 mg/m^3^ (∼3.5 ppm; no annual guideline is provided).^42^

We additionally plotted all measurements by time since intervention to visually depict the stability of exposure reductions. Plots were created first for the entire data set and then by country.

We used statistical methods^22,43,44^ that leverage our repeat measures to evaluate the effect of the HAPIN LPG stove and fuel intervention on exposure. We fit four models to assess distinct comparisons (e.g., before and after, between groups, and comparison of changes by study visit). Model 1 estimated the difference between baseline and postintervention exposures in each arm separately. Model 2 estimated the difference in exposures between arms postintervention. Model 3 estimated the change in exposure for the intervention arm between study phases (pre- or postintervention) relative to the same change in the control arm. Model 4 estimated a similar comparison of changes by study visit. The parameters of interest are the fixed effect for the treatment arm (Model 1), the respective fixed effect for the study phase in each arm (Model 2), the “treatment arm x study phase” interaction term (Model 3), and the “treatment arm x study visit” interaction term (Model 4). We calculated the personal exposure percent reduction attributable to the intervention by exponentiating the parameters of interest, subtracting them from 1, and multiplying them by 100. Exposures were log-transformed given their non-normality.

These models include (1) a random intercept to account for correlation among repeated measurements from the same participants, and (2) an indicator variable for randomization strata when there is more than one. We evaluated nontransformed models to estimate absolute changes in pollutant levels. Finally, we estimated the intraclass correlation coefficient for all measures and by study arm using mixed-effect models with no covariates and a random effect for participant ID.

As supplementary analyses, and in acknowledgment that pregnancy and the arrival of a child may impact exposure of others in the household, including adult women aged 40–79, we summarized pollutant levels and relationships by pregnancy-related study phases: baseline (BL), during pregnancy (P1 and P2), and postbirth (B1, B2, and B4). We additionally compare exposures between pregnant participants and women aged 40–79 to better characterize the difference in exposure between residents in the same household.

The study protocol has been reviewed and approved by institutional review boards (IRBs) and Ethics Committees at Emory University (00089799), Johns Hopkins University (00007403), Sri Ramachandra Institute of Higher Education and Research (IEC-N1/16/JUL/54/49), the Indian Council of Medical Research–Health Ministry Screening Committee (5/8/4–30/(Env)/Indo-US/2016-NCD-I), Universidad del Valle de Guatemala (146–08–2016), Guatemalan Ministry of Health National Ethics Committee (11–2016), Asociación Benefica PRISMA (CE2981.17), the London School of Hygiene and Tropical Medicine (11664–5), the Rwandan National Ethics Committee (No.357/RNEC/2018), and Washington University in St. Louis (201611159). The study has been registered with ClinicalTrials.gov (Identifier NCT02944682).

## Results

### Participant and Household Characteristics

A total of 418 women aged 40 to <80 years were enrolled and completed randomization (209 in the control arm and 209 in the intervention arm). Table 1 summarizes trial-wide and site-specific household and participant characteristics by study arm. We provide a summary of selected characteristics for participants with and without missing exposure data in Supplemental Table S1. The baseline characteristics of the intervention and control groups were similar. The mean age of participants at baseline was 51.8 (SD 7.5) years in the control group and 52.3 (SD 8.2) years in the intervention group. Most participants had no formal education or did not complete primary school in both control (79.9%) and intervention (80.4%) groups. Less than half described themselves as primary cooks in their household at baseline. Households typically reported cooking indoors. Wood and charcoal were primary fuels in Guatemala, India, and Rwanda, while cow dung was used in Peru. Country-specific characteristics are available in the Supporting Information (Table S17).

**Table 1:** Household and Other Adult Women Participants Characteristics at Baseline, by Study Arm^a^

variable	overall	
	control (*n* = 209)	intervention (*n* = 209)
household and kitchen characteristics		
**household size (# people)**		
mean (SD)	6.1 (2.6)	5.9 (2.5)
range	2–18	2–17
**access to electricity**	*n* (%)	*n* (%)
no	26 (12.4%)	20 (9.6%)
yes	182 (87.1%)	187 (89.5%)
missing	1 (0.5%)	2 (1.0%)
**kitchen volume (m^3^)**		
mean (SD)	26.4 (17.6)	25.1 (16.1)
range	4.2 (85.8)	1.8 (69.9)
*n*	180	186
missing (*n*)	29	23
**roof in the kitchen**	*n* (%)	*n* (%)
no	21 (10.0%)	14 (6.7%)
yes	187 (89.5%)	195 (93.3%)
missing	1 (0.5%)	0
**number of stoves**	*n* (%)	*n* (%)
one	74 (35.4%)	74 (35.4%)
two	113 (54.1%)	112 (53.6%)
three or more	21 (10.0%)	23 (11.0%)
missing	1 (0.5%)	0
**primary stove has a chimney**	*n* (%)	*n* (%)
no	162 (77.5%)	167 (79.9%)
yes	46 (22.0%)	42 (20.1%)
missing	1 (0.5%)	0
**primary cook**	*n* (%)	*n* (%)
pregnant women	110 (52.9%)	115 (55.0%)
other adult women	92 (44.2%)	91 (43.5%)
other/missing	6 (2.9%)	3 (1.4%)
**primary fuel type**	*n* (%)	*n* (%)
cow dung	63 (30.1%)	54 (25.8%)
wood	145 (69.4%)	148 (70.8%)
charcoal	0	3 (1.4%)
other	0	4 (1.9%)
missing	1 (0.5%)	0
**primary stove location**	*n* (%)	*n* (%)
in participant’s bedroom	8 (3.8%)	13 (6.2%)
room immediately adjacent to the participant’s bedroom	44 (21.1%)	35 (16.7%)
separated from the participant’s bedroom but inside the house	45 (21.5%)	58 (27.8%)
outside the house (outdoors)	25 (12.0%)	14 (6.7%)
in a separate building detached from the bedroom-main home	86 (41.1%)	89 (42.6%)
missing	1 (0.5%)	0
**primary light source**	*n* (%)	*n* (%)
torch (battery)	4 (1.9%)	6 (2.9%)
kerosene lamp	4 (1.9%)	4 (1.9%)
solar light	13 (6.2%)	10 (4.8%)
electricity	179 (85.6%)	178 (85.2%)
other	8 (3.8%)	11 (5.3%)
missing	1 (0.5%)	0
**presence of a smoker in home**	*n* (%)	*n* (%)
no	179 (85.6%)	182 (87.1)
yes	29 (13.9%)	27 (12.9%)
missing	1 (0.5%)	0
participant characteristics		
**age (year)**		
mean (SD)	51.8 (7.5)	52.3 (8.2)
range	40.1–73.8	40.2–74.3
**occupation**	*n* (%)	*n* (%)
agriculture	71 (34.0%)	65 (31.1%)
commercial	5 (2.4%)	10 (4.8%)
household	122 (58.4%)	120 (57.4%)
other	8 (3.8%)	7 (3.3%)
unemployed	3 (1.4%)	7 (3.3%)
**education**	*n* (%)	*n* (%)
no formal education or primary school incomplete	167 (79.9%)	168 (80.4%)
primary school or secondary school incomplete	31 (14.8%)	34 (16.3%)
secondary school or vocational or some college/university	7 (3.3%)	3 (1.4%)
missing	4 (1.9%)	4 (1.9%)

a Summary based on 418 adult women aged 40–79 enrolled in the HAPIN trial.

### Exposure Data Completeness, Compliance, and Quality Assessment and Control

All participants (*n* = 418) had at least one valid PM_2.5_ measurement. Among them, 88% had three or more valid PM_2.5_ exposure measurements during the 18-month study period. Approximately 7% of our total samples had invalid gravimetric samples; these data were replaced with adjusted nephelometer values using modeled correction factors, as detailed elsewhere.^35,44^

For both BC and CO, 80% of the participants had three or more valid measurements. All participants with valid baseline measurements had at least one valid postrandomization measurement for PM_2.5_, BC, and CO. The numbers and percentages of exposure samples successfully collected by visit and country are presented in Table S2. The final data set, as reported here, includes 1731 PM_2.5_, 1555 BC, and 1580 CO samples. Sample validity details are in Table S3.

### Exposure Summary

**Figure 1:**
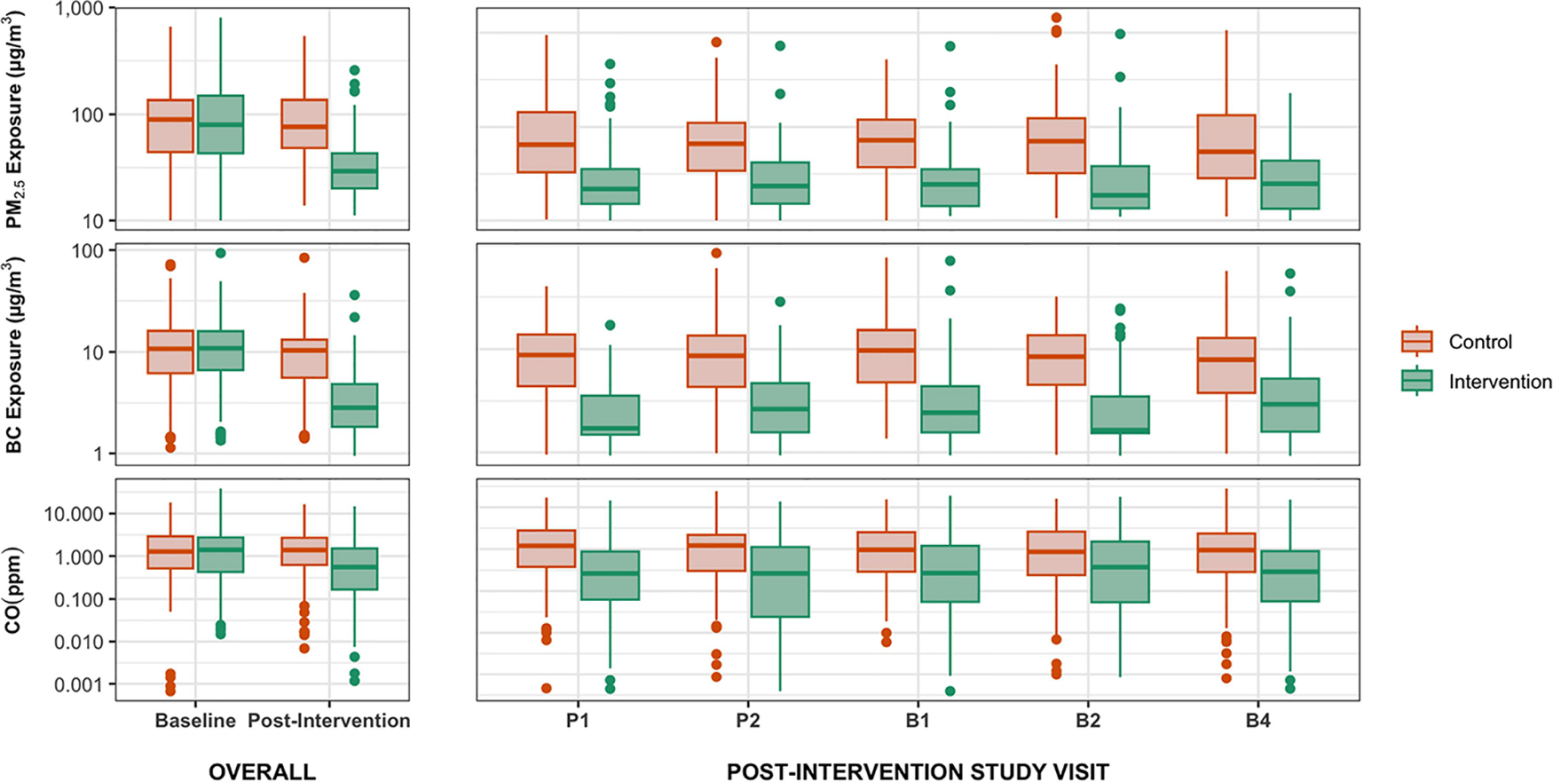
Personal exposures to PM_2.5_, BC, and CO by study arm. The “Overall” panel compares baseline values with average exposures postintervention. The “Post-intervention Study Visit” panel shows values by study visit (all postintervention). The solid line in each box is the median. The hinges correspond to the 25th and 75th percentiles. The whiskers extend 1.5× the interquartile range above and below the upper and lower hinges. Data beyond the whiskers are outliers. Baseline (“BL”, 9 and 20 weeks gestation), Postintervention visit 1 (“P1”, 24–28 weeks of gestation), Postintervention visit 2 (“P2”, 32–36 weeks of gestation), Postintervention visit 3, “B1” (child <3 months of age), “B2” (<6 m), “B4” (<12 m). *Y*-axes are on the log scale.

24-h average personal exposure to PM_2.5_, BC, and CO by study arm at baseline and postintervention are summarized in Table 2 and displayed graphically in Figure 1 (country-specific plots are in Figures S1–S3 for PM_2.5_, BC, and CO, respectively). Trial-wide at baseline, there was no statistically significant difference in PM_2.5_ exposure (Wilcoxon rank sum, *p* = 0.73) between the control group (median: 89.4 μg/m^3^; IQR: 44.2–135.6) and intervention group (median: 79.8 μg/m^3^; IQR: 43.0–148.5). Baseline BC (Wilcoxon rank sum, *p* = 0.95) and CO (Wilcoxon rank sum, *p* = 0.71) exposures were also similar between arms. Median (IQR) exposures to BC and CO were 10.7 μg/m^3^ (6.2–16.1) and 1.3 ppm (0.5–2.9) in the control group and 10.9 μg/m^3^ (6.7–16.0) and 1.4 ppm (0.4–2.7) in the intervention group.

**Table 2:** Summary of Valid Personal Exposure to PM_2.5_, BC, and CO of Other Adult Women Participants by Study Group

	PM_2.5_ exposure (μg/m^3^)		BC exposure (μg/m^3^)		CO exposure (ppm)	
	control	intervention	control	intervention	control	intervention
baseline						
*N*	192	190	166	167	169	174
average (SD)	112.7 (100.4)	124.4 (137.5)	12.6 (10.3)	13.1 (11.3)	2.2 (2.7)	2.4 (4.1)
range	10–660.8	10–803.4	1.1–72.3	1.3–93.3	0–18.1	0–38.7
median (IQR)	89.4 (44.2–135.6)	79.8 (43–148.5)	10.7 (6.2–16.1)	10.9 (6.7–16)	1.3 (0.5–2.9)	1.4 (0.4–2.7)
postintervention^a^						
*N*	194	200	191	200	186	198
# measures (SD)	3.4 (1.2)	3.4 (1.1)	3.1 (1.2)	3.2 (1.2)	3.2 (1.2)	3.2 (1.2)
average (SD)	111.4 (100.6)	38.1 (31.5)	10.4 (7.8)	4.0 (3.7)	2.1 (2.4)	1.3 (2.1)
range	13.9–540.1	11.2–257.6	1.4–83.9	0.9–36.1	0–16.4	0–14.8
median (IQR)	76.3 (48.3–136.3)	29.3 (20.2–43.0)	10.4 (5.6–13.2)	2.8 (1.8–4.8)	1.4 (0.6–2.7)	0.6 (0.2–1.5)

a Summary of the average of repeat measures across all postintervention visits.

Median postintervention exposure to PM_2.5_ in the intervention arm (29.3 μg/m^3^) was 62% lower compared to that in the control arm (76.3 μg/m^3^). BC exposures in the intervention group were 73% lower (2.8 vs 10.4 μg/m^3^). CO exposures were lower in the intervention group by 57% (0.6 vs 1.4 ppm). Decreases in exposure were consistent between rounds (Figure 1, Supplemental Tables S4–S7). Findings were also consistent across countries, though the magnitude of reductions varied (Tables S5–S7 and Figures S1–S3). Exposure data by select housing and participant characteristics are included in Supporting Information (Tables S18–S20).

### Correlations between Measurement Rounds and between Pollutants

We observed moderate to low correlation (Spearman’s ρ) between all three pollutants during all measurement rounds (Tables S8 and S9). We observed moderate correlation (trial-wide Spearman’s ρ = 0.53) between PM_2.5_ and CO among the traditional stove households (including the intervention group at baseline, prior to intervention, and all control group measurements). The PM-CO exposure correlation is much weaker in LPG-using households (overall Spearman’s ρ = 0.11), with varying correlations by country (Figure S4). The correlation between BC and CO among traditional stove households was also moderate (trial-wide Spearman’s ρ = 0.49) and much weaker among LPG households (trial-wide Spearman’s ρ = 0.07) (Figure S5). We found a stronger correlation between PM_2.5_ and BC, with a trial-wide Spearman’s ρ of 0.76 in the traditional stove households and 0.60 in the LPG stove households; some heterogeneity between countries was noted (Figure S6).

### Exposures Meeting the Annual WHO Interim Target Guidelines

At baseline, 19.3% (*n* = 37) and 21.1% (*n* = 40) of PM_2.5_ measurements were less than or equal to the annual 35 μg/m^3^ WHO IT-1 for PM_2.5_ in the control and intervention arms, respectively. During the postintervention period, 26.9% (*n* = 177) of control and 70% (*n* = 478) of the intervention exposures were below WHO-IT1. 53% (*n* = 364) of intervention exposures were at or below WHO IT-2 of 25 μg/m^3^.

For CO, 81 and 85% of the 24-h exposures in the control and intervention arms, respectively, were below the WHO annual guideline value (3.5 ppm) at baseline. Postintervention, 84% of control CO exposures were below the guideline value, whereas 91% of the intervention exposures were less than the guideline value.

### Exposures over Time

**Figure 2:**
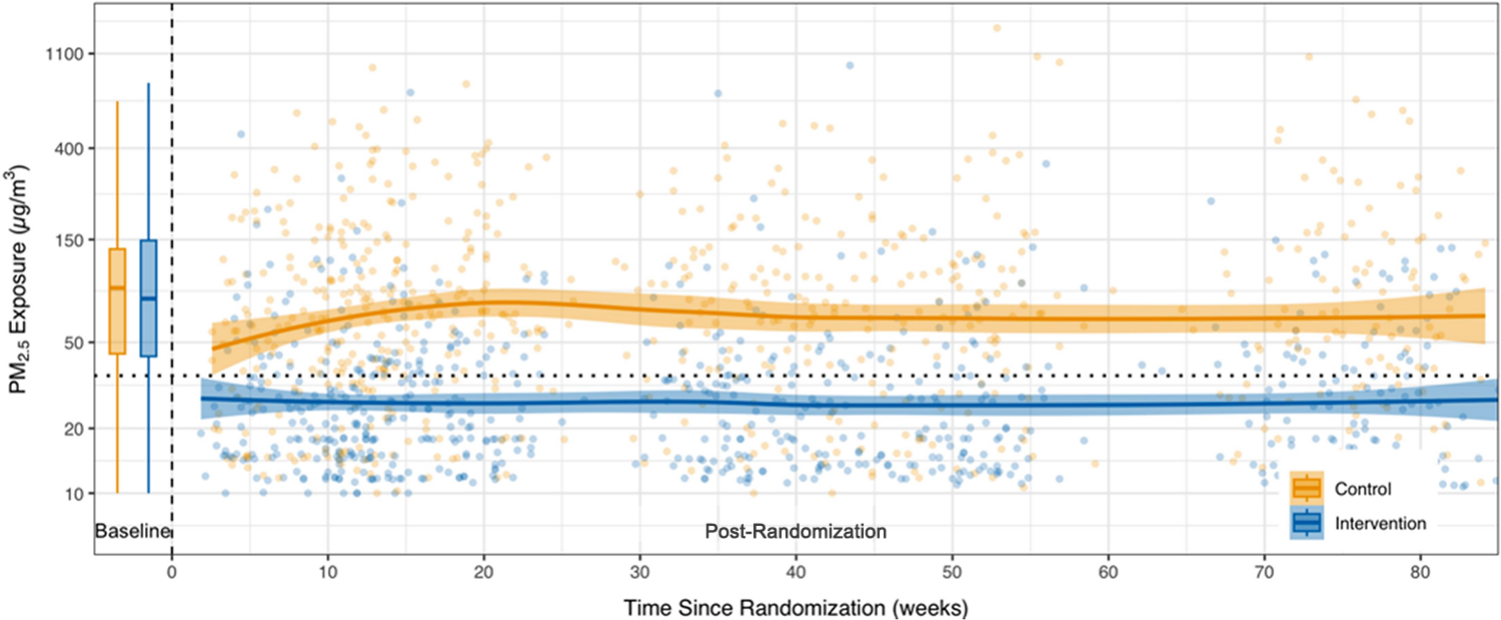
Personal PM_2.5_ exposure trends pre- and postintervention. Time since randomization is on the *x*-axis in weeks; time before 0 indicates the baseline period. Baseline exposures are presented as box plots. Lower and upper hinges correspond to the 25th and 75th percentiles. The whiskers extend 1.5 × IQR above and below the hinges. Data beyond the whiskers are outliers. Solid lines are a locally weighted smoothing (LOESS) model. Shaded areas are standard errors. Orange (light) points are data points from control homes; blue (dark) points are from intervention homes. Note: IQR, interquartile range.

We plotted exposures to PM_2.5_ by time since randomization overall (Figure 2) and in each country (Figure S7). The plot highlights a similar distribution of PM_2.5_ exposures at baseline (*p* = 0.73) but a distinct separation of exposures between the control and intervention groups postintervention. Site-specific personal PM_2.5_ exposure trends followed a similar pattern, although the magnitude of exposures and exposure contrasts vary between sites. Among the control households, we observed a small but statistically significant reduction in PM_2.5_ between baseline and the postintervention period: 89.4 and 76.3 μg/m^3^, respectively. We noted a less pronounced reduction for black carbon (10.7–10.4 μg/m^3^). We also plotted PM_2.5_ data by calendar date (Figures S13–S15); exposures are also stable over calendar time.

### Modeling Results

**Figure 3:**
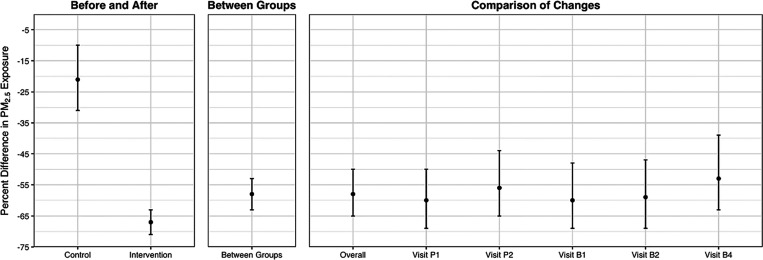
Estimated effects of the HAPIN LPG stove and fuel intervention on PM_2.5_ exposure. All models used log-transformed PM_2.5_ as the dependent variable. Whiskers are the 95% confidence interval.

We assessed the effect of the HAPIN LPG cookstove and fuel intervention on personal exposure using different modeling strategies: “between groups,” “before and after,” and “comparison of changes.” All models showed significant reductions in all three pollutants (Table 3, Supplemental Table S10). Visualization of the results across models for PM_2.5_ is shown in Figure 3 (results for BC and CO are shown in Supplemental Figures S8 and S9). The three modeling approaches yield similar estimated percent reduction in PM_2.5_ exposure due to the intervention: 59% (95% CI: 55%, 63%) for the “between groups” approach; 67% (95% CI: 63%, 71%) for the “before and after” approach; and 59% (95% CI: 51%, 65%) for the “comparison of changes” approach (Table 3). The reductions were similar for BC but more pronounced for CO (Table 3).

**Table 3:** Percent Decreases in PM_2.5_, BC, and CO Exposure Associated with LPG Intervention^a^

model type	details	% decrease in PM2.5 exposure	% decrease in BC exposure	% decrease in CO exposure
		estimate (CI)	estimate (CI)	estimate (CI)
between groups	–	59 (55, 63)	60 (56, 65)	73 (65, 80)
before and after	control	20 (9, 29)	25 (15, 33)	21 (−5, 40)
	intervention	67 (63, 71)	70 (67, 74)	79 (70, 85)
comparison of changes	overall	59 (51, 65)	61 (54, 67)	74 (60, 83)

a –, no data; BC, black carbon; CI, confidence interval; CO, carbon monoxide; LPG, liquefied petroleum gas.

We also modeled untransformed exposures to show the absolute mean reductions. For PM_2.5_, the absolute reductions were 73 (95% CI: 58, 87) μg/m^3^ for “between groups,” 87 (95% CI: 73, 101) μg/m^3^ for “before and after”, and 85 (95% CI: 61, 108) μg/m^3^ for the “comparison of changes”. The “before and after” approach also indicated a 20% (95% CI: 9%, 29%) reduction in exposure between baseline and postintervention periods for the control group. The visit-specific “comparison of changes” models presented consistent percent reductions in personal PM_2.5_/BC/CO exposures across visits, indicating the effectiveness of the LPG stove and fuel intervention in reducing exposures over time. Country-specific reductions generally reflect the trial-wide pattern, although the magnitude varied.

### Variability within and between Participants

ICCs were assessed for all participants (excluding baseline) and then separately for interventions (excluding baseline values, when biomass stoves were used) and for controls (all observations). We observed a relatively low ICC for all pollutants, indicating high with-in person variability: PM_2.5_ measurements (overall: 0.43; control: 0.36; intervention: 0.12), BC (overall 0.58; control: 0.38; intervention: 0.33) and CO (overall: 0.30; control: 0.23; intervention: 0.20).

### Comparison with Exposures of Pregnant Adult Women

24-h exposures for nonpregnant adult women and pregnant women living in the same household are summarized in Supplemental Table S11 and visualized in Figures S10–S12. IRC and country-specific tables are in Supplemental Tables S12–S14. In control households trial-wide, median PM_2.5_ exposures were significantly higher for nonpregnant women compared to pregnant women at both baseline (89.3 vs 72.6 μg/m^3^; Dunn’s *p* = 0.03) and postbirth (75.6 vs 54.9 μg/m^3^; Dunn’s *p* = 0.02) periods. In intervention households, median PM_2.5_ exposures for nonpregnant women were higher than those for pregnant women during pregnancy (26.6 vs 20.6 μg/m^3^; Dunn’s *p* = 0.002) and postbirth (26.1 vs 20.3 μg/m^3^; Dunn’s *p* = 0.008). Other pollutant exposures were generally not statistically different between participant types in either treatment arm, except during the postbirth period, where median BC (8.1 vs 5.9 μg/m^3^; Dunn’s *p* = 0.05) and CO (0.3 vs 0.2 μg/m^3^; Dunn’s *p* = 0.003) exposures for nonpregnant women were significantly higher than their counterparts. Correlations of exposure between nonpregnant and pregnant participants living in the same households are presented in Table S15.

## Discussion

We contribute to the literature on exposures to women between the ages of 40 and <80 in four diverse LMIC settings. Our main findings show that the 18-month HAPIN intervention of an LPG cookstove and continued fuel supply led to a substantial and significant reduction in personal exposures to PM_2.5_, BC, and CO for women aged 40 through 79 living in the same household as the pregnant HAPIN participant. In the intervention group, the overall median postintervention PM_2.5_ exposure was 29.3 μg/m^3^, representing a 62% reduction from baseline (76.3 μg/m^3^). In these women, 70% of the postintervention PM_2.5_ exposures fell below the annual WHO IT-1 of 35 μg/m^3^, and 53% were at or below WHO IT-2 of 25 μg/m^3^. The overall median BC and CO exposures in the intervention group were 73 and 57% lower, respectively, in comparison with baseline measures. Over the 18-month intervention period, average PM_2.5_ exposures varied by ∼6 μg/m^3^ or less (Table 2; Figure 2), indicating a stable exposure reduction throughout the study consistent with sustained use of the LPG stove in intervention households. Our findings demonstrate the largest noted reduction in personal exposures to three major household air pollutants among several cleaner household energy intervention studies.

HAPIN has now demonstrated substantial exposure reductions for pregnant women, their newborn children (under 1 year of age), and for women aged 40–79, all living in the same house. Differences in exposure distributions between subpopulations are likely due to behavioral changes associated with pregnancy, such as dietary requirements, physical activity, time spent at home, cooking activity, occupation, and child-rearing activities.^45,46^ Although we observed moderate to strong correlations between pollutant exposures of nonpregnant and pregnant women in our study, these correlations varied considerably across countries: consistently weaker correlations were found in Peru compared to Guatemala and India, suggesting that differences in time-activity patterns between participant groups may influence exposures. We also found relatively low ICCs, both overall and by study group, indicating high within-person variability. This may be driven in part by changing roles as the other HAPIN participant in the household progressed through pregnancy. Alternatively, it may be that the participants described in this manuscript were more mobile than their pregnant counterparts, resulting in more variable pollutant exposures over time. Future work should interrogate more thoroughly time-activity patterns.

In general, we found that women aged 40–79 had higher exposures than their pregnant counterparts. These differences were statistically significant for all pollutants among intervention households during pregnancy. Our findings align with previous studies on cookstove interventions, which demonstrated statistically significant reductions in CO concentrations for pregnant women in Guatemala^18^ and India^47^ but not for their nonpregnant counterparts. We did not track detailed time activity patterns of HAPIN participants, but hypothesize that these differences in exposure may be due to differences in mobility and daily activities between pregnant and nonpregnant participants. We note that pregnant women achieved lower overall exposures postintervention (Supporting Information Tables S11 and S12). The results of our study, particularly in intervention households, were mainly driven by participants from Peru. This is the only country where significant differences in exposure were observed for all pollutants during each postintervention period. The low correlations between pregnant and nonpregnant women in Peru suggest the presence of distinct time-activity patterns that contribute to exposure differences. Further analyses are necessary to better characterize these differential behavioral patterns.

### Exposure Comparisons with Previous Studies

Our previous work characterized exposure reductions associated with LPG use among pregnant women enrolled in HAPIN.^44^ That study reported statistically significant exposure reductions, after adjusting for those seen in the control group, of 62, 62, and 82% for PM_2.5_, BC, and CO, respectively. These findings are similar to reductions (62% for PM_2.5_, 73% for BC, 57% for CO) in the current study. Median postintervention exposures for pregnant women in HAPIN intervention households (15–34 μg/m^3^ for PM_2.5_; 2.7–2.8 μg/m^3^ for BC; and 0.2 ppm for CO) were within the ranges we report for corresponding nonpregnant women. These findings suggest that the HAPIN intervention package improved air quality for individuals who typically may not have been the household’s primary cook.

Other recent HAP studies (Supplemental Table S16) provide notable yet imperfect comparisons. In Guatemala, Grajeda et al.^48^ reported median exposures for pregnant women who owned LPG stoves (55 μg/m^3^) and those who did not (78 μg/m^3^) (in comparison with 23–29 and 57–107 μg/m^3^ in the intervention and control arms, respectively, for the HAPIN site in Guatemala), and estimated that LPG ownership was associated with a 38% reduction in PM_2.5_. Weinstein et al.^49^ found that the median PM_2.5_ exposure level among Guatemalan women cooking exclusively with biomass (102 μg/m^3^) decreased when they were provided with LPG stoves (45 μg/m^3^). Additionally, median PM_2.5_ and BC exposures among Guatemalan women in control homes in the current study were comparable to those among women in rural Honduras using traditional biomass cookstoves.^50,51^ Thornburg et al.^52^ reported a 31% reduction in personal PM_2.5_ (from 103.5 to 71.5 μg/m^3^) from an LPG intervention among pregnant women in Bangladesh. Raqib et al.^53^ observed a 43.5% (average decreased from 158.9 to 85.6 μg/m^3^) and 12.9% (average decreased from 7.36 to 6.27 μg/m^3^) reduction in PM_2.5_ and BC, respectively (in comparison to the before and after percent reductions for PM_2.5_ (63%) and BC (70%) observed among intervention participants in the HAPIN India site). In Rwanda, a trial of rocket-style cookstoves and water filters^54^ reported median exposures of 146 and 158 μg/m^3^ in the control and intervention arms, respectively, for the primary cook (in comparison with 69–106 μg/m^3^ in the control arm and 20–57 μg/m^3^ postintervention for the HAPIN site in Rwanda). The large, eight country PURE-AIR study offers another frame of comparison^55^; female participants on whom PM_2.5_ exposure was measured were, on average, 59 years old (SD 10). Those using gas had estimated PM_2.5_ geometric mean exposures of 48 μg/m^3^ (95% CI 43–54), while wood users had exposures of 78 μg/m^3^ (95% CI 69–89). Exposures using wood are similar to those for control households and intervention households at baseline for the current study; gas exposures in the current study were lower, likely because mixed use of traditional and clean fuels was minimized.

Special considerations are necessary when comparing exposure estimates from HAPIN to those from other relevant HAP studies. As an efficacy trial, HAPIN’s study design aimed to understand the maximum achievable exposure reduction by implementing strategies to support exclusive LPG use and ensure stove maintenance.^26^ The high adherence to the HAPIN intervention may explain the lower exposures among LPG users in HAPIN relative to those observed in the numerous studies shown in Supplemental Table S16. Moreover, studies like Alexander et al.^20^ have cited both mixed fuel use and ambient air pollution as potential reasons for consistently elevated personal exposures among LPG users that exceed health-relevant targets. Additionally, while Chillrud et al.^22^ did not measure ambient pollution the authors found a positive association between air pollution exposure and population density, highlighting a “neighborhood effect” that could attenuate exposures between groups.

### Study Strengths

The current study demonstrates several notable strengths. First, we rigorously examined the impact of a cookstove and fuel intervention on personal exposures for women aged 40–79. We used state-of-the-science methods, including a combined nephelometer and gravimetric sampler, and rigorous QA–QC procedures. Second, extensive pretrial testing allowed us to develop targeted strategies aimed at promoting exclusive LPG use. This, in turn, resulted in high adherence (>96%), measured through a combination of sensors, observations, and questionnaires, to the cookstove intervention implemented throughout HAPIN and allowed us to observe large exposure reductions due to LPG use.^30^ Additionally, we established standard practices for data collection, cleaning, and analysis, ensuring the internal and external credibility of our exposure estimates.^35,56^ Third, we conducted comprehensive exposure assessment, collecting up to six repeated 24-h measurements of multiple pollutant exposures per participant. This longitudinal design allowed us to capture exposure dynamics over time and to characterize the impact of the intervention overall and by study visit; we found consistent reductions in exposure among households with the LPG stove and fuel intervention. Finally, we note that our study enables comparison with pregnant women and their young children living in the same household, providing valuable information on exposure to multiple householders. Data on multiple individuals in the same home across a range of ages remains uncommon in household air pollution exposure assessments.

### Study Limitations

Our study also has some limitations. First, as an efficacy trial, HAPIN provided free LPG cookstoves and a continuous fuel supply over the entire study period. Combined with behavioral reinforcement activities as needed, the trial achieved high fidelity and exclusive use of the intervention.^31,57^ A similar exposure contrast between the LPG and biomass cookstove might be hard to observe in contexts without such intensive support. Moreover, we deliberately selected study sites without major air pollution point sources.^26−28^ This could limit the applicability of our findings to areas with garbage burning, road traffic, and industrial pollution, among other potential sources of exposure.

Second, although the HAPIN trial collected up to six 24-h measurements over the 18-month study period (roughly three months apart), more measurements may be needed to fully characterize exposure over time, resulting in some risk of exposure misclassification. An intensive field sampling campaign in Guatemala indicated that >48 h sampling duration reduces measurement variation and that repeated sampling per week or month led to a higher probability of being closer to the “true” long-term mean.^58^ Still, our findings showed that high adherence to the intervention resulted in stable exposure reductions (Figures 2 and 3), suggesting that our measurements provided a reasonable estimate of longer-term average exposures. Another source of exposure measurement error may come from wearing compliance of exposure instruments. We could not rule out the possibility that participants changed their behavior (i.e., stayed home, altered time-activity patterns) while wearing the exposure instrument during the sampling period, leading to a departure from their “true” exposure.

Third, although exposure levels among controls remained high, we observed a ∼20% reduction in the control group postintervention. This might be due to the nature of the intervention and study design: participants and field workers were not blinded to study arm, and the frequent interactions between participants and the field team for exposure and health evaluation may have improved awareness of harmful HAP exposures and led to behavior changes in the control group. If this was the case, the contrast between LPG and biomass exposures could have been more prominent and the observed percentage reduction may be an underestimate.

Additionally, some sample loss was inevitable, especially given the large number of participants followed over a long study period and during the COVID-19 pandemic. The trial suspended data collection due to the pandemic in March 2020 and resumed household visits during the fifth year of the trial.^59^ The lockdown impacted some postintervention visits (i.e., B1, B2, and B4). Among 418 enrolled, on average, 75% had successful exposure visits prepandemic compared to 52% during the pandemic (Table S2). Finally, we note that there may have been changes in family responsibilities during the other HAPIN participant’s pregnancy and the subsequent first year of life. This may have shifted cooking responsibilities to unmonitored household members who we were unable to monitor during this study. We did not collect detailed time-activity data for our participants, which may have enabled better exposure apportionment to specific activities. Nonetheless, we note that we saw a consistent and clear decrease in exposure in households who received the LPG fuel and stove intervention.

This analysis suggests that an 18-month LPG cookstove/fuel intervention can substantially and consistently reduce personal HAP exposure among nonpregnant women aged 40 to <80 living in households that rely on solid fuels. The trial collected up to six personal PM_2.5_, BC, and CO exposure measurements per participant and is one of the largest and most comprehensive personal air pollution exposure monitoring efforts in the context of cleaner cooking interventions and HAP to date. The exposure contrast between women using biomass and LPG cookstoves/fuel is among the largest of all other household energy intervention studies. As an efficacy trial with high fidelity and adherence to the intervention, HAPIN showed high exposure reductions from using LPG for cooking in four LMICs characterized by diverse socioeconomic, cultural, behavioral, and environmental factors. Our findings provide evidence that implementing a cleaner household energy intervention can effectively reduce personal air pollution exposure and achieve levels below the annual WHO IT-1 target of 35 μg/m^3^ for multiple adult women in the same household.

## Supporting Information Available

Selected characteristics of households and participants with and without PM_2.5_ exposure measurements by visit and study arm; overall exposure data completeness of other adult women participants in HAPIN Trial; gravimetric sample validity 6; summary of valid personal exposure to PM_2.5_, BC, and CO of adult women aged 40−79 by country and study group; summary of personal exposure to PM_2.5_ of other adult women participants by IRC and study group; summary of personal exposure to BC of other adult women participants by IRC and study group; summary of personal exposure to CO of other adult women participants by IRC and study group; correlations (Spearman’s ρ) between pollutants by stove type and IRC; correlations (Spearman’s ρ) of personal exposure to PM_2.5_, BC, and CO between measurement rounds; percent decreases in PM_2.5_, BC, and CO exposure associated with LPG intervention (trial-wide); comparison of personal exposures to PM_2.5_ between pregnant and non-pregnant adult women in the same households by arm and study period; comparison of personal exposures to BC between pregnant and non-pregnant adult women in the same households by arm and study period; comparison of personal exposures to CO between pregnant and non-pregnant adult women in the same households by arm and study period; correlation (Spearman’s ρ) of personal measures of exposure between non-pregnant and pregnant adult women by pollutant, stove-type, IRC, and study period; personal exposures from comparable household air pollution studies; household and participants characteristics at baseline, by IRC and study arm; PM_2.5_ exposures by specific household/participants characteristics for each country, study arm, and study period; CO exposures by specific household/participants characteristics for each country, study arm, and study period; BC exposures by specific household / participants characteristics for each country, study arm, and study period; boxplot of personal exposure to PM_2.5_ among non-pregnant adult women participants by IRC, study group, and visit; boxplot of personal exposure to BC among non-pregnant adult women participants by IRC, study group, and visit; boxplot of personal exposure to CO among non-pregnant adult women participants by IRC, study group, and visit; correlation between log10-transformed PM_2.5_ and CO exposure by stove type and IRC; correlation between log10-transformed BC and CO exposure by stove type and IRC; correlation between log10-transformed PM_2.5_ and BC exposure by stove type and IRC; trends in personal PM_2.5_ exposure among the HAPIN non-pregnant adult women participants in each IRC; estimated effects of the HAPIN LPG stove and fuel intervention on BC exposure; estimated effects of the HAPIN LPG stove and fuel intervention on CO exposure; trial-wide and IRC-specific comparison of personal exposures to PM_2.5_ between pregnant and non-pregnant adult women in the same households by arm and study period; trial-wide and IRC-specific comparison of personal exposures to BC between pregnant and non-pregnant adult women in the same households by arm and study period; trial-wide and IRC-specific comparison of personal exposures to CO between pregnant and non-pregnant adult women in the same households by arm and study period; and baseline Measurements by Calendar Date (PDF)
