# Supplementary material for: Exposure Contrasts of Women Aged 40–79 Years during the Household Air Pollution Intervention Network Randomized Controlled Trial
Source: Environ Sci Technol. 2024 Dec 30;59(1):69–81. doi: 10.1021/acs.est.4c06337 (PMC11740992; doi:10.1021/acs.est.4c06337)
Supplement: Supplementary file 1 — es4c06337_si_001.pdf [file es4c06337_si_001.pdf]

Supporting information for

Exposure Contrasts of Women aged 40-79 years during the Household Air Pollution Intervention Network Randomized Controlled Trial

Wenlu Ye [a§], Devan Campbell [b§], Michael Johnson [c], Kalpana Balakrishnan [d], Jennifer L. Peel [e], Kyle Steenland [f], Lindsay J. Underhill [g], Ghislaine Rosa [h], Miles A. Kirby [i], Anaité Díaz-Artiga [j], John McCracken [b], Lisa M. Thompson [k], Maggie L. Clark [e], Lance A. Waller [l], Howard H. Chang [l], Jiantong Wang [l], Ephrem Dusabimana [m], Florien Ndagijimana [m], Sankar Sambandam [d], Krishnendu Mukhopadhyay [d], Marilu Chiang [n], Stella M Hartinger [o], Laura Nicolaou [p], Kendra Williams [p], Ricardo Piedrahita [c], Katherine A.
Kearns [b], Jacob Kremer [b], Ahana Ghosh [c], Joshua P. Rosenthal [q], William Checkley [p], Thomas Clasen [f], Luke Naeher [b*], Ajay Pillarisetti [a*]

[a] Division of Environmental Health Sciences, School of Public Health, University of California, Berkeley, CA 94609, USA
[b] Department of Environmental Health Sciences, University of Georgia, Athens, GA 30602, USA
[c] Berkeley Air Monitoring Group, Berkeley, CA 94701, USA
[d] Department of Environmental Health Engineering, ICMR Center for Advanced Research on Air Quality, Climate and Health, Sri Ramachandra Institute for Higher Education and Research (Deemed University), Chennai 600001, India
[e] Department of Environmental and Radiological Health Sciences, Colorado State University, Fort Collins, CO 80523, USA
[f] Gangarosa Department of Environmental Health, Rollins School of Public Health, Emory University, Atlanta, GA 30322, USA
[g] Global Health Center, Institute for Public Health and Cardiovascular Division, Department of Medicine, Washington University, St. Louis, MO 63110, USA
[h] Clean Air (Africa) Global Health Research Group, University of Liverpool, Liverpool L69 3GF, UK
[i] Department of Global Health and Population, Harvard T H Chan School of Public Health, Harvard University, Boston, MA 02115, USA
[j] Center for Health Studies, Universidad del Valle de Guatemala, Guatemala City 01015, Guatemala
[k] Nell Hodgson Woodruff School of Nursing and Gangarosa Department of Environmental Health, Emory University, Atlanta 30322, GA, USA
[l] Department of Biostatistics and Bioinformatics, Emory University, Atlanta 30322, GA, USA
[m] Eagle Research Centre, Kigali 00000, Rwanda
[n] Biomedical Research Unit, AB PRISMA, Lima 32, Peru
[o] Facultad de Salud Pública y Administración, Universidad Peruana Cayetano Heredia, Lima 15102, Peru
[p] Division of Pulmonary and Critical Care, School of Medicine and Center for Global Non-Communicable Disease Research and Training, Johns Hopkins University, Baltimore, MD 21205, USA
[q] Division of Epidemiology and Population Studies, Fogarty International Center, National Institutes of Health, Bethesda, MD 20892, USA

[*]Email: ajayp@berkeley.edu
[*]Email: lnaeher@uga.edu
[§] *W.Y and D.C. contributed equally to this work*

Summary: 43 Pages, 15 figures, 20 tables

TABLES 4

Table S1. Selected characteristics of households and participants with and without $PM_{2.5}$ exposure measurements by visit and study arm 4

Table S2. Overall exposure data completeness of other adult women participants in HAPIN Trial 6

Table S3. Gravimetric sample validity 6

Table S4. Summary of valid personal exposure to $PM_{2.5}$, BC, and CO of adult women aged 40-79 by country and study group. 7

Table S5. Summary of personal exposure to $PM_{2.5}$ of other adult women participants by IRC and study group 8
Table S6. Summary of personal exposure to BC of other adult women participants by IRC and study group 9
Table S7. Summary of personal exposure to CO of other adult women participants by IRC and study group 10
Table S8. Correlations (Spearman's ρ) between pollutants by stove type and IRC 11
Table S9. Correlations (Spearman's ρ) of personal exposure to $PM_{2.5}$, BC, and CO between measurement rounds 11
Table S10. Percent decreases in $PM_{2.5}$, BC, and CO exposure associated with LPG intervention (trial-wide) 12
Table S12. Comparison of personal exposures to $PM_{2.5}$ between pregnant and non-pregnant adult women in the same households by arm and study period 14
Table S13. Comparison of personal exposures to BC between pregnant and non-pregnant adult women in the same households by arm and study period 15
Table S14. Comparison of personal exposures to CO between pregnant and non-pregnant adult women in the same households by arm and study period 16
Table S15. Correlation (Spearman's ρ) of personal measures of exposure between non-pregnant and pregnant adult women by pollutant, stove-type, IRC, and study period 17
Table S16. Personal exposures from comparable household air pollution studies 18
Table S17. Household and participants characteristics at baseline, by IRC and study arm 20
Table S18. PM2.5 exposures by specific household / participants characteristics for each country, study arm, and study period 23
Table S19. CO exposures by specific household / participants characteristics for each country, study arm, and study period 26
Table S20. BC exposures by specific household / participants characteristics for each country, study arm, and study period 29
FIGURES 32
Figure S1. Boxplot of personal exposure to $PM_{2.5}$ among non-pregnant adult women participants by IRC, study group, and visit. 32
Figure S2. Boxplot of personal exposure to BC among non-pregnant adult women participants by IRC, study group, and visit. 33
Figure S3. Boxplot of personal exposure to CO among non-pregnant adult women participants by IRC, study group, and visit. 34
Figure S4. Correlation between log_{10}-transformed $PM_{2.5}$ and CO exposure by stove type and IRC 35
Figure S5. Correlation between log_{10}-transformed BC and CO exposure by stove type and IRC 35
Figure S6. Correlation between log_{10}-transformed $PM_{2.5}$ and BC exposure by stove type and IRC 36
Figure S7. Trends in personal $PM_{2.5}$ exposure among the HAPIN non-pregnant adult women participants in each IRC. 38
Figure S8. Estimated effects of the HAPIN LPG stove and fuel intervention on BC exposure. 39
Figure S9. Estimated effects of the HAPIN LPG stove and fuel intervention on CO exposure. 39
Figure S10. Trial-wide and IRC-specific comparison of personal exposures to PM2.5 between pregnant and non-pregnant adult women in the same households by arm and study period 40
Figure S11. Trial-wide and IRC-specific comparison of personal exposures to BC between pregnant and non-pregnant adult women in the same households by arm and study period 40
Figure S12. Trial-wide and IRC-specific comparison of personal exposures to CO between pregnant and non-pregnant adult women in the same households by arm and study period 41
Figure S13. Baseline Measurements by Calendar Date. 42

Figure S14. Pregnancy Period Measurements by Calendar Date. 42
Figure S15. Post-birth Measurements by Calendar Date. 43

TABLES

Table S1. Selected characteristics of households and participants with and without $PM_{2.5}$ exposure measurements by visit and study arm

During Pregnancy	Baseline				P1				P2			
	Control		Intervention		Control		Intervention		Control		Intervention	
	Missing	Not Missing	Missing	Not Missing	Missing	Not Missing	Missing	Not Missing	Missing	Not Missing	Missing	Not Missing
N	17	192	19	190	33	160	34	164	36	148	43	149
**Education**	n (%)											
No formal education or Primary school incomplete	14 (82.4%)	153 (79.7%)	13 (68.4%)	155 (81.6%)	27 (81.8%)	129 (80.6%)	28 (82.4%)	133 (81.1%)	28 (77.8%)	119 (80.4%)	34 (79.1%)	119 (79.9%)
Primary school complete or Secondary school incomplete	2 (11.8%)	29 (15.1%)	6 (31.6%)	28 (14.7%)	4 (12.1%)	22 (13.8%)	5 (14.7%)	26 (15.9%)	5 (13.9%)	21 (14.2%)	8 (18.6%)	25 (16.8%)
Secondary school complete or Vocational or Some college or university	0	7 (3.6%)	0	3 (1.6%)	1 (3.0%)	6 (3.8%)	1 (2.9%)	2 (1.2%)	2 (5.6%)	5 (3.4%)	0	2 (1.3%)
Missing	1 (5.9%)	3 (1.6%)	0	4 (2.1%)	1 (3.0%)	3 (1.9%)	0	3 (1.8%)	1 (2.8%)	3 (2.0%)	1 (2.3%)	3 (2.0%)
**Diet Diversity Score**	n (%)											
High (<5)	3 (17.6%)	16 (8.3%)	6 (31.6%)	11 (5.8%)	4 (12.1%)	14 (8.8%)	5 (14.7%)	11 (6.7%)	5 (13.9%)	11 (7.4%)	3 (7.0%)	13 (8.7%)
Medium (4-5)	9 (52.9%)	57 (29.7%)	6 (31.6%)	49 (25.8%)	10 (30.3%)	49 (30.6%)	10 (29.4%)	39 (23.8%)	16 (44.4%)	42 (28.4%)	14 (32.6%)	39 (26.2%)
Low (0-3)	5 (29.4%)	119 (62.0%)	7 (36.8%)	130 (68.4%)	19 (57.6%)	97 (60.6%)	19 (55.9%)	114 (69.5%)	15 (41.7%)	95 (64.2%)	26 (60.5%)	97 (65.1%)
**Food Insecurity Score**	n (%)											
Mild (1-3)	1 (5.9%)	63 (32.8%)	4 (21.1%)	59 (31.1%)	15 (45.5%)	45 (28.1%)	13 (38.2%)	47 (28.7%)	14 (38.9%)	41 (27.7%)	12 (27.9%)	49 (32.9%)
Moderate/Severe (4-8)	3 (17.6%)	35 (18.2%)	0	19 (10.0%)	9 (27.3%)	26 (16.2%)	2 (5.9%)	15 (9.1%)	6 (16.7%)	30 (20.3%)	1 (2.3%)	15 (10.1%)
None (0)	12 (70.6%)	91 (47.4%)	14 (73.7%)	111 (58.4%)	9 (27.3%)	85 (53.1%)	19 (55.9%)	100 (61.0%)	15 (41.7%)	74 (50.0%)	30 (69.8%)	83 (55.7%)
Missing	1 (5.9%)	3 (1.6%)	1 (5.3%)	1 (0.5%)	0	4 (2.5%)	0	2 (1.2%)	1 (2.8%)	3 (2.0%)	0	2 (1.3%)
**Age, Mean (SD)**	52.3 (7.41)	51.8 (7.5)	51.8 (8.14)	52.3 (8.27)	51.3 (7.57)	51.8 (7.48)	50.8 (7.31)	52.5 (8.4)	50.4 (8.25)	52 (7.06)	50.6 (6.13)	52.5 (8.7)
**BMI, Mean (SD)**	25.7 (5.35)	25.5 (5.15)	26.8 (5.76)	25 (4.99)	25.3 (5.65)	25.4 (5.03)	26 (5)	25 (5.12)	27.3 (4.03)	24.6 (5.09)	25.5 (4.9)	25.3 (4.97)

Post-birth	B1				B2				B4			
	Control		Intervention		Control		Intervention		Control		Intervention	
	Missing	Not Missing	Missing	Not Missing	Missing	Not Missing	Missing	Not Missing	Missing	Not Missing	Missing	Not Missing
N	59	114	65	124	53	118	65	116	56	119	62	128
**Education**	n (%)											
No formal education or Primary school incomplete	43 (72.9%)	96 (84.2%)	46 (70.8%)	105 (84.7%)	38 (71.7%)	98 (83.1%)	51 (78.5%)	93 (80.2%)	42 (75.0%)	97 (81.5%)	47 (75.8%)	105 (82.0%)
Primary school complete or Secondary school incomplete	14 (23.7%)	11 (9.6%)	17 (26.2%)	15 (12.1%)	13 (24.5%)	14 (11.9%)	11 (16.9%)	20 (17.2%)	10 (17.9%)	16 (13.4%)	14 (22.6%)	18 (14.1%)

Secondary school complete or Vocational or Some college or university	2 (3.4%)	5 (4.4%)	2 (3.1%)	1 (0.8%)	1 (1.9%)	5 (4.2%)	3 (4.6%)	0	3 (5.4%)	3 (2.5%)	1 (1.6%)	2 (1.6%)
Missing	0	2 (1.8%)	0	3 (2.4%)	1 (1.9%)	1 (0.8%)	0	3 (2.6%)	1 (1.8%)	3 (2.5%)	0	3 (2.3%)
**Diet Diversity**						n (%)						
High (<5)	7 (11.9%)	7 (6.1%)	6 (9.2%)	10 (8.1%)	10 (18.9%)	5 (4.2%)	5 (7.7%)	9 (7.8%)	7 (12.5%)	9 (7.6%)	9 (14.5%)	7 (5.5%)
Medium (4-5)	23 (39.0%)	28 (24.6%)	25 (38.5%)	23 (18.5%)	19 (35.8%)	34 (28.8%)	27 (41.5%)	23 (19.8%)	22 (39.3%)	32 (26.9%)	23 (37.1%)	28 (21.9%)
Low (0-3)	29 (49.2%)	79 (69.3%)	34 (52.3%)	91 (73.4%)	24 (45.3%)	79 (66.9%)	33 (50.8%)	84 (72.4%)	27 (48.2%)	78 (65.5%)	30 (48.4%)	93 (72.7%)
**Food Insecurity**						n (%)						
Mild (1-3)	22 (37.3%)	31 (27.2%)	18 (27.7%)	38 (30.6%)	20 (37.7%)	34 (28.8%)	21 (32.3%)	33 (28.4%)	17 (30.4%)	36 (30.3%)	20 (32.3%)	38 (29.7%)
Moderate/Severe (4-8)	9 (15.3%)	23 (20.2%)	5 (7.7%)	9 (7.3%)	10 (18.9%)	21 (17.8%)	4 (6.2%)	10 (8.6%)	17 (30.4%)	14 (11.8%)	7 (11.3%)	9 (7.0%)
None (0)	27 (45.8%)	58 (50.9%)	41 (63.1%)	76 (61.3%)	22 (41.5%)	61 (51.7%)	38 (58.5%)	73 (62.9%)	19 (33.9%)	68 (57.1%)	34 (54.8%)	80 (62.5%)
Missing	1 (1.7%)	2 (1.8%)	1 (1.5%)	1 (0.8%)	1 (1.9%)	2 (1.7%)	2 (3.1%)	0	3 (5.4%)	1 (0.8%)	1 (1.6%)	1 (0.8%)
**Age, Mean (SD)**	50.9 (8.05)	52.3 (7.2)	51.4 (8.05)	52.5 (8.24)	52.3 (7.73)	51.6 (7.12)	52.2 (8.07)	51.7 (7.92)	50.7 (7.92)	52.4 (7.25)	51.3 (7.29)	51.8 (8.3)
**BMI, Mean (SD)**	26.4 (5.09)	24.6 (4.84)	26.2 (5.15)	24.8 (5.01)	26.9 (4.33)	24.6 (5.15)	25.3 (4.51)	24.9 (5.26)	26 (5.04)	24.7 (4.87)	26.3 (5.19)	25 (4.83)

Table S2. Overall exposure data completeness of other adult women participants in HAPIN Trial

Visit	IRC	Enrolled	Exposure Visit Made	Exposure Data Available	% Total Enrolled with Exposure Measurement	% Total Visit Made with Exposure
BL	Guatemala	138	138	130	94.2	94.2
	India	104	104	90	86.5	86.5
	Peru	133	132	107	80.5	81.1
	Rwanda	43	43	32	74.4	74.4
P1	Guatemala	138	125	112	81.2	89.6
	India	104	96	86	82.7	89.6
	Peru	133	106	87	65.4	82.1
	Rwanda	43	33	30	69.8	90.9
P2	Guatemala	138	120	106	76.8	88.3
	India	104	81	67	64.4	82.7
	Peru	133	89	74	55.6	83.1
	Rwanda	43	37	29	67.4	78.4
B1	Guatemala	138	100	90	65.2	90
	India	104	84	63	60.6	75
	Peru	133	63	49	36.8	77.8
	Rwanda	43	26	20	46.5	76.9
B2	Guatemala	138	87	74	53.6	85.1
	India	104	84	70	67.3	83.3
	Peru	133	70	53	39.8	75.7
	Rwanda	43	31	21	48.8	67.7
B4	Guatemala	138	100	85	61.6	85
	India	104	91	80	76.9	87.9
	Peru	133	70	46	34.6	65.7
	Rwanda	43	22	14	32.6	63.6

Note: BL - baseline, P1 – prenatal visit 1, P2 – prenatal visit 2, B1 – post-birth visit 1, B2 – post-birth visit 2, and B4 – post-birth visit 3.

Table S3. Gravimetric sample validity

IRC	Valid Gravimetric Samples, N (%)	Valid Nephelometric Samples, N (%)	Valid Samples, N (%)	% Flow Issues	% Pressure Issues	% Duration Issues	% Filter Damaged
Guatemala	556 (85.4%)	616 (94.6%)	617 (94.8%)	14 (2.2%)	6 (0.9%)	30 (4.6%)	10 (1.5%)
India	454 (86.1%)	472 (89.6%)	474 (89.9%)	9 (1.7%)	10 (1.9%)	47 (8.9%)	4 (0.8%)
Peru	435 (82.7%)	468 (89.0%)	470 (89.4%)	16 (3%)	12 (2.3%)	52 (9.9%)	16 (3%)
Rwanda	152 (82.6%)	165 (89.7%)	170 (92.4%)	4 (2.2%)	2 (1.1.%)	14 (7.6%)	11 (6%)

Table S4. Summary of valid personal exposure to $PM_{2.5}$, BC, and CO of adult women aged 40-79 by country and study group.

	$PM_{2.5}$ Exposure (μg/m³)		BC Exposure (μg/m³)		CO Exposure (ppm)	
	**Control**	**Intervention**	**Control**	**Intervention**	**Control**	**Intervention**
**Post-intervention Visit 1**						
N	160	164	148	155	148	152
Average (SD)	110.2 (129.5)	36.8 (50.1)	10.2 (7.7)	3.1 (2.5)	2.2 (2.8)	0.8 (1.8)
Range	10.2 - 946.4	10 - 464.2	1 - 40.1	0.9 - 16.9	0 - 17	0 - 14.5
Median (IQR)	64.8 (32.9-143.5)	21.8 (15.1-35.6)	8.8 (4.4-13.8)	1.7 (1.5-3.6)	1.2 (0.4-2.7)	0.2 (0-0.7)
**Post-intervention Visit 2**						
N	148	149	131	144	136	137
Average (SD)	104.9 (121.1)	37.6 (63)	10.8 (10.8)	3.9 (3.8)	2 (3.4)	1 (2)
Range	10 - 793.8	10 - 725	1 - 83.9	0.9 - 28.5	0 - 24.2	0 - 13.7
Median (IQR)	66.2 (34.2-110.6)	23.4 (15.2-41.8)	8.6 (4.3-13.5)	2.6 (1.6-4.7)	1 (0.3-2.2)	0.2 (0-1)
**Post-intervention Visit 3**						
N	114	124	100	113	98	114
Average (SD)	102.1 (104.2)	37 (68.4)	12 (10.9)	4.8 (7.9)	1.7 (2.3)	1.3 (3.2)
Range	10 - 520.4	11.2 - 718.3	1.4 - 76	0.9 - 70.6	0 - 15.5	0 - 18.9
Median (IQR)	72.2 (37.4-119.4)	24.4 (14.4-35.4)	9.7 (4.8-15.2)	2.4 (1.6-4.4)	0.9 (0.2-2.5)	0.2 (0-0.9)
**Post-intervention Visit 4**						
N	118	116	110	107	117	107
Average (SD)	121.4 (194.5)	43.2 (96.3)	10 (7.1)	3.6 (4.1)	2.1 (3.1)	1.5 (3)
Range	10.6 - 1447.6	11 - 969.1	1 - 31.9	0.9 - 24.4	0 - 16	0 - 17.8
Median (IQR)	70.6 (32.2-123.7)	18.7 (13.6-38.2)	8.5 (4.5-13.6)	1.7 (1.5-3.5)	0.8 (0.2-2.4)	0.3 (0-1.2)
**Post-intervention Visit 5**						
N	119	128	94	112	105	116
Average (SD)	114.1 (156.3)	37.1 (35.9)	9.4 (8)	5 (6.7)	1.9 (3.5)	1 (2.4)
Range	11 - 1062.3	10 - 227	1 - 56.4	0.9 - 53.3	0 - 28.2	0 - 15.2
Median (IQR)	54.4 (28.4-133.3)	24.8 (13.4-43.7)	7.9 (3.8-12.8)	2.9 (1.6-5.2)	0.9 (0.3-2.2)	0.2 (0-0.6)

Table S5. Summary of personal exposure to $PM_{2.5}$ of other adult women participants by IRC and study group

$PM_{2.5}$ Exposure (µg/m³)	Guatemala		India		Peru		Rwanda		Overall	
	Control	Intervention	Control	Intervention	Control	Intervention	Control	Intervention	Control	Intervention
Baseline										
N	64	70	47	46	58	56	23	18	192	190
Average (SD)	130.4 (108.3)	154 (139.9)	101 (93)	120.1 (152.3)	100.4 (104.6)	111.3 (135.3)	118.1 (77)	60.5 (39.3)	112.7 (100.4)	124.4 (137.5)
Range	13.7 - 660.8	17.3 - 803.4	10 - 531.4	10 - 759.6	12.5 - 427.4	12.5 - 698.1	14.7 - 312.1	19.8 - 166.2	10 - 660.8	10 - 803.4
Median (IQR)	107.2 (69.6-140.5)	115.6 (75-178.5)	75.2 (44.1-123.8)	70.9 (40.3-128.4)	67.1 (27.7-124.3)	59 (29.3-122.6)	101.8 (56.6-160.3)	51.9 (32.4-78.2)	89.4 (44.2-135.6)	79.8 (43-148.5)
Post-intervention Visit 1										
N	56	59	41	46	47	47	16	12	160	164
Average (SD)	145.5 (161.2)	31.6 (31.2)	96.9 (104.9)	35.8 (66.1)	82.9 (111.2)	44.6 (56.3)	100.4 (87.1)	35.1 (21.1)	110.2 (129.5)	36.8 (50.1)
Range	11.8 - 946.4	10 - 176.1	10.2 - 407.3	10.1 - 464.2	13.1 - 531.2	12.4 - 289.8	23 - 318.1	20 - 92.2	10.2 - 946.4	10 - 464.2
Median (IQR)	96.9 (55.4-173.5)	24.6 (11.9-30.4)	49.1 (32.3-122.5)	19.5 (17.7-34)	41.3 (14.9-97.4)	15.7 (14.8-53.1)	73.1 (51.4-99.3)	28.3 (20.3-41.6)	64.8 (32.9-143.5)	21.8 (15.1-35.6)
Post-intervention Visit 2										
N	52	61	40	32	35	44	21	12	148	149
Average (SD)	140.4 (147.2)	37.1 (35.6)	108.2 (106.1)	25.8 (12.2)	65.7 (108.1)	48.5 (107.4)	76.3 (62.5)	31.9 (11.3)	104.9 (121.1)	37.6 (63)
Range	11.8 - 793.8	10 - 222.7	10 - 396.9	10 - 59.7	12.3 - 541.3	12.5 - 725	30.5 - 295.6	20.2 - 49.1	10 - 793.8	10 - 725
Median (IQR)	83.6 (52.4-164.6)	24.6 (11.9-46.5)	72.9 (38-114.2)	18 (17.8-35.6)	29.9 (15-59.3)	21.7 (14.8-46.8)	61.1 (43.7-72.3)	29.3 (21.5-42)	66.2 (34.2-110.6)	23.4 (15.2-41.8)
Post-intervention Visit 3										
N	45	47	33	36	21	32	15	9	114	124
Average (SD)	134.3 (112.6)	35.6 (31.1)	87.2 (93.8)	46.4 (116.4)	47 (44.5)	28.4 (39.9)	115.6 (127.2)	37.5 (11.3)	102.1 (104.2)	37 (68.4)
Range	21.5 - 520.4	11.2 - 171.4	10 - 412.3	13.1 - 718.3	12.7 - 188.5	12.5 - 233.6	18.8 - 508.4	20.8 - 55.5	10 - 520.4	11.2 - 718.3
Median (IQR)	105.7 (59.5-169.8)	29.1 (12.6-39.8)	66.8 (26.1-94.2)	19.4 (17.6-32.4)	27.8 (15.3-60.4)	14.8 (14.2-27.5)	78 (50.4-105.4)	34.6 (33.1-42.2)	72.2 (37.4-119.4)	24.4 (14.4-35.4)
Post-intervention Visit 4										
N	42	33	33	38	25	37	18	8	118	116
Average (SD)	115 (101.9)	40.9 (37.6)	116.6 (245.8)	27.1 (27)	151.6 (284)	60.9 (164.2)	103.3 (77.5)	46.9 (18.8)	121.4 (194.5)	43.2 (96.3)
Range	11.8 - 458	11.1 - 162.9	11.8 - 1447.6	13.4 - 138.3	10.6 - 1065.1	11 - 969.1	23.5 - 355.6	19.8 - 72.8	10.6 - 1447.6	11 - 969.1
Median (IQR)	80.3 (53-150.6)	27.8 (15.8-41.8)	67.9 (32.3-103.1)	17.9 (14.6-27.6)	42.8 (15.2-106.9)	15.1 (12.6-34.5)	88.7 (54.8-120.5)	49.4 (32.1-60.1)	70.6 (32.2-123.7)	18.7 (13.6-38.2)
Post-intervention Visit 5										
N	43	45	41	41	24	36	11	6	119	128
Average (SD)	116.5 (134.3)	34.9 (32.9)	104.1 (100.7)	49.9 (43.4)	143.8 (269.1)	23.5 (26.2)	77.8 (39.9)	47.7 (22.2)	114.1 (156.3)	37.1 (35.9)
Range	12 - 598.9	11 - 137.5	13.2 - 434.6	10 - 227	11 - 1062.3	10.5 - 136	39.4 - 158.3	14 - 68.2	11 - 1062.3	10 - 227
Median (IQR)	57.1 (34.5-136.2)	22.6 (14.4-34.9)	56.5 (33.5-131.2)	39.2 (17.9-55.9)	19.9 (13.7-81.6)	14.1 (11-23.2)	67.6 (53.2-80.4)	57 (33.6-62.5)	54.4 (28.4-133.3)	24.8 (13.4-43.7)

Table S6. Summary of personal exposure to BC of other adult women participants by IRC and study group

BC Exposure (µg/m³)	Guatemala		India		Peru		Rwanda		Overall	
	Control	Intervention	Control	Intervention	Control	Intervention	Control	Intervention	Control	Intervention
Baseline										
N	54	66	46	44	48	47	18	10	166	167
Average (SD)	12.3 (5.8)	13.4 (6.6)	14.2 (11.6)	12.6 (10.8)	11.6 (14)	14.7 (16.6)	12.3 (4.5)	6.4 (3.1)	12.6 (10.3)	13.1 (11.3)
Range	1.1 - 29	4.3 - 46.8	1.6 - 69.2	1.4 - 47.8	1.4 - 72.3	1.3 - 93.3	3.5 - 20.1	2.8 - 12.3	1.1 - 72.3	1.3 - 93.3
Median (IQR)	11.5 (8.8-15.7)	12.5 (9.8-15.3)	11.5 (6.9-18)	7.9 (3.8-20.9)	6.3 (2.1-15.4)	10.6 (3.8-16.5)	11 (10-15.8)	5.8 (4.1-8.5)	10.7 (6.2-16.1)	10.9 (6.7-16)
Post-intervention Visit 1										
N	54	56	40	45	38	44	16	10	148	155
Average (SD)	11.8 (6.2)	4 (3.2)	10.9 (9.5)	2.7 (2)	7.1 (6.6)	1.9 (1.5)	10.9 (8.1)	4.2 (1.6)	10.2 (7.7)	3.1 (2.5)
Range	1 - 33.1	0.9 - 16.9	1.6 - 40.1	1.4 - 9.5	1.3 - 24.8	1.3 - 10.6	2.9 - 37.7	2.8 - 6.6	1 - 40.1	0.9 - 16.9
Median (IQR)	11.1 (8-13.3)	2.9 (1.7-5.5)	7.1 (4-16.8)	1.6 (1.6-3.2)	4.1 (1.5-11.3)	1.5 (1.5-1.5)	8.6 (7.1-12.9)	3.7 (2.9-5.4)	8.8 (4.4-13.8)	1.7 (1.5-3.6)
Post-intervention Visit 2										
N	47	60	38	32	29	40	17	12	131	144
Average (SD)	11.6 (5.4)	5.3 (4.9)	13.3 (11.7)	3.3 (2.9)	8.3 (16.7)	2.3 (2)	6.9 (2.8)	4 (1.4)	10.8 (10.8)	3.9 (3.8)
Range	1 - 26.5	0.9 - 28.5	1.6 - 59.9	1.6 - 16.1	1.4 - 83.9	1.4 - 11.9	3.4 - 15.9	2.8 - 6.6	1 - 83.9	0.9 - 28.5
Median (IQR)	11.3 (8.5-14)	3.5 (2.4-7)	10.2 (5.2-18)	2.3 (1.6-3.8)	1.5 (1.4-6.4)	1.5 (1.5-2.3)	6.2 (5.3-8.1)	3 (2.9-5.1)	8.6 (4.3-13.5)	2.6 (1.6-4.7)
Post-intervention Visit 3										
N	37	42	29	32	19	32	15	7	100	113
Average (SD)	14.2 (8.1)	5.7 (4.6)	13.3 (15.3)	4.1 (6.6)	7.3 (8.2)	4.4 (12.2)	10.3 (7.8)	3.6 (1.1)	12 (10.9)	4.8 (7.9)
Range	5.5 - 52.3	0.9 - 19.6	1.6 - 76	1.6 - 36.5	1.4 - 28.2	1.4 - 70.6	2.8 - 33.3	2.9 - 5.9	1.4 - 76	0.9 - 70.6
Median (IQR)	12.5 (9.8-17.4)	3.6 (2.5-9.3)	8 (4.4-15.4)	1.6 (1.6-3.6)	2.8 (1.5-10.3)	1.5 (1.5-2.2)	8.8 (4.6-11.7)	3 (3-3.7)	9.7 (4.8-15.2)	2.4 (1.6-4.4)
Post-intervention Visit 4										
N	36	28	32	37	25	34	17	8	110	107
Average (SD)	11.4 (6)	6.4 (6.3)	10.1 (8)	2.1 (1.3)	7.5 (8.4)	2.5 (2.7)	10.3 (4.6)	5.2 (2)	10 (7.1)	3.6 (4.1)
Range	1 - 27.7	0.9 - 24.4	1.4 - 31.7	1.4 - 8.9	1.4 - 31.9	1.4 - 13.2	3.7 - 17.9	2.8 - 8.9	1 - 31.9	0.9 - 24.4
Median (IQR)	11.3 (7.8-13.8)	3.6 (2.2-7.5)	7.7 (4.7-12.4)	1.6 (1.6-1.8)	3.7 (1.5-10)	1.5 (1.5-1.8)	10.7 (5.8-13.7)	4.7 (4.3-5.7)	8.5 (4.5-13.6)	1.7 (1.5-3.5)
Post-intervention Visit 5										
N	29	38	38	40	17	29	10	5	94	112
Average (SD)	10.1 (5.7)	5.5 (4.7)	11.1 (10.4)	5.7 (9.7)	4.8 (4.9)	3.1 (3.7)	9.3 (4.3)	5.2 (1.8)	9.4 (8)	5 (6.7)
Range	1 - 18.9	0.9 - 17.4	1.6 - 56.4	1.4 - 53.3	1.4 - 15.2	1.4 - 20.4	4.1 - 17.6	2.9 - 7.8	1 - 56.4	0.9 - 53.3
Median (IQR)	9.9 (5.5-15.7)	3.4 (2-7.8)	8.4 (4-14.5)	2.3 (1.6-5.2)	1.7 (1.5-7.9)	2.3 (1.5-2.9)	8.4 (6.3-12.2)	5.1 (4.3-6.1)	7.9 (3.8-12.8)	2.9 (1.6-5.2)

Table S7. Summary of personal exposure to CO of other adult women participants by IRC and study group

CO Exposure (ppm)	Guatemala		India		Peru		Rwanda		Overall	
	Control	Intervention	Control	Intervention	Control	Intervention	Control	Intervention	Control	Intervention
Baseline										
N	58	66	45	43	45	48	21	17	169	174
Average (SD)	1.9 (2)	1.7 (1.5)	1.2 (1.3)	2 (3.8)	4 (3.8)	4 (6.3)	1.1 (1)	1.5 (2.4)	2.2 (2.7)	2.4 (4.1)
Range	0.1 - 9.5	0 - 7.5	0 - 6.3	0 - 21.4	0 - 18.1	0 - 38.7	0.1 - 3.8	0 - 8.9	0 - 18.1	0 - 38.7
Median (IQR)	1.3 (0.6-2.5)	1.4 (0.5-2.6)	0.6 (0.2-1.7)	0.7 (0.2-2.1)	2.9 (1.5-5.8)	2 (0.9-4.3)	0.7 (0.3-1.7)	0.3 (0.1-1.3)	1.3 (0.5-2.9)	1.4 (0.4-2.7)
Post-intervention Visit 1										
N	56	55	41	47	35	40	16	10	148	152
Average (SD)	2 (1.9)	0.6 (1)	2.4 (3.8)	0.3 (1.4)	2.7 (3)	1.7 (2.9)	1.1 (1)	0.4 (0.5)	2.2 (2.8)	0.8 (1.8)
Range	0 - 9.4	0 - 4.6	0 - 17	0 - 9.5	0 - 10.6	0 - 14.5	0 - 2.9	0 - 1.4	0 - 17	0 - 14.5
Median (IQR)	1.5 (0.6-2.8)	0.3 (0-0.6)	0.8 (0.1-2.5)	0 (0-0.1)	1.6 (0.3-3.9)	0.6 (0.2-1.9)	0.8 (0.2-1.8)	0.3 (0.1-0.4)	1.2 (0.4-2.7)	0.2 (0-0.7)
Post-intervention Visit 2										
N	50	54	36	35	31	39	19	9	136	137
Average (SD)	2.2 (3.3)	0.7 (0.9)	2.5 (4.4)	0.3 (1.2)	1.8 (2.8)	2 (2.7)	0.8 (1.3)	1.7 (4)	2 (3.4)	1 (2)
Range	0 - 20.7	0 - 3.6	0 - 24.2	0 - 6.7	0 - 13.9	0 - 13.7	0 - 4.9	0 - 12.3	0 - 24.2	0 - 13.7
Median (IQR)	1.3 (0.7-2.2)	0.2 (0-1)	1.4 (0.3-2.3)	0 (0-0.1)	1 (0.2-2.1)	0.7 (0.2-3.2)	0.2 (0.1-0.9)	0 (0-1)	1 (0.3-2.2)	0.2 (0-1)
Post-intervention Visit 3										
N	39	41	32	38	14	29	13	6	98	114
Average (SD)	1.8 (2)	0.5 (0.9)	1.6 (1.8)	0.4 (0.9)	2.8 (4.1)	3.6 (5.6)	1 (1.2)	1.5 (2)	1.7 (2.3)	1.3 (3.2)
Range	0.1 - 8.6	0 - 3.9	0 - 5.6	0 - 4.1	0 - 15.5	0 - 18.9	0.1 - 2.9	0 - 5.4	0 - 15.5	0 - 18.9
Median (IQR)	1.1 (0.3-2.4)	0.1 (0-0.5)	0.6 (0.2-2.5)	0.1 (0-0.2)	1.9 (0.2-2.7)	0.7 (0.4-4)	0.3 (0.1-2.5)	1 (0.2-1.5)	0.9 (0.2-2.5)	0.2 (0-0.9)
Post-intervention Visit 4										
N	41	31	36	39	22	27	18	10	117	107
Average (SD)	1.6 (1.7)	0.7 (1.8)	1.4 (1.8)	0.4 (1.1)	4.2 (5.2)	4 (4.6)	2 (3.3)	1.7 (1.4)	2.1 (3.1)	1.5 (3)
Range	0 - 6.5	0 - 10.1	0 - 6.4	0 - 4.7	0 - 16	0 - 17.8	0 - 13.5	0.2 - 4.8	0 - 16	0 - 17.8
Median (IQR)	0.9 (0.3-2.3)	0.1 (0-0.5)	0.7 (0.1-1.5)	0.1 (0-0.3)	2.3 (0.4-5.9)	3.4 (0.6-5.1)	0.5 (0.2-2.5)	1.1 (0.9-2)	0.8 (0.2-2.4)	0.3 (0-1.2)
Post-intervention Visit 5										
N	40	46	41	41	12	22	12	7	105	116
Average (SD)	1.6 (2.4)	0.6 (1.1)	1.6 (2.3)	0.7 (1.8)	4.9 (7.8)	3 (4.3)	0.9 (1)	0.2 (0.2)	1.9 (3.5)	1 (2.4)
Range	0 - 12.2	0 - 5.6	0 - 8.6	0 - 10.7	0 - 28.2	0 - 15.2	0 - 3.5	0 - 0.6	0 - 28.2	0 - 15.2
Median (IQR)	0.7 (0.2-2.3)	0.2 (0-0.5)	0.9 (0.1-2)	0 (0-0.3)	2.3 (0.8-5.7)	0.7 (0.4-4.2)	0.7 (0.3-1.2)	0.1 (0-0.2)	0.9 (0.3-2.2)	0.2 (0-0.6)

Table S8. Correlations (Spearman's ρ) between pollutants by stove type and IRC

	PM-BC		PM-CO		BC-CO	
**IRC**	**LPG**	**Traditional**	**LPG**	**Traditional**	**LPG**	**Traditional**
All	0.60	0.76	0.11	0.53	0.07	0.49
Guatemala	0.85	0.67	0.40	0.67	0.34	0.50
India	0.40	0.73	-0.02	0.59	0.17	0.55
Peru	0.34	0.78	0.03	0.56	0.13	0.61
Rwanda	0.77	0.85	0.01	0.53	-0.15	0.50

Note: Traditional stove includes measurements from the intervention group at baseline and all measurements from the control group. LPG stove includes all post-intervention measurements from the intervention group.

Table S9. Correlations (Spearman's ρ) of personal exposure to $PM_{2.5}$, BC, and CO between measurement rounds

Group 1	Group 2	Correlation Coefficient	p-value	Correlation Coefficient	p-value
PM2.5		*Control*		*Intervention*	
P1	BL	0.43	0.000	0.20	0.014
P2	BL	0.42	0.000	0.04	0.639
B1	BL	0.29	0.002	0.02	0.839
B2	BL	0.37	0.000	0.11	0.250
B4	BL	0.26	0.006	0.17	0.056
P2	P1	0.45	0.000	0.35	0.000
B1	P1	0.43	0.000	0.15	0.129
B2	P1	0.42	0.000	0.17	0.104
B4	P1	0.33	0.001	0.12	0.214
B1	P2	0.41	0.000	0.30	0.004
B2	P2	0.40	0.000	0.27	0.012
B4	P2	0.34	0.001	0.25	0.013
B2	B1	0.43	0.000	0.14	0.216
B4	B1	0.24	0.039	0.28	0.011
B4	B2	0.19	0.104	0.27	0.013
BC		*Control*		*Intervention*	
P1	BL	0.26	0.005	0.17	0.066
P2	BL	0.38	0.000	0.13	0.165
B1	BL	0.32	0.004	0.11	0.298
B2	BL	0.17	0.111	0.05	0.622
B4	BL	0.43	0.000	0.35	0.001
P2	P1	0.38	0.000	0.53	0.000
B1	P1	0.54	0.000	0.43	0.000
B2	P1	0.26	0.017	0.38	0.000
B4	P1	0.38	0.001	0.19	0.071
B1	P2	0.56	0.000	0.46	0.000
B2	P2	0.33	0.003	0.30	0.010
B4	P2	0.46	0.000	0.40	0.000
B2	B1	0.34	0.005	0.31	0.006
B4	B1	0.33	0.013	0.38	0.002
B4	B2	0.29	0.033	0.48	0.000

CO		Control		Intervention	
P1	BL	0.15	0.095	0.24	0.005
P2	BL	0.23	0.014	0.26	0.004
B1	BL	0.26	0.016	0.20	0.053
B2	BL	0.13	0.190	-0.04	0.689
B4	BL	0.18	0.084	-0.09	0.404
P2	P1	0.42	0.000	0.32	0.001
B1	P1	0.32	0.003	0.36	0.001
B2	P1	0.17	0.101	0.20	0.071
B4	P1	0.06	0.576	0.13	0.252
B1	P2	0.39	0.001	0.29	0.009
B2	P2	0.27	0.014	0.29	0.010
B4	P2	0.42	0.000	0.23	0.035
B2	B1	0.10	0.423	0.16	0.182
B4	B1	0.18	0.164	0.18	0.120
B4	B2	0.39	0.001	0.08	0.516

Note: BL - baseline, P1 – prenatal visit 1, P2 – prenatal visit 2, B1 – post-birth visit 1, B2 – post-birth visit 2, and B4 – post-birth visit 3.

For $PM_{2.5}$ in the control group, the correlations ranged from 0.19 to 0.45. Correlations for BC (range: 0.17 – 0.56) in the control group were similar to $PM_{2.5}$ but were weaker for CO (range: 0.06 – 0.42). Generally, the correlations between measurement rounds in the intervention group are weaker compared to that in the control group, even between the post-intervention measurement rounds. Among intervention households, correlations between all measurement rounds ranged from 0.02 – 0.35, 0.05 – 0.53, and -0.04 – 0.36 for $PM_{2.5}$, BC, and CO exposures, respectively. Among the consecutive visits, the two follow-up visits during pregnancy (P1 and P2) tend to have stronger correlations for all three pollutants in the intervention group. In the control group, stronger correlations are observed between the last follow-up visit during pregnancy (P2) and the first follow-up visit post-birth (B1).

Table S10. Percent decreases in $PM_{2.5}$, BC, and CO exposure associated with LPG intervention (trial-wide)

Model Type	Details	% Decrease in PM2.5 exposure	% Decrease in BC exposure	% Decrease in CO exposure
		Estimate (CI)	Estimate (CI)	Estimate (CI)
Between Groups	–	59 (55, 63)	60 (56, 65)	73 (65, 80)
Before and After	Control	20 (9, 29)	25 (15, 33)	21 (-5, 40)
	Intervention	67 (63, 71)	70 (67, 74)	79 (70, 85)
Comparison of Changes	Overall	59 (51, 65)	61 (54, 67)	74 (60, 83)
	Visit P1	61 (51, 69)	67 (60, 73)	77 (60, 87)
	Visit P2	58 (67, 47)	60 (68, 50)	81 (89, 65)
	Visit B1	61 (69, 49)	65 (72, 56)	75 (87, 53)
	Visit B2	60 (69, 48)	63 (70, 53)	63 (80, 31)
	Visit B4	51 (62, 37)	47 (58, 33)	70 (84, 43)

Table S11. Comparison of personal exposures to $PM_{2.5}$, BC, and CO between non-pregnant and pregnant adult women in the same households by arm and study period.

	**PM2.5 (µg/m³)**				**BC (µg/m³)**				**CO (ppm)**			
	**Control**		**Intervention**		**Control**		**Intervention**		**Control**		**Intervention**	
	**OAW**	**PW**	**OAW**	**PW**	**OAW**	**PW**	**OAW**	**PW**	**OAW**	**PW**	**OAW**	**PW**
Baseline												
N	180	180	174	174	148	148	152	152	151	151	151	151
Average (SD)	110.7 (93.7)	96.8 (92.3)	148.3 (242.4)	131.6 (157.7)	12.7 (10.3)	11.8 (9.6)	12.5 (9.2)	13.6 (11.4)	2.2 (2.7)	1.8 (2.5)	2.3 (4.1)	2.6 (4.4)
Range	10 - 531.4	10 - 568.1	10 - 2063.2	10 - 1382.2	1.1 - 72.3	1.5 - 66.1	1.4 - 49.3	1.4 - 77.2	0 - 18.1	0 - 16.8	0 - 38.7	0 - 32.4
Median (IQR)	89.3 (44.3-135.6)	72.6 (35.5-116.1)	83.5 (43.5-159.2)	83.9 (42.9-159.6)	11 (6.2-16.1)	10.5 (6.4-14.5)	10.9 (6.7-15.7)	11.2 (6.3-16.6)	1.4 (0.5-2.9)	0.9 (0.4-2.1)	1.4 (0.4-2.7)	1.3 (0.4-2.8)
Pregnancy												
N	175	175	177	177	166	166	172	172	159	159	173	173
Average (SD)	111.3 (136.5)	110.4 (132.2)	49.1 (117.3)	25.7 (15.1)	10.5 (8.6)	11 (8.3)	3.5 (2.6)	3.1 (2.5)	2 (2.9)	2.1 (3.5)	0.9 (1.9)	0.5 (1)
Range	10.2 - 1041.1	11.8 - 1116.9	10 - 1271	10 - 102.4	1 - 83.9	1.3 - 65.3	0.9 - 16.6	0.9 - 19.1	0 - 20.7	0 - 27.6	0 - 14.1	0 - 6.7
Median (IQR)	69.7 (37.5-131.4)	74.5 (38.6-131)	26.6 (16.6-42.1)	20.6 (14.8-32.6)	9.5 (4.8-13.9)	10.2 (5.2-14.2)	2.7 (1.6-4.6)	2.3 (1.5-4.1)	1.2 (0.4-2.6)	1 (0.4-2.2)	0.2 (0-0.9)	0.2 (0-0.6)
Post Birth												
N	163	163	164	164	109	109	123	123	150	150	148	148
Average (SD)	117.9 (164.9)	91.5 (120)	56.3 (164.7)	49.9 (185.6)	9.9 (7.7)	8.6 (10.1)	3.9 (6)	3 (4)	2 (2.6)	1.7 (2.3)	1.3 (2.6)	0.7 (1.4)
Range	10.5 - 1551.7	10.8 - 910.8	10 - 1905.2	10.4 - 2240.5	1.4 - 49.9	1.4 - 91.2	0.9 - 53.3	0.9 - 41.1	0 - 15.8	0 - 16.7	0 - 17.8	0 - 12.4
Median (IQR)	75.6 (36.4-126.8)	54.9 (28.9-109)	26.1 (14.9-43.2)	20.3 (14.9-30.3)	8.1 (4.3-13.6)	5.9 (2.6-11.7)	2 (1.6-3.8)	1.6 (1.5-3)	1.2 (0.4-2.7)	0.8 (0.3-2.2)	0.3 (0.1-1.3)	0.2 (0-0.7)

Table S12. Comparison of personal exposures to $PM_{2.5}$ between pregnant and non-pregnant adult women in the same households by arm and study period

PM2.5 Exposure (µg/m³)	Guatemala				India				Peru				Rwanda				Overall			
	Control		Intervention		Control		Intervention		Control		Intervention		Control		Intervention		Control		Intervention	
	OAW	MOM	OAW	MOM	OAW	MOM	OAW	MOM	OAW	MOM	OAW	MOM	OAW	MOM	OAW	MOM	OAW	MOM	OAW	MOM
**Baseline**																				
N	63	63	68	68	43	43	41	41	51	51	49	49	23	23	16	16	180	180	174	174
Average (SD)	125.4 (86.7)	113.3 (92.6)	156.6 (141.2)	139.4 (95.8)	99.4 (95.9)	111.8 (113.1)	118.4 (146.5)	153.4 (245.4)	95.3 (97.8)	61.1 (66.7)	148.4 (292.8)	105.2 (143.9)	125.6 (96.4)	102.6 (80.3)	189.9 (502.6)	123.1 (121.7)	110.7 (93.7)	96.8 (92.3)	148.3 (242.4)	131.6 (157.7)
Range	13.7 - 471.8	10 - 374.7	17.3 - 803.4	10 - 429.2	10 - 531.4	11.5 - 568.1	10 - 759.6	10 - 1382.2	12.5 - 427.4	12.5 - 310.7	12.5 - 1916.7	12.6 - 788	14.7 - 458.2	20.5 - 405.8	19.8 - 2063.2	20.8 - 399.3	10 - 531.4	10 - 568.1	10 - 2063.2	10 - 1382.2
Median (IQR)	108.5 (73.1-145)	83.1 (49.1-155.9)	121 (76.8-181.4)	130.7 (66.8-179.9)	69.6 (38.5-123.8)	76 (54.6-122.3)	72.1 (40.7-133.9)	83.6 (46.1-128.6)	60.3 (28.1-123.9)	35.4 (14.9-82.6)	59.3 (24.6-123.2)	51.4 (21.9-118.9)	101.8 (61-160.3)	84.2 (48.7-134)	46 (29.3-83.4)	52.4 (43.4-170.5)	89.3 (44.3-135.6)	72.6 (35.5-116.1)	83.5 (43.5-159.2)	83.9 (42.9-159.6)
**Pregnancy**																				
N	60	60	62	62	44	44	48	48	49	49	51	51	22	22	16	16	175	175	177	177
Average (SD)	158.5 (191)	127.2 (105.2)	34.9 (25.4)	26.2 (14.9)	105.1 (94.4)	122.7 (100.9)	50.4 (120.9)	29.1 (15.6)	73.7 (91.4)	88.7 (195)	69.6 (181.7)	19.5 (13.9)	78.4 (61.2)	88.4 (56.1)	35.5 (19.1)	33 (11.6)	111.3 (136.5)	110.4 (132.2)	49.1 (117.3)	25.7 (15.1)
Range	11.8 - 1041.1	11.8 - 513.7	10 - 120.2	10 - 62.6	10.2 - 375.7	26.1 - 506.1	10.1 - 745.6	11.7 - 87	13.1 - 531.2	12.5 - 1116.9	12.4 - 1271	12.1 - 102.4	23 - 295.6	37.2 - 209	14.6 - 92.2	12.5 - 55.1	10.2 - 1041.1	11.8 - 1116.9	10 - 1271	10 - 102.4
Median (IQR)	99.4 (58.7-178)	91.1 (52.7-155.2)	28.1 (17.4-42.1)	22.1 (12.6-32.6)	72.2 (36.9-122.7)	85.4 (56.7-179)	26 (17.8-34.5)	25.5 (17.7-38.5)	45.4 (17.2-79.9)	30.5 (15-54.4)	24.6 (14.7-53.7)	15 (14.2-19)	65.4 (44.4-76.9)	60.2 (46.2-112.6)	30.2 (21.2-43.8)	33.1 (23.9-38.8)	69.7 (37.5-131.4)	74.5 (38.6-131)	26.6 (16.6-42.1)	20.6 (14.8-32.6)
**Post Birth**																				
N	59	59	49	49	47	47	48	48	38	38	56	56	19	19	11	11	163	163	164	164
Average (SD)	138 (134.8)	103 (113.2)	39.5 (33.8)	24.7 (20.8)	87.6 (68.7)	103.4 (89.9)	47.6 (60.1)	43.4 (45.6)	137.2 (282.7)	63.1 (163.9)	82 (274.3)	40.7 (109.1)	92.3 (68)	83.4 (99.8)	38.6 (16.3)	238.1 (664.2)	117.9 (164.9)	91.5 (120)	56.3 (164.7)	49.9 (185.6)
Range	12 - 756.5	20.1 - 788.1	11.1 - 171.4	11.6 - 158.1	13.7 - 292.4	12.7 - 423.9	10 - 368.1	13.5 - 235.5	10.5 - 1551.7	10.8 - 910.8	10.7 - 1905.2	10.4 - 647.1	29.7 - 335.2	21.2 - 471.4	19.9 - 64.7	15.5 - 2240.5	10.5 - 1551.7	10.8 - 910.8	10 - 1905.2	10.4 - 2240.5
Median (IQR)	92.1 (64.2-167.5)	69.5 (41-128.3)	28.6 (15.5-44.7)	20.5 (17.2-26.4)	67.9 (42.7-108.7)	90.1 (39.4-127)	28.5 (21.6-51)	27.4 (21.3-43.9)	30.8 (16.9-95.8)	14.9 (12.3-28.9)	15.5 (12.5-36.1)	13.9 (11.8-16.9)	73 (56.5-94.8)	54.9 (41.7-80.1)	37.7 (23.5-51.7)	42.4 (26.9-52)	75.6 (36.4-126.8)	54.9 (28.9-109)	26.1 (14.9-43.2)	20.3 (14.9-30.3)

Note: OAW – non-pregnant other adult women, MOM – pregnant women.

Table S13. Comparison of personal exposures to BC between pregnant and non-pregnant adult women in the same households by arm and study period

BC Exposure ($\mu g/m^3$)	Guatemala				India				Peru				Rwanda				Overall			
	Control		Intervention		Control		Intervention		Control		Intervention		Control		Intervention		Control		Intervention	
	OAW	MOM	OAW	MOM	OAW	MOM	OAW	MOM	OAW	MOM	OAW	MOM	OAW	MOM	OAW	MOM	OAW	MOM	OAW	MOM
**Baseline**																				
N	52	52	64	64	41	41	39	39	40	40	41	41	15	15	8	8	148	148	152	152
Average (SD)	12.7 (5.6)	11.9 (7.8)	13.3 (6.7)	13.2 (9.5)	13.6 (11.3)	13.6 (11)	12.1 (9.5)	14.2 (12.3)	11.7 (14.8)	10.1 (11.6)	13 (12.4)	13.7 (13.5)	12.9 (4.2)	11.7 (4.5)	5.9 (2.6)	12.3 (10.8)	12.7 (10.3)	11.8 (9.6)	12.5 (9.2)	13.6 (11.4)
Range	1.1 - 29	1.7 - 55	4.3 - 46.8	1.5 - 77.2	1.6 - 69.2	1.6 - 54	1.5 - 33.3	1.4 - 48.8	1.4 - 72.3	1.5 - 66.1	1.4 - 49.3	1.4 - 65.7	6.2 - 20.1	4.8 - 20.3	2.8 - 8.9	2.9 - 31.9	1.1 - 72.3	1.5 - 66.1	1.4 - 49.3	1.4 - 77.2
Median (IQR)	11.6 (9.3-15.9)	10.9 (7.8-13.3)	12.4 (9.7-14.9)	11.5 (9.2-14.7)	11.7 (6.2-17.2)	10.5 (8.8-15.8)	8 (4.2-20.6)	7.6 (4.9-24.1)	6 (1.6-14.8)	6.3 (2-13.3)	10.1 (3.4-16.1)	10.6 (2.3-19.7)	11.1 (10.1-15.9)	11.8 (9-14.8)	5.8 (3.8-8.4)	6.8 (4.8-19.2)	11 (6.2-16.1)	10.5 (6.4-14.5)	10.9 (6.7-15.7)	11.2 (6.3-16.6)
**Pregnancy**																				
N	60	60	61	61	44	44	48	48	43	43	49	49	19	19	14	14	166	166	172	172
Average (SD)	11.8 (4.9)	11.3 (4.8)	4.7 (3.3)	4 (3.1)	11.7 (7.8)	13.3 (7.9)	3 (2)	3.2 (2.3)	8.6 (13.3)	8 (12)	2.2 (1.6)	1.8 (0.9)	8.3 (4.6)	11.2 (6.5)	4.2 (1.5)	4 (1.3)	10.5 (8.6)	11 (8.3)	3.5 (2.6)	3.1 (2.5)
Range	1 - 23.7	2.1 - 22.3	0.9 - 16.6	0.9 - 19.1	1.6 - 33.8	2.6 - 35.2	1.4 - 9.1	1.4 - 11.7	1.4 - 83.9	1.3 - 65.3	1.4 - 10.6	1.4 - 7.7	3.4 - 21.5	4.3 - 28.1	2.8 - 6.6	2.5 - 6.3	1 - 83.9	1.3 - 65.3	0.9 - 16.6	0.9 - 19.1
Median (IQR)	11.4 (8.8-14.5)	11.3 (7.4-14.3)	3.5 (2.2-6.3)	3.4 (2.1-5)	11.1 (5.2-16.1)	12.5 (7.3-15.1)	2.2 (1.6-3.9)	2.3 (1.6-3.6)	5.4 (1.5-9.7)	3.9 (1.5-8.6)	1.5 (1.5-2.2)	1.5 (1.5-1.6)	7.6 (5.2-9.5)	9 (6.5-14.9)	3.8 (2.9-5.1)	3.8 (2.9-5)	9.5 (4.8-13.9)	10.2 (5.2-14.2)	2.7 (1.6-4.6)	2.3 (1.5-4.1)
**Post Birth**																				
N	12	12	16	16	47	47	48	48	32	32	50	50	18	18	9	9	109	109	123	123
Average (SD)	14.5 (4.6)	11.2 (4.8)	5 (4.6)	3.7 (3.3)	10.9 (9.5)	12.4 (13.6)	4.2 (7.6)	3.8 (5.8)	6.9 (6.2)	2.7 (1.8)	3.2 (5.2)	1.7 (0.6)	9.3 (3.9)	7.6 (3.3)	4.3 (1.2)	4.2 (1.1)	9.9 (7.7)	8.6 (10.1)	3.9 (6)	3 (4)
Range	7.7 - 23	5 - 20.2	0.9 - 17.4	0.9 - 12.1	1.6 - 49.9	2 - 91.2	1.5 - 53.3	1.6 - 41.1	1.4 - 25.1	1.4 - 7.5	1.4 - 36.1	1.4 - 4	2.8 - 17.2	2.8 - 14.4	2.9 - 6.6	2.8 - 5.7	1.4 - 49.9	1.4 - 91.2	0.9 - 53.3	0.9 - 41.1
Median (IQR)	13.1 (12-16)	10.8 (7.4-13.2)	3.3 (1.7-5.9)	2.2 (1.5-4.5)	8.1 (4.4-14.8)	8.8 (4.9-15.9)	2.1 (1.6-3.6)	2.3 (1.6-4)	4.9 (2.2-9.9)	1.5 (1.5-4.2)	1.5 (1.5-2.5)	1.5 (1.5-1.5)	8.7 (6.7-12.3)	6.9 (5.6-9.6)	4 (3.6-4.9)	4.4 (3-5)	8.1 (4.3-13.6)	5.9 (2.6-11.7)	2 (1.6-3.8)	1.6 (1.5-3)

Note: OAW – non-pregnant other adult women, MOM – pregnant women.

Table S14. Comparison of personal exposures to CO between pregnant and non-pregnant adult women in the same households by arm and study period

CO Exposure (ppm)	Guatemala				India				Peru				Rwanda				Overall			
	Control		Intervention		Control		Intervention		Control		Intervention		Control		Intervention		Control		Intervention	
	OAW	MOM	OAW	MOM	OAW	MOM	OAW	MOM	OAW	MOM	OAW	MOM	OAW	MOM	OAW	MOM	OAW	MOM	OAW	MOM
**Baseline**																				
N	57	57	61	61	40	40	36	36	39	39	37	37	15	15	17	17	151	151	151	151
Average (SD)	1.9 (2)	1.6 (2.1)	1.7 (1.5)	1.6 (1.5)	1.3 (1.4)	1.5 (1.8)	1.9 (3.9)	2.8 (4)	4.1 (4)	2.7 (3.6)	3.9 (6.6)	3.9 (6.7)	1.1 (1)	1 (1.1)	1.5 (2.4)	2.6 (5.4)	2.2 (2.7)	1.8 (2.5)	2.3 (4.1)	2.6 (4.4)
Range	0.1 - 9.5	0 - 12.6	0 - 7.5	0 - 8.3	0 - 6.3	0 - 10	0 - 21.4	0 - 19.9	0 - 18.1	0 - 16.8	0 - 38.7	0 - 32.4	0.1 - 3.8	0 - 3.5	0 - 8.9	0 - 22.5	0 - 18.1	0 - 16.8	0 - 38.7	0 - 32.4
Median (IQR)	1.3 (0.6-2.4)	0.7 (0.4-1.9)	1.4 (0.5-2.6)	1.2 (0.5-2.2)	0.8 (0.3-2.1)	1.1 (0.3-2)	0.8 (0.2-2.1)	1.4 (0.2-3.6)	2.9 (1.5-6.4)	1.4 (0.5-2.8)	2 (1.2-3.3)	1.5 (0.9-3.6)	0.9 (0.4-1.5)	0.6 (0.3-1.1)	0.3 (0.1-1.3)	0.3 (0.2-2.5)	1.4 (0.5-2.9)	0.9 (0.4-2.1)	1.4 (0.4-2.7)	1.3 (0.4-2.8)
**Pregnancy**																				
N	59	59	65	65	45	45	49	49	33	33	46	46	22	22	13	13	159	159	173	173
Average (SD)	2.1 (2.9)	1.7 (1.6)	0.6 (0.8)	0.5 (0.8)	2.4 (3.6)	2.7 (4.2)	0.4 (1.4)	0.2 (0.3)	2.1 (2.4)	2.5 (5.5)	1.8 (2.4)	1 (1.2)	0.8 (0.9)	1.3 (1.3)	1.3 (3.3)	0.8 (1.8)	2 (2.9)	2.1 (3.5)	0.9 (1.9)	0.5 (1)
Range	0 - 20.7	0 - 8	0 - 3.6	0 - 4.5	0 - 17	0 - 24.7	0 - 9.5	0 - 1.4	0 - 10.6	0 - 27.6	0 - 14.1	0 - 5.3	0 - 2.8	0 - 4.9	0 - 12.3	0 - 6.7	0 - 20.7	0 - 27.6	0 - 14.1	0 - 6.7
Median (IQR)	1.5 (0.6-2.6)	1.3 (0.6-2.2)	0.3 (0.1-0.8)	0.2 (0.1-0.5)	1.1 (0.3-2.8)	1.2 (0.4-2.8)	0 (0-0.1)	0 (0-0.3)	1.3 (0.4-3.3)	1 (0.3-1.6)	0.9 (0.3-2.6)	0.5 (0.1-1.4)	0.4 (0.1-1.4)	0.8 (0.3-1.8)	0.3 (0.1-0.7)	0.1 (0.1-0.4)	1.2 (0.4-2.6)	1 (0.4-2.2)	0.2 (0-0.9)	0.2 (0-0.6)
**Post Birth**																				
N	57	57	48	48	47	47	50	50	29	29	37	37	17	17	13	13	150	150	148	148
Average (SD)	1.5 (1.7)	1.2 (1.2)	0.5 (0.5)	0.3 (0.6)	1.6 (1.4)	2.2 (3.3)	0.5 (0.9)	0.3 (0.4)	3.8 (4.4)	2.3 (2.6)	3.5 (4.4)	1.5 (2.3)	1.7 (1.8)	0.8 (0.8)	1.5 (1.8)	0.9 (1.6)	2 (2.6)	1.7 (2.3)	1.3 (2.6)	0.7 (1.4)
Range	0 - 8.6	0 - 5	0 - 2	0 - 3.2	0 - 5.1	0 - 16.7	0 - 4.1	0 - 1.8	0 - 15.8	0.1 - 8.2	0 - 17.8	0 - 12.4	0 - 6.8	0 - 2.5	0 - 5.4	0 - 5.9	0 - 15.8	0 - 16.7	0 - 17.8	0 - 12.4
Median (IQR)	1 (0.4-1.9)	0.8 (0.3-1.8)	0.3 (0.1-0.6)	0.1 (0-0.4)	1.4 (0.4-2.5)	1.1 (0.5-2.7)	0.2 (0-0.7)	0.1 (0-0.4)	2.7 (0.5-5.2)	0.8 (0.4-3.3)	1.7 (0.5-5)	0.7 (0.3-2)	1.2 (0.4-2.4)	0.6 (0.1-1.4)	0.8 (0.2-1.7)	0.3 (0.1-0.9)	1.2 (0.4-2.7)	0.8 (0.3-2.2)	0.3 (0.1-1.3)	0.2 (0-0.7)

Note: OAW – non-pregnant other adult women, MOM – pregnant women.

Table S15. Correlation (Spearman's ρ) of personal measures of exposure between non-pregnant and pregnant adult women by pollutant, stove-type, IRC, and study period

	PM2.5		BC		CO	
**IRC**	**LPG**	**Traditional**	**LPG**	**Traditional**	**LPG**	**Traditional**
**Baseline Period**						
All	---	0.63	---	0.66	---	0.54
Guatemala	---	0.73	---	0.54	---	0.48
India	---	0.71	---	0.72	---	0.56
Peru	---	0.48	---	0.70	---	0.54
Rwanda	---	0.36	---	0.23	---	0.61
**Pregnancy Period**						
All	0.52	0.68	0.74	0.80	0.44	0.51
Guatemala	0.72	0.70	0.70	0.73	0.27	0.69
India	0.55	0.80	0.72	0.87	0.15	0.72
Peru	0.39	0.47	0.30	0.75	0.45	0.13
Rwanda	0.42	0.46	0.68	0.40	0.62	0.23
**Post-birth Period**						
All	0.39	0.53	0.71	0.62	0.35	0.46
Guatemala	0.43	0.68	0.84	0.22	0.19	0.45
India	0.61	0.82	0.84	0.81	0.26	0.48
Peru	0.18	-0.14	0.40	0.09	0.17	0.38
Rwanda	0.72	0.25	0.73	0.38	0.21	0.61

Trial-wide, we observed moderate to strong correlations (Spearman's ρ range: 0.39 – 0.80) for all three pollutants between non-pregnant and pregnant participants living in the same household (**Table S11**). At baseline, the correlations between participant types were 0.63, 0.66, and 0.54 for PM2.5, BC, and CO exposures, respectively. During the pregnancy period, we observed slightly lower, yet still moderate, correlations among participants in LPG-using households compared to those using biomass for PM2.5 (0.52 vs. 0.68), BC (0.74 vs. 0.80), and CO (0.44 vs. 0.51) alike. During the post-birth period, correlations between participant types remained lower among LPG users compared to those using biomass for PM2.5 (0.39 vs. 0.53) and CO (0.35 vs. 0.46); however, the contrary was true for BC (0.71 vs. 0.62).

Table S16. Personal exposures from comparable household air pollution studies

Author	Yr	Study Location	Study Population	Age (years)	Fuel	Descriptive Statistic	PM (μg/m^3)	BC (μg/m^3)	CO (ppm)
Johnson et al.	2022	All study sites	Pregnant Women	18 - 35	LPG	Median	23.7 - 24.1	2.7 - 2.8	0.2
		Guatemala					23.3 - 23.8	2.7 - 2.9	0.1 - 0.2
		India					25.3 - 28.7	2.1 - 2.5	0
		Peru					14.6	1.6	0
		Rwanda					28.2 - 33.6	3.8 - 4.2	0.2
Fandino et al.	2022	Peru	Nonpregnant Women	25 - 64	LPG	Arithmetic Mean	17 - 26	2	3
Fandino et al.	2022	Peru	Nonpregnant Women	25 - 64	Biomass	Arithmetic Mean	104 - 126	2	3
Young et al.	2019	Honduras	Nonpregnant Women	25 - 56	Biomass	Arithmetic Mean	101	17	
Okello et al.	2018	Uganda and Ethiopia	Elderly Women	>50	Biomass	Geometric Mean	45.6 - 63.9		0.2 – 0.5
Thornburg et al.	2022	Bangladesh	Pregnant Women	15 - 37	Biomass	Arithmetic Mean	81.3	56.4	
Thornburg et al.	2022	Bangladesh	Pregnant Women	15 - 37	LPG	Arithmetic Mean	63.1	54	
Raqib et al.	2023	Bangladesh	Adult Women	25 - 65	Biomass	Arithmetic Mean	158.9	7.4	1.0 (μg/m^3)
Raqib et al.	2023	Bangladesh	Adult Women	25 - 65	LPG	Arithmetic Mean	85.6	6.3	0.3 (μg/m^3)
Chillrud et al.	2021	Ghana	Pregnant Women	27 - 28 (Average)	Biomass	Arithmetic Mean	73 - 77		1.1 – 1.3
Chillrud et al.	2021	Ghana	Pregnant Women	27 - 28 (Average)	LPG	Arithmetic Mean	52		0.9
Estevez-Garcia et al.	2020	Mexico	Women	N/A	Biomass	Arithmetic Mean	69.4		
Estevez-Garcia et al.	2020	Mexico	Women	N/A	LPG	Arithmetic Mean	51.9		
Gould et al.	2020	Ecuador	Adult Men and Women	18 - 84	Biomass	Arithmetic Mean	50.8		
Gould et al.	2020	Ecuador	Adult Men and Women	18 - 84	LPG	Arithmetic Mean	22.4		
Pillarisetti et al.	2019	India	Pregnant Women	22 – 24 (Average)	Biomass	Arithmetic Mean	520		
Pillarisetti et al.	2019	India	Pregnant Women	22 – 24 (Average)	LPG	Arithmetic Mean	72		

Weinstein et al.	2020	Guatemala	Pregnant Women	18 - 39	Biomass	Median	102		
Weinstein et al.	2020	Guatemala	Pregnant Women	18 - 39	LPG	Median	45		
Grajeda et al.	2020	Guatemala	Pregnant Women	18 - 40	Biomass	Median	78		
Grajeda et al.	2020	Guatemala	Pregnant Women	18 - 40	LPG	Media	55		
Curto et al.	2019	Mozambique	Childbearing Women	Dec-49	Biomass	Arithmetic Mean		18.6	
Alexander et al.	2018	Nigeria	Pregnant Women	14 - 44	Biomass	Arithmetic Mean	66 - 364		
Alexander et al.	2018	Nigeria	Pregnant Women	14 - 44	Ethanol	Arithmetic Mean	61 - 352		
Shupler et al.	2020	Overall	Adult Women	43 - 84	Biomass	Geometric Mean	71 - 147		
Shupler et al.	2020	Overall	Adult Women	43 - 84	LPG	Geometric Mean	48		
Shupler et al.	2020	India	Adult Women	43 - 84	Biomass	Geometric Mean	89 - 150		
Shupler et al.	2020	India	Adult Women	43 - 84	LPG	Geometric Mean	56		
Shupler et al.	2020	Other South Asia	Adult Women	43 - 84	Biomass	Geometric Mean	147 - 183		
Shupler et al.	2020	South America	Adult Women	43 - 84	Biomass	Geometric Mean	39		
Shupler et al.	2020	South America	Adult Women	43 - 84	LPG	Geometric Mean	23		
Shupler et al.	2020	Africa	Adult Women	43 - 84	Biomass	Geometric Mean	153		
Shupler et al.	2024	Africa	Cooks	35 (SD 11)	LPG	Geometric Mean	43 (23-76)	0·57 (0·12–2·49)	
Shupler et al.	2024	Africa	Children	3.1 (SD 1.3)	LPG	Geometric Mean	47 (18–128)		
Shupler et al.	2024	Africa	Cooks	37 (SD 12)	Wood	Geometric Mean	101 (55–194)	2·52 (0·56–14·09)	
Shupler et al.	2024	Africa	Children	3.1 (SD 1.3)	Wood	Geometric Mean	107 (32–275)		
Benka-Coker et al.	2021	Honduras	Adult Women	24 - 59	Biomass	Median	37 - 47		
Benka-Coker et al.	2021	Honduras	Adult Women	24 - 59	Biomass	Median	76 - 86		
Young et al.	2022	Honduras	Adult Women	24 - 59	Biomass	Median		3.5	
Young et al.	2022	Honduras	Adult Women	24 - 59	Biomass	Median		11.8	

Table S17. Household and participants characteristics at baseline, by IRC and study arm

Variable	Guatemala		India		Peru		Rwanda		Overall	
	Control (n = 67)	Intervention (n = 71)	Control (n = 51)	Intervention (n = 53)	Control (n = 67)	Intervention (n = 66)	Control (n = 24)	Intervention (n = 19)	Control (n = 209)	Intervention (n = 209)
Household and kitchen characteristics										
**Hosuehold Size**										
Mean (SD)	7.8 (3.0)	7.7 (2.8)	4.3 (1.4)	4.4 (1.3)	5.8 (1.9)	5.4 (1.7)	6.1 (2.2)	5.7 (2.2)	6.1 (2.6)	5.9 (2.5)
Range	3-18	3-17	2-8	2-9	3-12	2-10	3-10	2-10	2-18	2-17
**Access to electricity (n (%))**										
No	2 (3%)	5 (7%)	2 (3.9%)	1 (1.9%)	6 (9%)	2 (3%)	16 (66.7%)	12 (63.2%)	26 (12.4%)	20 (9.6%)
Yes	65 (97%)	66 (93%)	49 (96.1%)	52 (98.1%)	61 (91%)	64 (97%)	7 (29.2%)	5 (26.3%)	182 (87.1%)	187 (89.5%)
Missing	0	0	0	0	0	0	1 (4.2%)	2 (10.5%)	1 (0.5%)	2 (1.0%)
**Kitchen volume (m3)**										
Mean (SD)	41.4 (17.7)	38.7 (14.0)	18.3 (13.0)	18.5 (13.8)	20.5 (9.9)	18.1 (10)	12.4 (5.7)	12.4 (4.5)	26.4 (17.6)	25.1 (16.1)
Range	12.2-85.8	10.3-69.9	4.2-64.6	1.8-61.2	4.4-47	4-39.2	4.8-23.5	5.5-23.5	4.2 (85.8)	1.8 (69.9)
n	63	67	51	52	49	51	18	16	180	186
Missing (n)	4	4	0	1	19	15	6	3	29	23
**Roof in the kitchen (n (%))**										
No	66 (98.5%)	70 (98.6%)	0	0	15 (22.4%)	10 (15.2%)	6 (25%)	3 (15.8%)	21 (10.0%)	14 (6.7%)
Yes	0	1 (1.4%)	51 (100%)	53 (100%)	52 (77.6%)	56 (84.8%)	18 (75%)	16 (84.2%)	187 (89.5%)	195 (93.3%)
Missing	1 (1.5%)	0	0	0	0	0	0	0	1 (0.5%)	0
**Number of stoves (n (%))**										
One	12 (17.9%)	14 (19.7%)	29 (56.9%)	32 (60.4%)	19 (28.4%)	18 (27.3%)	14 (58.3%)	10 (52.6%)	74 (35.4%)	74 (35.4%)
Two	43 (64.2%)	40 (56.3%)	21 (41.2%)	20 (37.7%)	41 (61.2%)	44 (66.7%)	8 (33.3%)	8 (42.1%)	113 (54.1%)	112 (53.6%)
Three or more	12 (17.9%)	17 (23.9%)	1 (2%)	1 (1.9%)	7 (10.4%)	4 (6.1%)	1 (4.2%)	1 (5.3%)	21 (10.0%)	23 (11.0%)
Missing	0	0	0	0	0	0	1 (4.2%)	0	1 (0.5%)	0
**Primary stove has a chimney (n (%))**										
No	51 (76.1%)	55 (77.5%)	51 (100%)	52 (98.1%)	38 (56.7%)	41 (62.1%)	22 (91.7%)	19 (100%)	162 (77.5%)	167 (79.9%)
Yes	16 (23.9%)	16 (22.5%)	0	1 (1.9%)	29 (43.3%)	25 (37.9%)	1 (4.2%)	0	46 (22.0%)	42 (20.1%)
Missing	0	0	0	0	0	0	1 (4.2%)	0	1 (0.5%)	0
**Primary Cook (n (%))**										
Pregnant women	37 (55.2%)	39 (54.9%)	46 (90.2%)	48 (90.6%)	10 (14.9%)	13 (19.7%)	17 (73.9%)	15 (78.9%)	110 (52.9%)	115 (55.0%)
Other adult women	29 (43.3%)	32 (45.1%)	4 (7.8%)	5 (9.4%)	56 (83.6%)	53 (80.3%)	3 (13.0%)	1 (5.3%)	92 (44.2%)	91 (43.5%)
Other/Missing	1 (1.5%)	0	1 (2.0%)	0	1 (1.5%)	0	3 (13.0%)	3 (15.8%)	6 (2.9%)	3 (1.4%)

**Primary fuel type (n (%))**										
Cow dung	0	0	0	0	62 (93.9%)	54 (81.8%)	0	0	63 (30.1%)	54 (25.8%)
Wood	67 (100%)	70 (100%)	51 (100%)	53 (100%)	4 (6.1%)	8 (12.1%)	23 (95.8%)	16 (84.2%)	145 (69.4%)	148 (70.8%)
Charcoal	0	0	0	0	0	0	0	3 (15.8%)	0	3 (1.4%)
Other	0	0	0	0	0	4 (6.1%)	0	0	0	4 (1.9%)
Missing	0	0	0	0	0	0	1 (4.2%)	0	1 (0.5%)	0
**Primary stove location (n (%))**										
In participant's bedroom	1 (1.5%)	1 (1.4%)	7 (10%)	11 (20.8%)	0	0	0	1 (5.3%)	8 (3.8%)	13 (6.2%)
Room immediately adjacent to the participant's bedroom	21 (31.3%)	18 (25.4%)	22 (43.1%)	13 (24.5%)	1 (1.5%)	4 (6.1%)	0	0	44 (21.1%)	35 (16.7%)
Separated from the participant’s bed-room but inside the house	27 (40.3%)	32 (45.1%)	9 (17.6%)	11 (20.8%)	9 (13.4%)	13 (19.7%)	0	2 (10.5%)	45 (21.5%)	58 (27.8%)
Outside the house (outdoors)	1 (1.5%)	1 (1.4%)	0	0	18 (26.9%)	10 (15.2%)	6 (25%)	3 (15.8%)	25 (12.0%)	14 (6.7%)
In a separate building detached from the bedroom-main home	17 (25.4%)	19 (26.8%)	13 (25.5%)	18 (34%)	39 (58.2%)	39 (59.1%)	17 (70.8%)	13 (68.4%)	86 (41.1%)	89 (42.6%)
Missing	0	0	0	0	0	0	1 (4.2%)	0	1 (0.5%)	0
**Primary light source (n (%))**										
Torch (battery)	0	0	0	1 (1.9%)	0	0	4 (16.7%)	5 (26.3%)	4 (1.9%)	6 (2.9%)
Kerosene lamp	0	0	3 (5.9%)	3 (5.7%)	0	0	1 (4.2%)	1 (5.3%)	4 (1.9%)	4 (1.9%)
Solar light	0	0	0	0	3 (4.5.%)	3 (4.5%)	10 (41.7%)	7 (36.8%)	13 (6.2%)	10 (4.8%)
Electricity	65 (97%)	65 (91.5%)	48 (94.1%)	49 (92.5%)	60 (89.6%)	60 (90.9%)	6 (25%)	4 (21.1%)	179 (85.6%)	178 (85.2%)
Other	2 (3%)	6 (8.5%)	0	0	4 (6.0%)	3 (4.5%)	2 (8.3%)	2 (10.5%)	8 (3.8%)	11 (5.3%)
Missing	0	0	0	0	0	0	1 (4.2%)	0	1 (0.5%)	0
**Presence of a smoker in home (n (%))**										
No	58 (86.6%)	64 (90.1%)	32 (62.7%)	35 (66%)	67 (100%)	66 (100%)	22 (91.7%)	17 (89.5%)	179 (85.6%)	182 (87.1)
Yes	9 (13.4%)	7 (9.9%)	19 (37.3%)	18 (34%)	0	0	1 (4.2%)	2 (10.5%)	29 (13.9%)	27 (12.9%)
Missing	0	0	0	0	0	0	1 (4.2%)	0	1 (0.5%)	0
Participant characteristics										
**Age (year)**										
Mean (SD)	53.3 (6.7)	53.8 (9.1)	49.5 (5.7)	48.6 (7.2)	52.3 (8.7)	52.8 (6.9)	51.0 (8.3)	55.2 (8.9)	51.8 (7.5)	52.3 (8.2)
Range	41.5-73.8	40.4-74.2	40.6-68	40.2-71.6	40.1-73.5	40.9-73.6	40.5-66.1	42.6-74.3	40.1-73.8	40.2-74.3
**Occupation (n (%))**										

Agriculture	0	1 (1.4%)	41 (80.4%)	38 (71.7%)	9 (13.4%)	14 (21.2%)	21 (87.5%)	12 (63.2%)	71 (34.0%)	65 (31.1%)
Commercial	1 (1.5%)	3 (4.2%)	1 (2%)	2 (3.8%)	2 (3.0%)	2 (3.0%)	1 (4.2%)	3 (15.8%)	5 (2.4%)	10 (4.8%)
Household	65 (97%)	66 (93%)	4 (7.8%)	7 (13.2)	53 (79.1%)	46 (69.7%)	0	1 (5.3%)	122 (58.4%)	120 (57.4%)
Other	1 (1.5%)	1 (1.4%)	2 (3.9%)	2 (3.8%)	3 (4.5.%)	3 (4.5%)	2 (8.3%)	1 (5.3%)	8 (3.8%)	7 (3.3%)
Unemployed	0	0	3 (5.9%)	4 (7.5%)	0	1 (1.5%)	0	2 (10.5%)	3 (1.4%)	7 (3.3%)
**Education (n (%))**										
No formal education or primary school incomplete	62 (92.5%)	64 (90.1%)	49 (96.1%)	50 (94.3%)	42 (62.7%)	38 (57.6%)	14 (58.3%)	16 (84.2%)	167 (79.9%)	168 (80.4%)
Primary school or secondary school incomplete	1 (1.5%)	3 (4.2%)	2 (3.9%)	3 (5.7%)	23 (34.3%)	26 (39.4%)	5 (20.8%)	2 (10.5%)	31 (14.8%)	34 (16.3%)
Secondary school or vocational or some college/university	0	1 (1.4%)	0	0	2 (3.0%)	1 (1.5%)	5 (20.8%)	1 (5.3%)	7 (3.3%)	3 (1.4%)
Missing	4 (6.0%)	3 (4.2%)	0	0	0	1 (1.5%)	0	0	4 (1.9%)	4 (1.9%)

Table S18. PM2.5 exposures by specific household / participants characteristics for each country, study arm, and study period

PM2.5 Exposure		Guatemala		India		Peru		Rwanda		Overall	
		Control	Intervention	Control	Intervention	Control	Intervention	Control	Intervention	Control	Intervention
Baseline											
Cooking Activity											
	Older Adult Woman	108.5 (69.3-145) [63]	115.3 (77.7-180.8) [65]	72.4 (41.9-119.9) [42]	71.6 (39.1-134.9) [44]	44.3 (16-123) [37]	55.5 (24.6-122.4) [37]	103.9 (62.3-158.9) [20]	43.6 (27.8-60.2) [15]	90 (41.9-134.6) [162]	82.2 (40.7-149.1) [161]
	Other	95.6 (95.6-95.6) [1]	127.7 (66.5-127.8) [5]	89.1 (66.7-127) [5]	54.9 (47.6-62.3) [2]	86.8 (46-146.7) [20]	65.7 (43.9-148.1) [19]	57.5 (52.2-114.9) [3]	95.5 (85.2-130.9) [3]	89.1 (50.4-137.3) [29]	69.6 (45-127.8) [29]
	Missing					12.5 (12.5-12.5) [1]				12.5 (12.5-12.5) [1]	
Kitchen Location											
	In participant's bedroom	120.9 (120.9-120.9) [1]	127.8 (127.8-127.8) [1]	120.6 (75.9-169.9) [7]	75.7 (65.2-89.6) [9]	116.6 (116.6-116.6) [1]		54.1 (54.1-54.1) [1]		118.6 (70.5-154.7) [10]	79.7 (66.9-118.2) [10]
	Room immediately adjacent to the participant's bedroom	105.7 (79-121.8) [19]	123 (77.7-189.2) [17]	69.6 (44.1-93.1) [19]	89 (44.3-135) [12]	51.3 (23.9-98.9) [4]	55.5 (33.7-75.3) [7]			88.4 (45.7-115.7) [42]	92.4 (52.8-175.9) [36]
	Separated from the participant's bedroom but inside the house	105.8 (77.1-154.1) [29]	104.3 (71.4-153.7) [34]	56.5 (33.3-118.3) [8]	53.9 (40.7-70.8) [9]	33.8 (16-123.4) [12]	42.1 (22.4-118.6) [12]	118 (118-118) [1]	36.9 (36.9-36.9) [1]	96.2 (40.3-130.3) [50]	83.8 (43.8-138.5) [56]
	In a separate building detached from the bedroom (main house)	112.3 (36.4-158) [15]	133.4 (94-184.9) [16]	89.1 (52.1-127) [13]	60.9 (25.8-197.1) [16]	86.7 (32.5-136.6) [27]	73.7 (46.5-178.1) [28]	101.8 (61-170.3) [15]	40.7 (24.1-67.1) [12]	89.2 (45-143) [70]	77.5 (35.2-168) [72]
	Outside the house (outdoors)		264.2 (155.3-373.1) [2]			54.8 (28.6-85) [12]	34.5 (19.6-58.8) [9]	100.8 (62.1-144.7) [6]	74.9 (60.2-79.4) [5]	66.6 (30.1-103.4) [18]	53.6 (32.1-83.7) [16]
	Other										
	Missing					220 (116.2-323.7) [2]				220 (116.2-323.7) [2]	
Ventilation											
	Window/opening above stove	110.9 (76.4-149.5) [58]	115.3 (77.8-182) [63]	63.2 (34-110.9) [9]	62.4 (41.4-128.1) [14]	67.9 (32.4-123.6) [26]	62 (32.7-84.1) [30]	101.8 (61-151) [11]	51.9 (40.4-67.1) [8]	97.6 (56.4-136.3) [104]	87.4 (52-162.4) [115]
	None	45.6 (38.2-95.8) [5]	115.9 (44-127.8) [5]	80.9 (44.9-126.9) [38]	73.4 (32.4-117.4) [32]	91.1 (22.5-149.2) [19]	109.1 (37.2-298.5) [18]	86.8 (54.5-162.7) [6]	36.9 (23.5-49) [7]	86.6 (37.6-133.1) [68]	72.9 (28.6-138.7) [62]
	Missing	93.6 (93.6-93.6) [1]	264.2 (155.3-373.1) [2]			38.5 (25.7-84.2) [13]	29.6 (22.3-46.9) [8]	100.8 (89.4-144.7) [6]	79.4 (77.1-102.2) [3]	79 (31.3-108.6) [20]	46.3 (24.6-79.4) [13]
Pregnancy											

Cooking Activity											
	Older adult woman	90.5 (53.9-173.9) [104]	25.5 (11.9-40.1) [114]	68.7 (37.2-122.8) [76]	18.1 (17.8-35.5) [74]	39.9 (15-91.8) [54]	17 (14.7-45) [78]	64.1 (36-77.4) [25]	26.6 (20.3-41.5) [19]	69.7 (36.7-138.7) [259]	23.7 (15.2-39) [285]
	Other person	71.2 (51.5-102) [4]	14.5 (13.2-15.9) [2]	32.3 (29.9-36.7) [5]	19.4 (17-21.8) [4]	37.9 (19.8-63.9) [26]	21 (15.1-60.3) [13]	81.2 (55.2-134.5) [11]	26.6 (21-34.7) [4]	44.6 (29.7-78.1) [46]	21 (15.1-37.3) [23]
	Missing		22.4 (19-26.5) [4]			14.9 (14.9-15) [2]		34.5 (34.5-34.5) [1]	47.9 (47.9-47.9) [1]	15 (14.9-24.7) [3]	23.5 (21.4-35.7) [5]
Kitchen Location											
	In participant's bedroom	160.9 (160.9-160.9) [1]	72 (56.4-87.6) [2]	85.5 (43.6-104.2) [11]	17.9 (17.6-35.7) [19]				41.1 (41.1-41.1) [1]	85.5 (47.1-105.5) [12]	18 (17.7-39.7) [22]
	Room immediately adjacent to the participant's bedroom	84.4 (49.4-179.6) [40]	23.4 (11.7-40.5) [37]	67.4 (38.6-120.8) [38]	26.1 (17.9-38.1) [39]	93.1 (64.8-171.5) [5]	15.5 (14.7-20.9) [4]		37.1 (20.3-42.9) [9]	76.5 (45.3-166.7) [83]	24.5 (17.6-40.5) [89]
	Separated from the participant's bedroom but inside the house	93.1 (54.1-149.5) [45]	26.9 (11.9-38.3) [44]	68.3 (29.9-143.2) [12]	17.8 (13.6-24.7) [11]	91.6 (36.1-153.8) [12]	15.2 (14.5-41.2) [20]		27.9 (21-38.2) [8]	87.5 (47.4-149.5) [69]	24.5 (14-38.5) [83]
	In a separate building detached from the bedroom (main house)	85.2 (58.8-180.5) [21]	24.2 (14.9-32.4) [32]	60.6 (30.8-147.7) [16]	17.8 (17.8-19.8) [7]	26.1 (14.9-61.4) [42]	22.1 (14.9-54.2) [65]	66.4 (42.7-89.3) [24]	20.7 (20.2-26.6) [5]	55.9 (24.6-97.1) [103]	22.1 (15-42.4) [109]
	Outside the house (outdoors)	257.5 (257.5-257.5) [1]		19 (16.3-36.2) [3]		41.6 (15.7-64.5) [17]		63 (53.4-84.3) [12]		56.6 (29.9-67.5) [33]	
	Other					15 (15-15) [1]	35.7 (35.7-35.7) [1]			15 (15-15) [1]	35.7 (35.7-35.7) [1]
	Missing		21.4 (17.2-23.5) [5]	29.9 (29.9-29.9) [1]	19.4 (18.6-20.2) [2]	15 (14.8-19.8) [5]	15.2 (15.2-15.2) [1]	34.5 (34.5-34.5) [1]	47.9 (47.9-47.9) [1]	19.8 (14.9-30.1) [7]	21 (17.2-23.5) [9]
Ventilation											
	Window/opening above stove	38.2 (27.9-48.5) [2]	28.4 (18.1-30.7) [10]	85.5 (85.5-85.5) [1]	26.7 (21-35) [7]	27.2 (20.7-33.7) [2]	112.6 (16.9-228) [4]		20.7 (20.3-38.9) [4]	40.2 (17.6-58.8) [5]	26.7 (17.8-35.6) [25]
	None		26.5 (26.5-26.5) [1]	67.4 (67.4-67.4) [1]	17.9 (17.6-45.1) [18]	21.4 (21.4-21.4) [1]	31.5 (14.8-61.5) [19]		36.2 (23.8-40.1) [6]	44.4 (32.9-55.9) [2]	23 (16-50.7) [44]
	Missing	90.5 (54.4-172.1) [106]	24.5 (11.8-38.7) [109]	61.3 (32.8-122.1) [79]	18 (17.8-32.2) [53]	38.4 (15-82.1) [79]	16 (14.7-40.4) [68]	65 (43.7-87.3) [37]	29.3 (21-42.5) [14]	65.4 (34.5-124.1) [301]	22.6 (15-38.3) [244]
Post-birth											
Cooking Activity											
	Older Adult Woman	88.7 (45.5-154.6) [126]	26.6 (13-41.6) [119]	67.9 (32.2-106.5) [101]	21.6 (16.2-40.3) [114]	29.2 (14.8-91.7) [59]	14.7 (12.6-26.7) [89]	72.7 (58.9-116.4) [34]	39 (33.1-56.6) [17]	68.7 (32.1-130.8) [320]	21.7 (13.7-39) [339]
	Other	61.8 (42.2-85.4) [3]	29.8 (12.6-32.4) [5]	36.4 (20.8-125.5) [6]	30 (30-30) [1]	19.8 (15.1-35.6) [11]	15.7 (13.1-27.9) [16]	92.2 (89.7-105.4) [3]	42.2 (26.2-50.6) [5]	33.5 (16.1-96.2) [23]	21.2 (13.6-34.6) [27]

	Missing	66 (66-66) [1]	37.8 (37.8-37.8) [1]					47.5 (40.6-104.4) [7]	64.3 (64.3-64.3) [1]	56.8 (41.2-101.8) [8]	51 (44.4-57.7) [2]
Kitchen Location											
	In participant's bedroom	134.9 (132.8-136.9) [2]	37.9 (33.1-56.9) [4]	48.1 (25.7-97.2) [23]	17.9 (13.6-40.5) [49]	32.9 (16.9-252.8) [8]	13.8 (12.4-15.3) [2]			48.1 (22.9-126.7) [33]	18 (13.6-40.8) [55]
	Room immediately adjacent to the participant's bedroom	74.8 (41.2-136.5) [49]	26.6 (12.5-38.3) [44]	74 (38.2-117) [52]	27.4 (17.8-39.2) [46]	79.1 (79.1-79.1) [1]	14.4 (12.8-18.4) [10]		39 (25.9-55.5) [5]	75.7 (38.4-128.3) [102]	26.2 (15.8-38.6) [105]
	Separated from the participant's bedroom but inside the house	92.1 (50.7-164.2) [55]	25.4 (12.6-34.9) [41]	77.8 (51.3-94.5) [15]	27 (14.5-45.3) [11]	20.1 (13.4-74.7) [22]	14.4 (12.5-21.1) [35]		42.4 (33.4-58) [10]	68.4 (28.5-122.8) [92]	21.2 (12.9-34.9) [97]
	In a separate building detached from the bedroom (main house)	108.9 (41.3-200.7) [23]	27.5 (16.8-45.6) [35]	51.7 (17.6-93.2) [16]	29.1 (18.1-31.3) [9]	34 (15.3-70.1) [28]	15 (12.7-31.7) [54]	71.9 (57.4-89.8) [24]	42.2 (30.2-50.1) [7]	62.6 (27.5-103.6) [91]	21 (14.1-38.4) [105]
	Outside the house (outdoors)					27.8 (12.6-63.1) [11]		118.5 (67.6-158.3) [13]		66.6 (25.8-147.4) [24]	
	Other						81.3 (81.3-81.3) [1]				81.3 (81.3-81.3) [1]
	Missing	66 (66-66) [1]	37.8 (37.8-37.8) [1]	1447.6 (1447.6-1447.6) [1]			25.2 (18.4-30.6) [3]	47.5 (40.6-104.4) [7]	64.3 (64.3-64.3) [1]	66 (41.8-109.6) [9]	35.9 (25.2-37.8) [5]
Ventilation											
	Window/opening above stove	110 (43.8-141.3) [8]	52.2 (24-104.4) [5]	38.3 (38.3-38.3) [1]		37.3 (25-67.8) [3]	109.3 (109.3-109.3) [1]			71.6 (38.1-124.8) [12]	78.3 (31-108.1) [6]
	None	250.6 (250.6-250.6) [1]	171.4 (171.4-171.4) [1]	25.2 (17.6-40.3) [5]	24.9 (15.7-54) [11]	29.2 (22.9-214) [7]	19.5 (14.1-25.2) [9]		52.1 (52.1-52.1) [1]	29.2 (19.9-105.6) [13]	22.5 (14.2-49.4) [22]
	Missing	82.5 (45.5-147.1) [121]	26.6 (12.7-38) [119]	71.1 (32.3-111.8) [101]	21.6 (16.2-39.1) [104]	25.4 (14.8-80.6) [60]	14.7 (12.6-26.9) [95]	74.6 (51.8-111.8) [44]	40.6 (28-57.2) [22]	68 (31.5-126.1) [326]	21.3 (13.6-38.2) [340]

Table S19. CO exposures by specific household / participants characteristics for each country, study arm, and study period

CO Exposure		Guatemala		India		Peru		Rwanda		Overall	
		Control	Intervention	Control	Intervention	Control	Intervention	Control	Intervention	Control	Intervention
Baseline											
Cooking Activity											
	Older Adult Woman	1.3 (0.6-2.4) [57]	1.4 (0.5-2.6) [61]	0.7 (0.2-1.7) [40]	0.6 (0.2-2) [42]	2.6 (1.6-5.3) [27]	1.9 (0.7-5) [34]	0.8 (0.4-1.8) [18]	0.3 (0.1-1.3) [14]	1.2 (0.5-2.4) [142]	1.3 (0.3-2.6) [151]
	Other	2.9 (2.9-2.9) [1]	1.4 (0.6-1.7) [5]	0.5 (0.3-2.4) [5]	4 (4-4) [1]	3.3 (1.5-8) [17]	2 (1.6-3.4) [14]	0.5 (0.4-0.9) [3]	0.4 (0.4-1.6) [3]	2.6 (0.6-3.8) [26]	1.8 (1.2-3.1) [23]
	Missing					0.2 (0.2-0.2) [1]				0.2 (0.2-0.2) [1]	
Kitchen Location											
	In participant's bedroom	3.8 (3.8-3.8) [1]	1.4 (1.4-1.4) [1]	1 (0.7-1.8) [8]	0.8 (0.3-1.8) [8]	1.8 (1.8-1.8) [1]		1.9 (1.9-1.9) [1]		1.4 (0.8-2.4) [11]	0.9 (0.4-1.7) [9]
	Room immediately adjacent to the participant's bedroom	1 (0.3-1.7) [15]	1.5 (0.2-2.8) [16]	0.4 (0.2-1.8) [20]	0.8 (0.3-2.1) [13]	1.5 (0.7-4.2) [3]	1.8 (1.6-3.3) [5]			0.6 (0.2-1.7) [38]	1.5 (0.4-2.2) [34]
	Separated from the participant's bedroom but inside the house	1.3 (0.6-2.4) [26]	1.4 (0.6-2.5) [31]	0.4 (0.1-0.9) [6]	0.5 (0.1-1.6) [8]	3.6 (2.6-5.1) [11]	1.5 (0.6-3.2) [12]		3.8 (3.8-3.8) [1]	1.7 (0.6-3.3) [43]	1.4 (0.5-2.5) [52]
	In a separate building detached from the bedroom (main house)	1.3 (0.7-2.5) [16]	1.1 (0.5-2.2) [16]	1 (0.4-2.7) [11]	0.6 (0.2-3.7) [14]	4.7 (2.4-6.9) [19]	2.5 (1.2-4.7) [23]	0.8 (0.5-1.8) [14]	0.3 (0.1-0.4) [11]	1.7 (0.7-3.5) [60]	1.3 (0.3-3) [64]
	Outside the house (outdoors)		2.3 (1.2-3.5) [2]			0.8 (0.5-2) [9]	1.8 (1-3.1) [8]	0.5 (0.1-1.1) [6]	1.3 (0.4-5.4) [5]	0.8 (0.5-1.6) [15]	1.6 (0.4-5) [15]
	Other										
	Missing					6.2 (3.2-9.2) [2]				6.2 (3.2-9.2) [2]	
Ventilation											
	Window/opening above stove	1.3 (0.6-2.4) [52]	1.4 (0.5-2.5) [59]	0.7 (0.4-1.8) [11]	1.4 (0.2-3.6) [14]	3.8 (2.1-6.1) [20]	1.5 (0.7-3.2) [26]	1 (0.6-2.2) [10]	0.3 (0.1-0.3) [7]	1.4 (0.6-3.2) [93]	1.4 (0.5-2.6) [106]
	None	1 (0.3-2.9) [5]	1.4 (0.3-1.7) [5]	0.6 (0.2-1.7) [34]	0.5 (0.2-1.7) [29]	4 (1.9-7.5) [14]	2.7 (1.7-6.8) [15]	0.3 (0.1-0.7) [5]	0.5 (0.1-3.3) [7]	1 (0.3-2.7) [58]	1.2 (0.2-2.9) [56]
	Missing	0.6 (0.6-0.6) [1]	2.3 (1.2-3.5) [2]			1.8 (0.5-2.1) [11]	1.8 (1.4-2) [7]	1 (0.4-1.3) [6]	1.3 (0.8-3.4) [3]	1 (0.5-1.9) [18]	1.7 (1-2.7) [12]
Pregnancy											

Cooking Activity											
	Older adult woman	1.4 (0.7-2.6) [101]	0.3 (0-0.8) [104]	1.2 (0.3-2.5) [73]	0 (0-0.1) [77]	1.2 (0.3-2.7) [45]	0.7 (0.2-2.8) [73]	0.3 (0.1-1.5) [24]	0.1 (0-0.4) [15]	1.2 (0.4-2.6) [243]	0.2 (0-0.8) [269]
	Other person	1.4 (0.8-1.6) [5]	1.5 (1.5-1.5) [1]	0 (0-0) [4]	0.1 (0.1-0.9) [5]	0.9 (0.2-2.8) [20]	0.5 (0.2-1.2) [6]	0.5 (0.3-1.6) [10]	1 (0.5-1) [3]	0.6 (0.2-1.7) [39]	0.7 (0.1-1.2) [15]
	Missing		0 (0-0.2) [4]			6.1 (6.1-6.1) [1]		0.2 (0.2-0.2) [1]	0.5 (0.5-0.5) [1]	3.2 (1.7-4.6) [2]	0 (0-0.5) [5]
Kitchen Location											
	In participant's bedroom	2.8 (2.8-2.8) [1]	0.3 (0.3-0.3) [1]	1.4 (0.9-2.1) [8]	0 (0-0) [21]				0 (0-0) [1]	1.5 (0.9-2.7) [9]	0 (0-0) [23]
	Room immediately adjacent to the participant's bedroom	0.9 (0.6-2.4) [40]	0.2 (0-0.4) [32]	0.8 (0.3-2.1) [41]	0 (0-0.1) [39]	1.6 (0.2-3.1) [4]	1.4 (1.2-3) [4]	4.9 (4.9-4.9) [1]	0.2 (0.1-0.4) [7]	0.9 (0.3-2.4) [86]	0.1 (0-0.3) [82]
	Separated from the participant's bedroom but inside the house	1.6 (0.7-2.8) [43]	0.5 (0-1.3) [39]	1.1 (0.6-2.3) [11]	0 (0-0.2) [11]	3.8 (1-6.7) [9]	0.2 (0.1-0.9) [18]		1 (0.1-1.1) [5]	1.6 (0.7-3) [63]	0.3 (0-1.1) [73]
	In a separate building detached from the bedroom (main house)	1.3 (0.8-2.3) [21]	0.2 (0-0.7) [32]	1.5 (0-4.5) [15]	0 (0-0.1) [8]	1.2 (0.2-2.5) [36]	0.7 (0.3-2.9) [56]	0.3 (0.1-1.3) [21]	0.1 (0-1) [5]	1 (0.1-2.5) [93]	0.4 (0.1-1.4) [101]
	Outside the house (outdoors)	2.1 (2.1-2.1) [1]		0.4 (0.4-0.4) [1]		1.2 (0.5-1.8) [13]		0.5 (0.1-1.6) [12]		0.7 (0.3-1.7) [27]	
	Other					0.2 (0.2-0.2) [1]	4.4 (4.4-4.4) [1]			0.2 (0.2-0.2) [1]	4.4 (4.4-4.4) [1]
	Missing		0 (0-0.5) [5]	0 (0-0) [1]	0.1 (0-0.5) [3]	1.1 (0.7-3.6) [3]		0.2 (0.2-0.2) [1]	0.5 (0.5-0.5) [1]	0.3 (0.2-1.1) [5]	0.1 (0-0.5) [9]
Ventilation											
	Window/opening above stove	0.8 (0.3-1.7) [4]	0.1 (0-0.7) [22]	0.2 (0.1-0.3) [2]	0.3 (0-0.6) [9]	2.7 (2.7-2.7) [1]	0 (0-0.1) [7]	1.3 (1.3-1.3) [1]	1.2 (0.6-2) [4]		0.2 (0.1-0.3) [2]
	None	0.2 (0.1-0.3) [2]	0.3 (0-0.6) [9]	2.7 (2.7-2.7) [1]	0 (0-0.1) [7]	1.3 (1.3-1.3) [1]	1.2 (0.6-2) [4]		0.2 (0.1-0.3) [2]	0.8 (0.3-1.7) [4]	0.1 (0-0.7) [22]
	Missing	1.4 (0.7-2.6) [104]	0.3 (0-0.9) [99]	1 (0.1-2.3) [75]	0 (0-0.1) [57]	1.2 (0.3-3) [64]	0.5 (0.2-2.7) [59]	0.4 (0.1-1.6) [35]	0.1 (0-1) [11]	1.2 (0.3-2.5) [278]	0.2 (0-0.9) [226]
Post-birth											
Cooking Activity											
	Older Adult Woman	1 (0.3-2.4) [116]	0.1 (0-0.5) [114]	0.7 (0.2-2.1) [104]	0.1 (0-0.3) [117]	1.6 (0.3-5) [41]	1 (0.4-5) [67]	0.8 (0.3-2.5) [33]	0.8 (0.1-1.5) [16]	0.9 (0.2-2.5) [294]	0.2 (0-0.8) [314]
	Other	0.1 (0.1-0.3) [3]	0 (0-0) [3]	0.1 (0-1.4) [5]	0 (0-0) [1]	2.5 (1.5-5.8) [7]	1.4 (0.8-4.4) [11]	0.3 (0.2-0.5) [4]	0.6 (0.1-3) [5]	0.6 (0.1-2.4) [19]	0.8 (0-2.8) [20]
	Missing	0.5 (0.5-0.5) [1]	0 (0-0) [1]					0.2 (0.1-0.5) [6]	0.6 (0.4-0.8) [2]	0.3 (0.1-0.6) [7]	0.2 (0.1-0.6) [3]

Kitchen Location											
	In participant's bedroom	0.8 (0.8-0.8) [1]	0.1 (0.1-0.2) [3]	0.7 (0.1-2.2) [22]	0.1 (0-0.4) [48]	2 (0.4-6.6) [4]	0.6 (0.6-0.6) [1]			0.8 (0.2-2.3) [27]	0.1 (0-0.4) [52]
	Room immediately adjacent to the participant's bedroom	0.8 (0.2-2.2) [46]	0.3 (0-0.5) [43]	0.8 (0.2-2.1) [55]	0 (0-0.2) [49]		1 (0.6-4.6) [8]		0.5 (0.1-0.9) [4]	0.8 (0.2-2.2) [101]	0.1 (0-0.5) [104]
	Separated from the participant's bedroom but inside the house	1.1 (0.3-2.9) [51]	0.2 (0.1-0.5) [37]	1.1 (0.4-2) [15]	0.1 (0-0.7) [11]	0.9 (0.1-2.5) [17]	2.4 (0.5-5.3) [26]		1.3 (0.2-1.9) [11]	1.1 (0.3-2.7) [83]	0.4 (0.1-2) [85]
	In a separate building detached from the bedroom (main house)	1 (0.5-1.9) [21]	0.1 (0-0.4) [34]	0.2 (0.1-1.3) [16]	0 (0-0.2) [10]	1.1 (0.5-5.3) [19]	1.7 (0.3-4.7) [41]	0.4 (0.1-1.1) [26]	0.6 (0.2-1.3) [6]	0.6 (0.2-1.9) [82]	0.3 (0-1.8) [91]
	Outside the house (outdoors)					4.7 (2.7-6.8) [8]		2.1 (1-2.7) [11]		2.7 (1.9-4.4) [19]	
	Other						0.7 (0.7-0.7) [1]				0.7 (0.7-0.7) [1]
	Missing	0.5 (0.5-0.5) [1]	0 (0-0) [1]	0.1 (0.1-0.1) [1]			0 (0-0) [1]	0.2 (0.1-0.5) [6]	0.6 (0.4-0.8) [2]	0.2 (0.1-0.5) [8]	0.1 (0-0.4) [4]
Ventilation											
	Window/opening above stove	1.3 (0.3-2.1) [8]	0.8 (0.1-1.6) [4]	0.3 (0.3-0.3) [1]	0.1 (0.1-0.1) [1]	0.9 (0.9-0.9) [1]	0 (0-0) [1]		0 (0-0) [1]	0.7 (0.3-2.1) [10]	0.1 (0-0.8) [7]
	None	2 (2-2) [1]	3.3 (3.3-3.3) [1]	0.8 (0.2-1.6) [4]	0.1 (0.1-2.2) [9]	10 (5.8-12.4) [3]	3.9 (1-5.4) [6]		5.4 (5.4-5.4) [1]	1.8 (1-4.4) [8]	2.2 (0.1-4.4) [17]
	Missing	0.9 (0.3-2.4) [111]	0.1 (0-0.5) [113]	0.7 (0.1-2.1) [104]	0 (0-0.3) [108]	2.2 (0.4-5.1) [44]	1.2 (0.4-4.8) [71]	0.5 (0.2-1.9) [43]	0.6 (0.2-1.4) [21]	0.8 (0.2-2.5) [302]	0.2 (0-0.8) [313]

Table S20. BC exposures by specific household / participants characteristics for each country, study arm, and study period

BC Exposure		Guatemala		India		Peru		Rwanda		Overall	
		Control	Intervention	Control	Intervention	Control	Intervention	Control	Intervention	Control	Intervention
Baseline											
Cooking Activity											
	Older Adult Woman	11.4 (8.7-15.1) [53]	12.6 (9.8-15.6) [62]	11.7 (7.2-17.2) [41]	7.9 (3.7-21.2) [42]	4.2 (1.6-16.4) [30]	11.2 (3.1-16.7) [30]	12.1 (10.4-15.9) [16]	5.8 (4.1-8.5) [10]	10.9 (6.7-16.1) [140]	10.9 (6.8-16.5) [144]
	Other	17.8 (17.8-17.8) [1]	11.1 (9.5-12.1) [4]	9.1 (6.1-24.2) [5]	9.6 (8-11.2) [2]	9.6 (5.8-15.2) [17]	9.4 (4.5-15.9) [17]	6.8 (5.2-8.5) [2]		9.6 (5.8-16.1) [25]	11 (5.1-14.2) [23]
	Missing					1.5 (1.5-1.5) [1]				1.5 (1.5-1.5) [1]	
Kitchen Location											
	In participant's bedroom	8.3 (8.3-8.3) [1]	11 (11-11) [1]	17.2 (15.4-21.2) [7]	16.6 (10.2-22) [8]	15.2 (15.2-15.2) [1]		7.3 (7.3-7.3) [1]		15.6 (9.9-19.2) [10]	15.5 (11-21.9) [9]
	Room immediately adjacent to the participant's bedroom	10.3 (9.5-13.9) [16]	12.7 (10.4-15.1) [16]	11.7 (9-15.5) [19]	10 (5-21) [11]	4.8 (2.1-9.8) [4]	11.8 (4.7-16.2) [7]			10.5 (8.2-15.1) [39]	12.1 (7.2-16) [34]
	Separated from the participant's bedroom but inside the house	12.4 (10.2-16.2) [24]	12.4 (10.1-14.6) [32]	7.2 (6.1-9.7) [7]	6.4 (3.6-8) [9]	1.6 (1.5-10.8) [9]	6.7 (2.8-13.9) [9]	11.1 (11.1-11.1) [1]	2.8 (2.8-2.8) [1]	11.1 (7.2-15.9) [41]	10.9 (6.8-13.9) [51]
	In a separate building detached from the bedroom (main house)	10.3 (7.7-14.3) [13]	14.1 (8.7-16.7) [15]	11.3 (5.3-18.3) [13]	7.6 (3.3-23.9) [16]	10.2 (4.2-17.2) [22]	14.9 (8.9-22.2) [24]	12.6 (10.5-16.2) [12]	5.8 (4.1-8.4) [8]	10.9 (5.9-17.1) [60]	10.7 (6.6-17.3) [63]
	Outside the house (outdoors)		11.1 (10.3-11.8) [2]			3.7 (2.5-10.7) [10]	4.2 (1.5-6.8) [7]	11.3 (8.7-13.8) [4]	8.9 (8.9-8.9) [1]	6 (3-12.9) [14]	6.8 (2.3-9) [10]
	Other										
	Missing					36.9 (19.2-54.6) [2]				36.9 (19.2-54.6) [2]	
Ventilation											
	Window/opening above stove	11.5 (9.3-15.1) [49]	12.6 (10.1-15.5) [59]	11.7 (6.2-16.8) [9]	7.5 (6.4-9) [13]	6 (2.4-10.8) [22]	11.8 (5.5-16.1) [25]	11 (10.6-16.1) [9]	8.3 (4.7-8.5) [5]	10.7 (6.3-15.1) [89]	11.6 (7.9-15.3) [102]
	None	8.2 (7.6-10.6) [4]	11 (7.1-13) [5]	11.3 (7.2-18.4) [37]	12.8 (3.4-21.6) [31]	14.2 (1.6-20.7) [16]	15.8 (6-24.1) [16]	11.1 (7.3-15.7) [5]	3.5 (2.9-4.8) [4]	11.2 (6.5-18.3) [62]	11 (3.4-21.7) [56]
	Missing	15.9 (15.9-15.9) [1]	11.1 (10.3-11.8) [2]			5 (2.6-14.5) [10]	2.9 (1.5-4.8) [6]	11.5 (9.8-13.8) [4]	8.9 (8.9-8.9) [1]	9.9 (3.7-15.5) [15]	5 (1.6-9) [9]

Pregnancy											
Cooking Activity											
	Older adult woman	11.1 (8.4-14.5) [97]	3.4 (2.1-6) [110]	8.7 (4.6-17.2) [73]	1.6 (1.6-3.4) [73]	3.4 (1.5-11) [47]	1.5 (1.5-2.2) [75]	7.6 (5.1-8.5) [23]	4.4 (2.9-5.1) [17]	9.6 (5-14) [240]	2.4 (1.5-4.4) [275]
	Other person	9.3 (5.6-12.5) [4]	2.5 (2.5-2.5) [2]	3.8 (3.2-5.2) [5]	3.1 (2.1-4.1) [4]	1.5 (1.4-6.9) [19]	1.5 (1.5-1.5) [9]	8.8 (7.2-12.6) [9]	2.9 (2.9-3) [4]	5.4 (1.5-9.4) [37]	1.6 (1.5-2.9) [19]
	Missing		2.7 (1.5-4) [4]			1.5 (1.5-1.5) [1]		5.1 (5.1-5.1) [1]	6.6 (6.6-6.6) [1]	3.3 (2.4-4.2) [2]	3.9 (1.6-4.2) [5]
Kitchen Location											
	In participant's bedroom	23.6 (23.6-23.6) [1]	7.9 (6.7-9.1) [2]	13.9 (7.8-18.8) [11]	1.6 (1.6-2.5) [19]				4.9 (4.9-4.9) [1]	14.2 (8.7-21.7) [12]	1.6 (1.6-4.6) [22]
	Room immediately adjacent to the participant's bedroom	10.8 (8.4-13.3) [37]	2.9 (2-4.7) [34]	8.7 (5.5-20.1) [37]	2.8 (1.6-3.9) [38]	5.4 (1.9-10.8) [5]	1.5 (1.5-1.5) [4]		4.1 (2.8-5.6) [8]	10.4 (6.9-14.5) [79]	2.8 (1.6-4.3) [84]
	Separated from the participant's bedroom but inside the house	11.1 (8-12.7) [41]	3.2 (2.4-6) [43]	9.8 (3.2-13.9) [11]	1.6 (1.6-2.3) [11]	10.6 (5.9-15) [10]	1.5 (1.5-2.1) [18]		3 (2.9-3.5) [8]	10.5 (6.8-15) [62]	2.7 (1.6-4.8) [80]
	In a separate building detached from the bedroom (main house)	12.1 (8.5-16.4) [21]	3.4 (2-5.9) [32]	5.2 (3.2-11.3) [15]	1.6 (1.6-2.2) [7]	2.3 (1.5-9.2) [34]	1.5 (1.5-1.6) [60]	7.6 (5.5-8.8) [21]	3.6 (2.9-4.5) [4]	7.1 (3-12) [91]	1.6 (1.5-3.1) [103]
	Outside the house (outdoors)	11.9 (11.9-11.9) [1]		4.1 (3.8-7.5) [3]		1.5 (1.5-5.9) [13]		7.7 (5.5-10.7) [11]		5.5 (1.5-9.4) [28]	
	Other					1.5 (1.5-1.5) [1]	11.9 (11.9-11.9) [1]			1.5 (1.5-1.5) [1]	11.9 (11.9-11.9) [1]
	Missing		2.5 (1.6-3.9) [5]	3.8 (3.8-3.8) [1]	3.3 (2.8-3.9) [2]	1.5 (1.4-1.5) [4]	1.5 (1.5-1.5) [1]	5.1 (5.1-5.1) [1]	6.6 (6.6-6.6) [1]	1.5 (1.5-3.2) [6]	2.5 (1.6-4.2) [9]
Ventilation											
	Window/opening above stove	5.4 (5-5.9) [2]	2.9 (1.9-4) [10]	14.5 (14.5-14.5) [1]	3.5 (1.7-3.7) [7]	6.6 (4-9.2) [2]	2.7 (2.1-6.6) [3]		2.9 (2.8-3.8) [4]	6.4 (4.5-11.8) [5]	2.9 (1.8-4) [24]
	None		2.7 (2.7-2.7) [1]	20.7 (20.7-20.7) [1]	1.6 (1.6-2.9) [18]	1.4 (1.4-1.4) [1]	1.5 (1.5-2.4) [17]		4.6 (4-5.1) [4]	11.1 (6.2-15.9) [2]	1.6 (1.5-2.9) [40]
	Missing	11.2 (8.4-14) [99]	3.4 (2.1-6.3) [105]	8.3 (4.1-16.8) [76]	1.6 (1.6-3.4) [52]	2.3 (1.5-10) [64]	1.5 (1.5-1.5) [64]	7.6 (5.4-8.8) [33]	3 (2.9-5.1) [14]	8.7 (4.4-13.6) [272]	2.4 (1.5-4.4) [235]
Post-birth											
Cooking Activity											
	Older Adult Woman	11.7 (7.4-15.2) [98]	3.4 (2.2-8.4) [103]	8.2 (4.6-14.3) [93]	1.6 (1.6-3.5) [108]	2.9 (1.5-9.7) [51]	1.5 (1.5-2.4) [80]	8.9 (5.1-12.4) [33]	4 (3-4.7) [14]	8.9 (4.6-14.1) [275]	2.3 (1.6-4.5) [305]

	Other	9.8 (7.8-11.5) [3]	3.3 (2.7-4.1) [4]	3.3 (1.8-17.5) [6]	1.6 (1.6-1.6) [1]	2.6 (1.8-6.9) [10]	1.5 (1.5-2.4) [15]	11.1 (10-11.7) [3]	5.1 (4.6-5.3) [5]	5.2 (2.4-12) [22]	2.4 (1.5-4.6) [25]
	Missing	10.4 (10.4-10.4) [1]	14.7 (14.7-14.7) [1]					9.8 (4.7-14.1) [6]	7.8 (7.8-7.8) [1]	10.4 (5-14) [7]	11.2 (9.5-13) [2]
Kitchen Location											
	In participant's bedroom	12 (11.4-12.7) [2]	6.6 (4.4-10.5) [4]	7 (3.7-10.4) [21]	1.6 (1.6-2.9) [48]	3 (1.7-7.5) [8]	1.5 (1.5-1.5) [1]			7 (3-10.6) [31]	1.6 (1.6-3.5) [53]
	Room immediately adjacent to the participant's bedroom	11.2 (7.3-15.4) [35]	3.2 (2.2-7.2) [39]	8.4 (5.7-15.2) [49]	1.7 (1.6-3.5) [41]	11.7 (11.7-11.7) [1]	1.5 (1.5-2.4) [9]		4.4 (3.9-5.5) [4]	10.1 (6.3-15.2) [85]	3 (1.6-4.4) [93]
	Separated from the participant's bedroom but inside the house	11.4 (7.4-15.1) [44]	3.5 (2.8-6.5) [33]	8.7 (6.8-18.5) [13]	1.9 (1.6-4.3) [11]	1.6 (1.5-9.3) [19]	1.5 (1.5-2.3) [33]		4.5 (3-5.3) [9]	9.1 (4.8-14.1) [76]	2.8 (1.5-4.6) [86]
	In a separate building detached from the bedroom (main house)	12.1 (8-13.9) [20]	4.3 (1.9-9.6) [31]	4.4 (2.5-10) [15]	1.6 (1.6-1.7) [9]	2.4 (1.5-9.1) [25]	1.5 (1.5-2.5) [49]	8.1 (4.8-11.4) [23]	3.9 (2.9-5) [6]	7.9 (3-11.8) [83]	2.2 (1.5-4.8) [95]
	Outside the house (outdoors)					6.2 (2.5-11.6) [8]		10.7 (7.4-16.9) [13]		9.4 (5.1-15.2) [21]	
	Other						9.4 (9.4-9.4) [1]				9.4 (9.4-9.4) [1]
	Missing	10.4 (10.4-10.4) [1]	14.7 (14.7-14.7) [1]	31.2 (31.2-31.2) [1]			1.5 (1.5-1.5) [2]	9.8 (4.7-14.1) [6]	7.8 (7.8-7.8) [1]	12.1 (5.4-15.1) [8]	4.7 (1.5-9.5) [4]
Ventilation											
	Window/opening above stove	15.4 (13.4-17.8) [6]	7.8 (4.2-13.8) [5]	2.6 (2.6-2.6) [1]		3.8 (2.7-7.6) [3]	20.4 (20.4-20.4) [1]			12.1 (5.1-15.5) [10]	10.8 (5.1-13.9) [6]
	None	12.5 (12.5-12.5) [1]	19.6 (19.6-19.6) [1]	4.3 (4-5.4) [4]	2.6 (1.6-7.6) [11]	3.7 (1.5-7.9) [5]	1.5 (1.5-1.9) [8]			4.3 (3.3-8.2) [10]	1.6 (1.5-6.6) [20]
	Missing	11 (7.3-14.7) [95]	3.4 (2.2-7.3) [102]	8.2 (4.6-15.2) [94]	1.6 (1.6-3.4) [98]	2.4 (1.5-9.4) [53]	1.5 (1.5-2.4) [86]	8.9 (5.3-12.4) [42]	4.4 (3-5.4) [20]	8.8 (4.5-13.9) [284]	2.4 (1.6-4.5) [306]

FIGURES

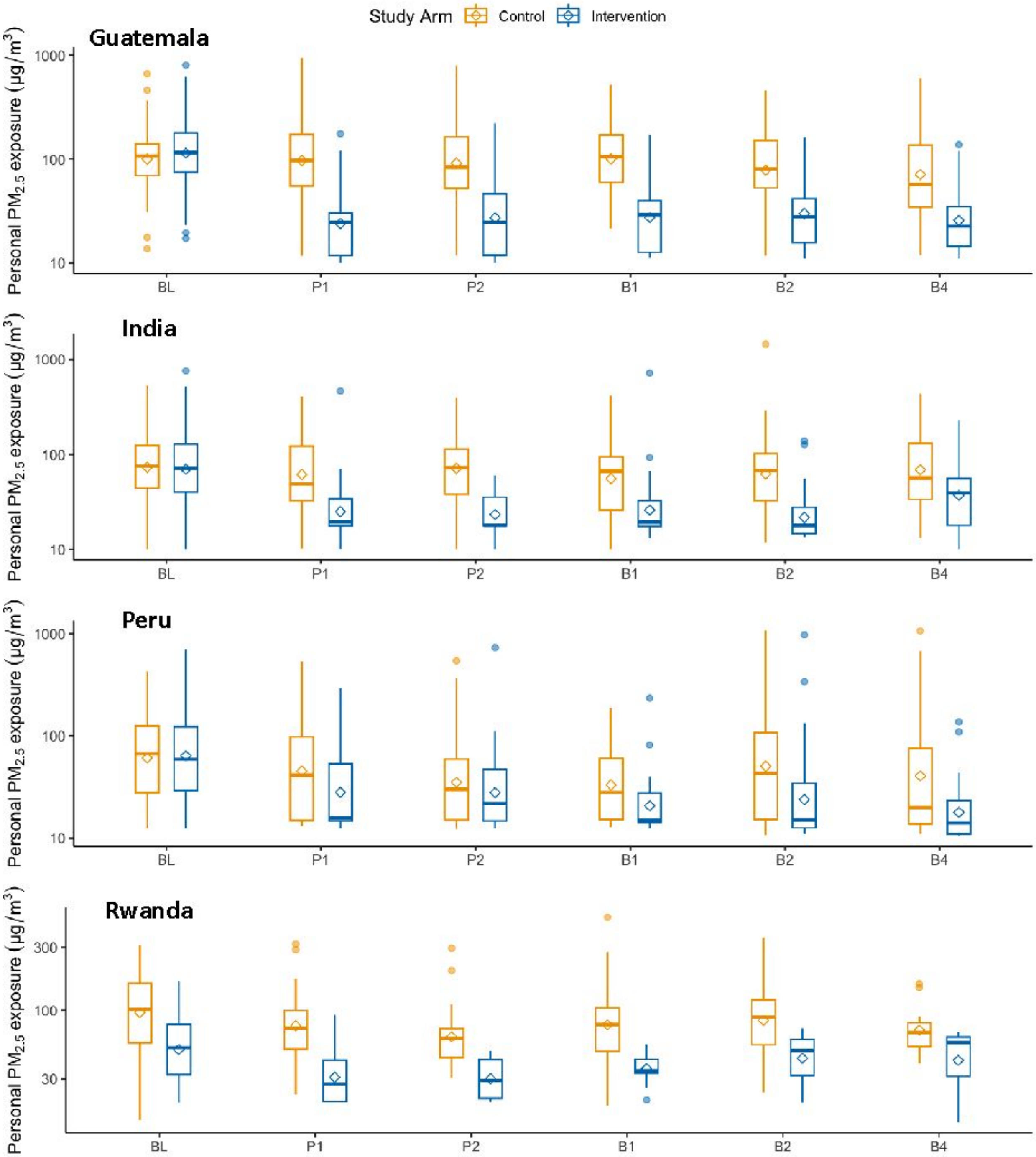

Figure S1. Boxplot of personal exposure to $PM_{2.5}$ among non-pregnant adult women participants by IRC, study group, and visit.

The square in each box indicates the mean value. The highest 2.5% of the datapoints are not shown in the figure.

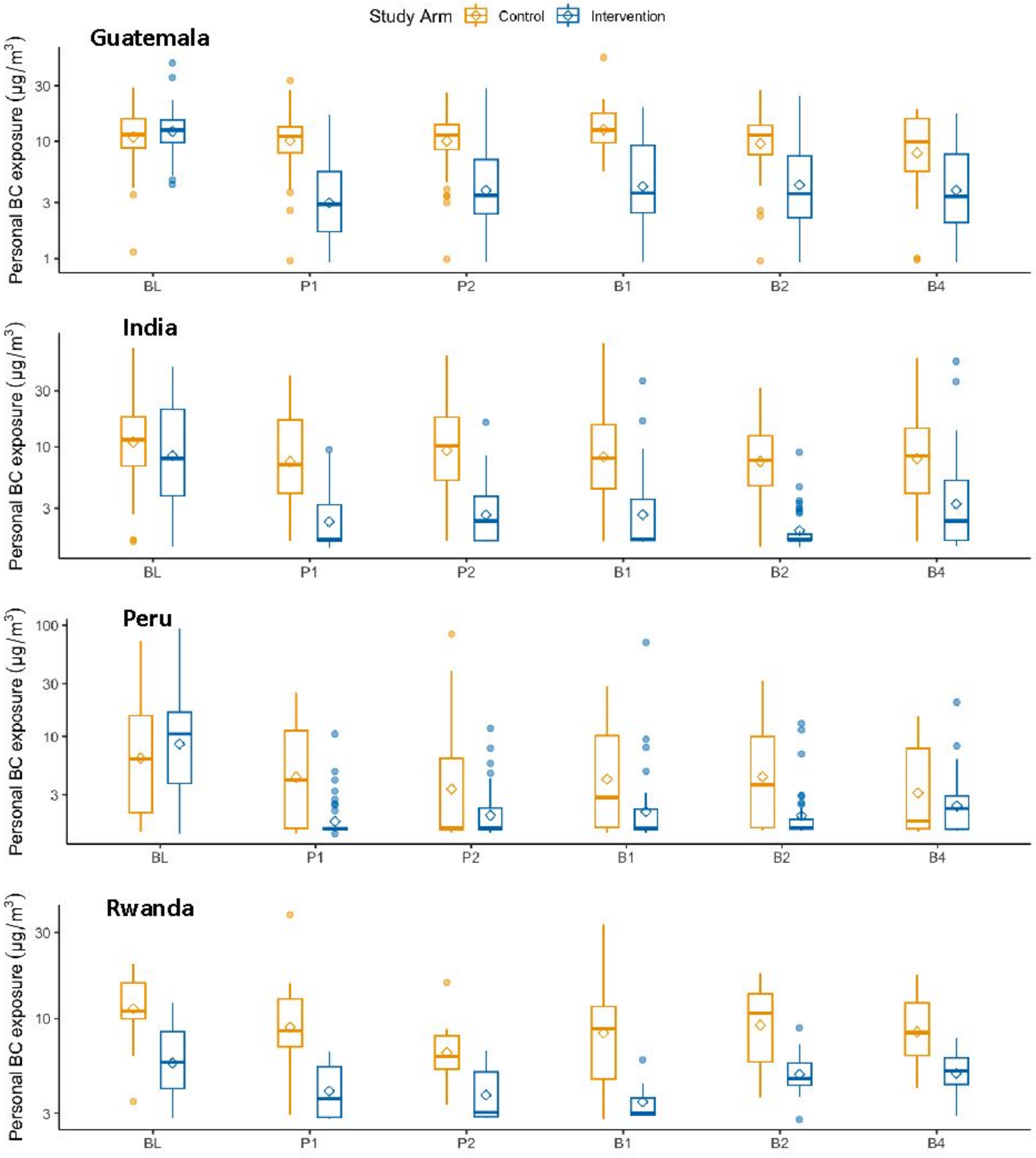

Figure S2. Boxplot of personal exposure to BC among non-pregnant adult women participants by IRC, study group, and visit.

The square in each box indicates the mean value. The highest 2.5% of the datapoints are not shown in the figure.

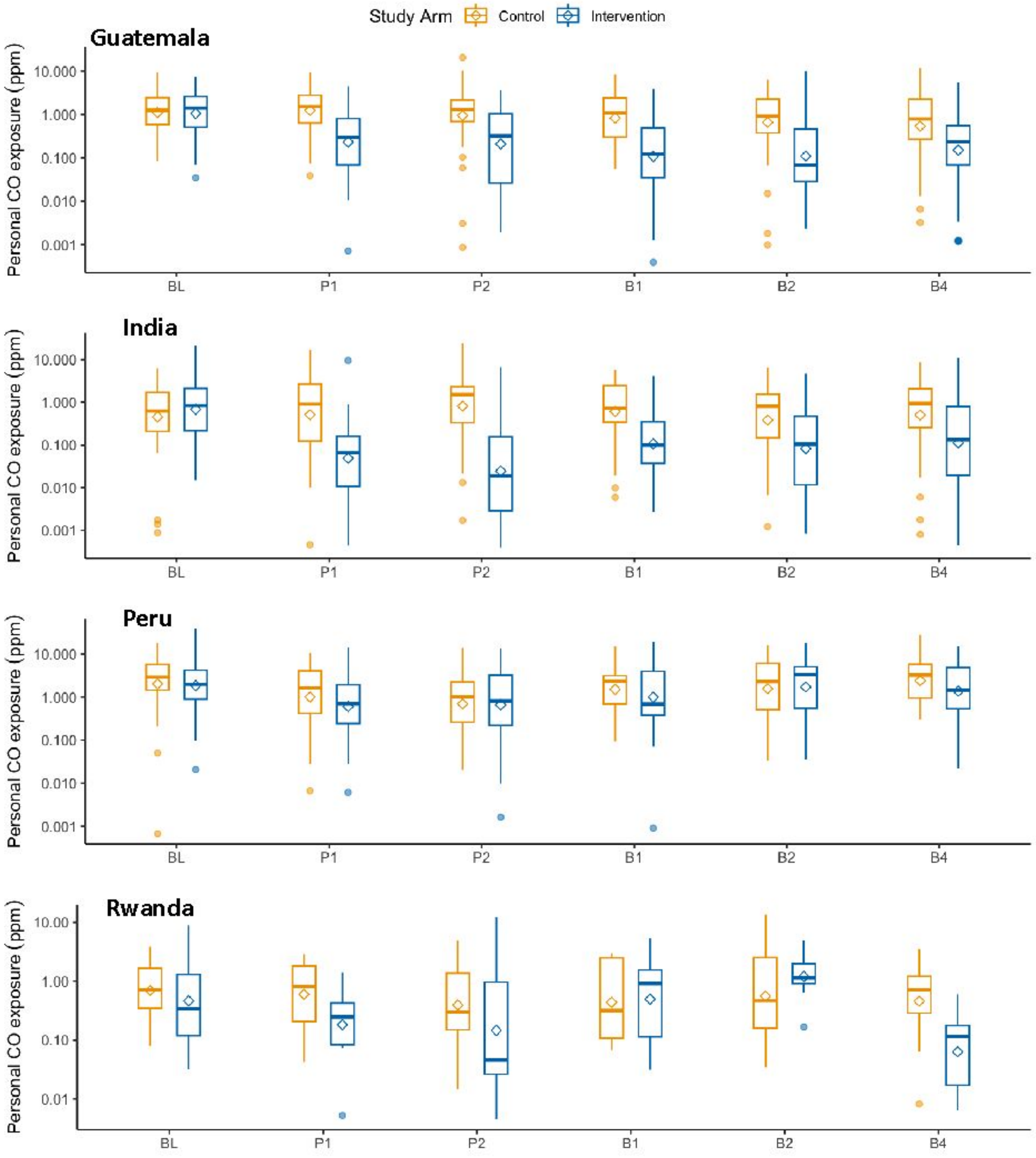

Figure S3. Boxplot of personal exposure to CO among non-pregnant adult women participants by IRC, study group, and visit.

The square in each box indicates the mean value. The highest 2.5% of the datapoints are not shown in the figure.

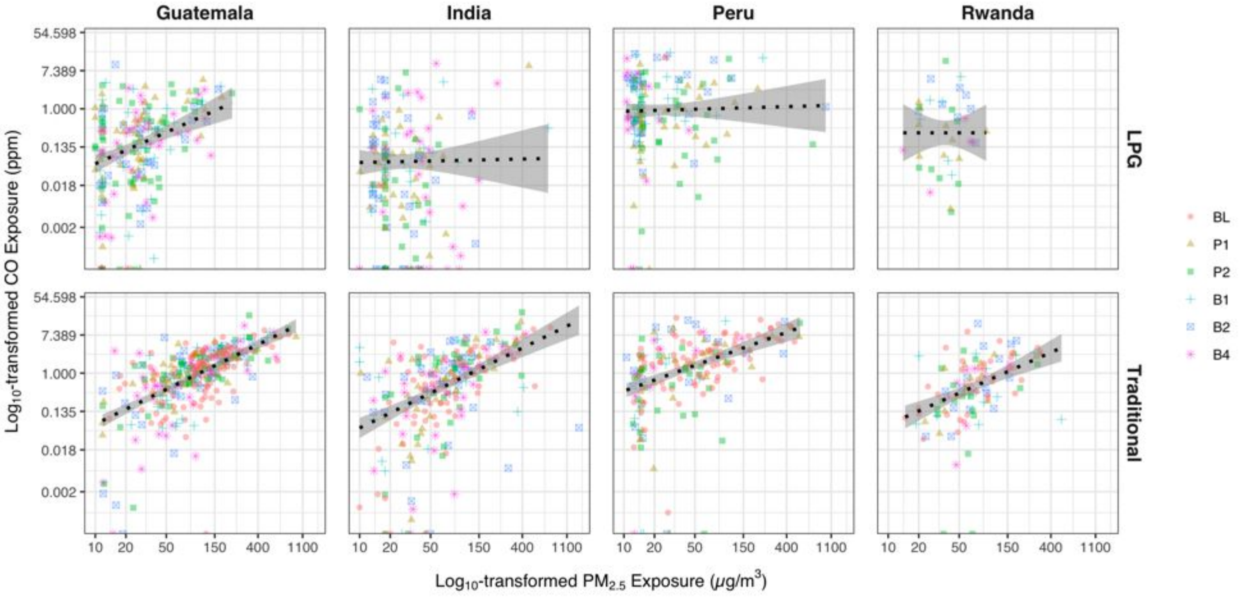

Figure S4. Correlation between $\log_{10}$-transformed $PM_{2.5}$ and CO exposure by stove type and IRC

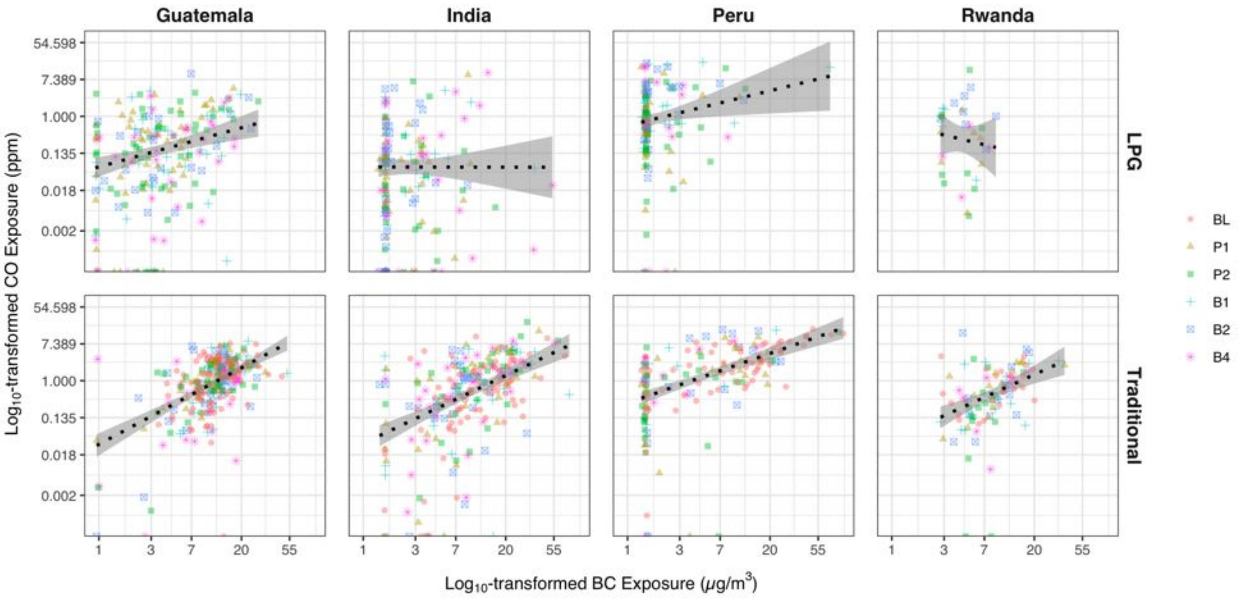

Figure S5. Correlation between $\log_{10}$-transformed BC and CO exposure by stove type and IRC

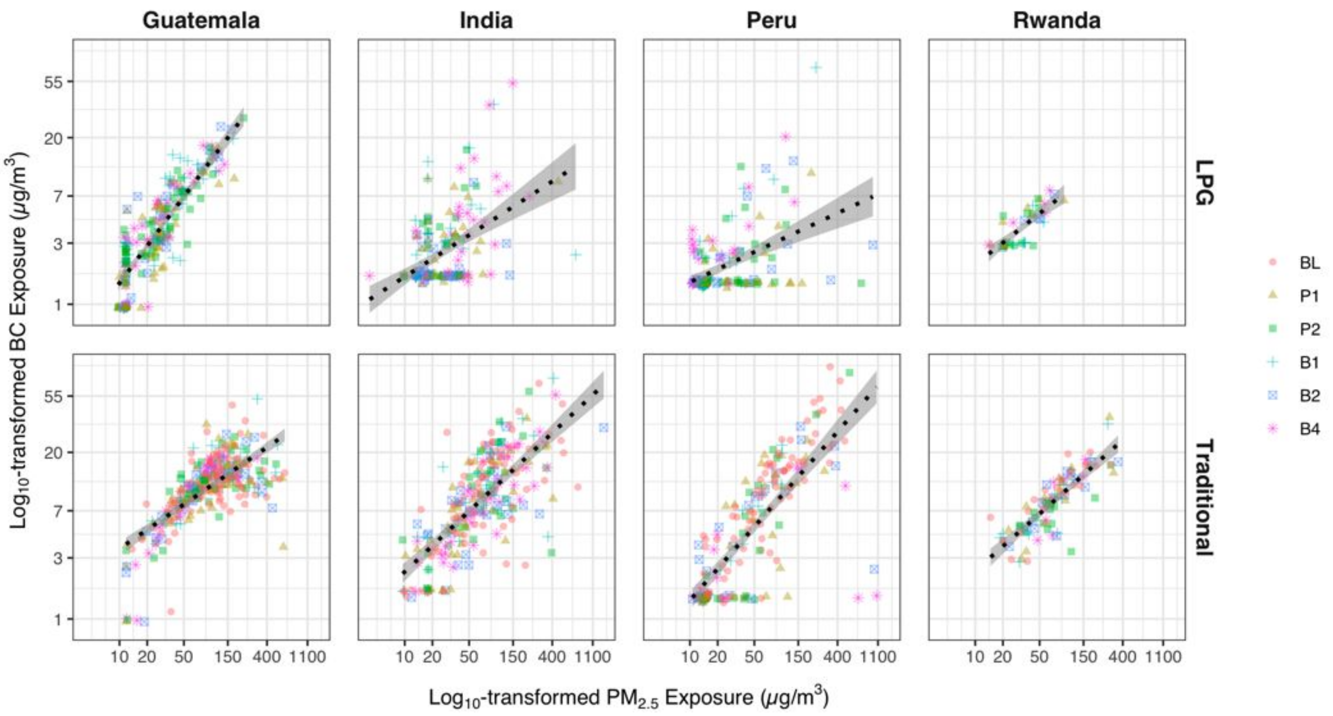

Figure S6. Correlation between $\log_{10}$-transformed $PM_{2.5}$ and BC exposure by stove type and IRC

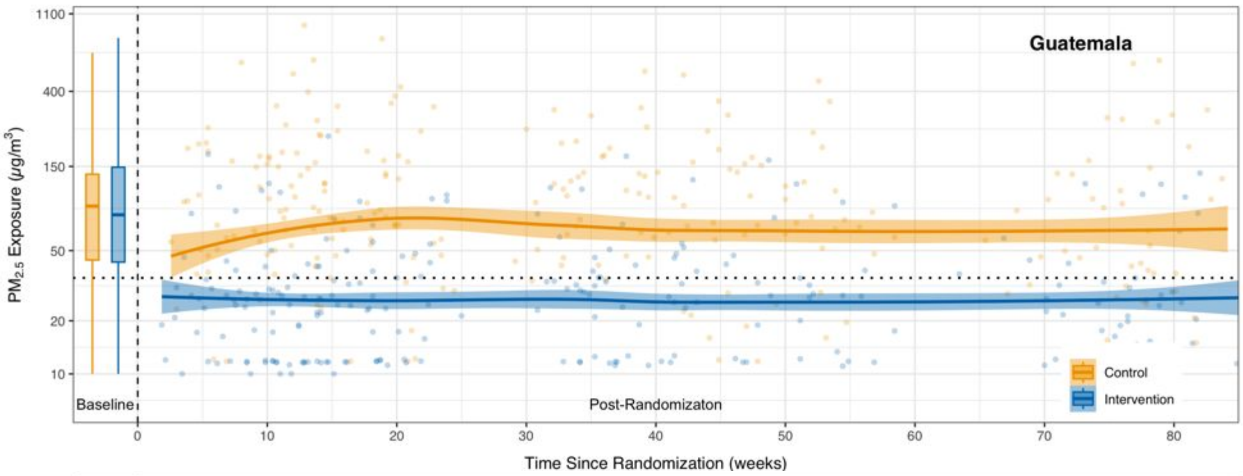
Guatemala
PM2.5 Exposure (µg/m3)
1100
400
150
50
20
10
Baseline
Post-Randomizaton
Control
Intervention
0
10
20
30
40
50
60
70
80
Time Since Randomization (weeks)

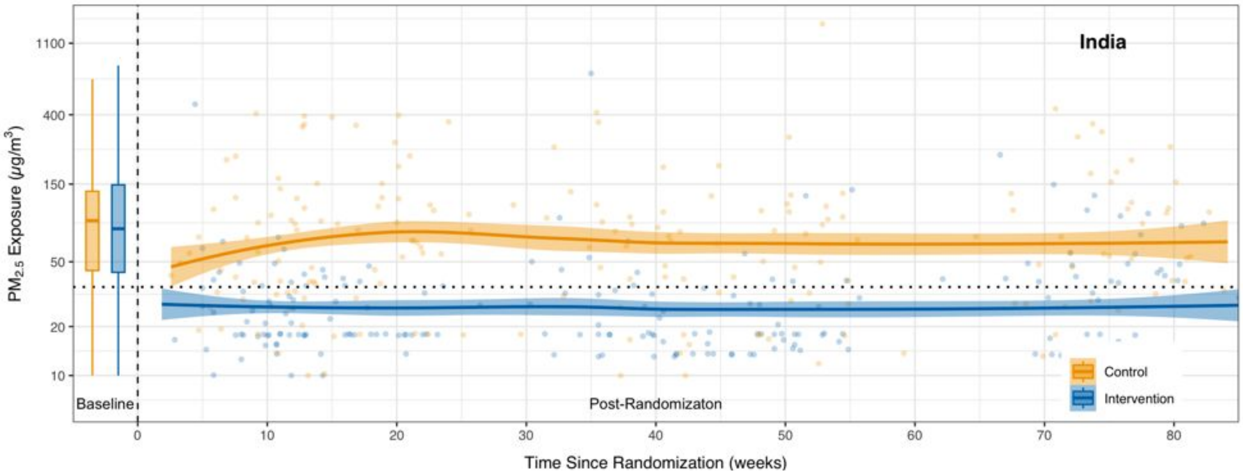
India
PM2.5 Exposure (µg/m3)
1100
400
150
50
20
10
Baseline
Post-Randomizaton
Control
Intervention
0
10
20
30
40
50
60
70
80
Time Since Randomization (weeks)

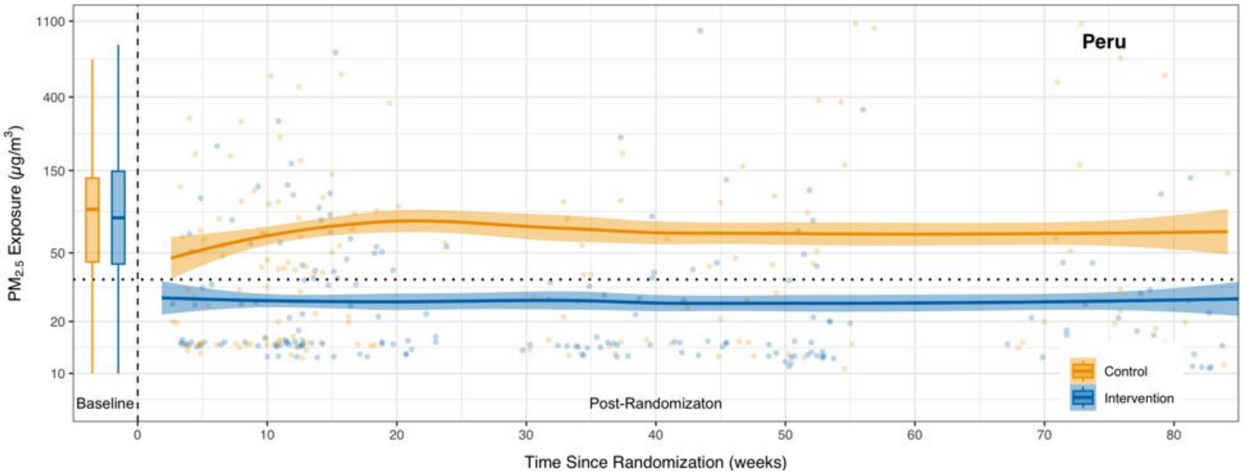
Peru
PM2.5 Exposure (µg/m3)
1100
400
150
50
20
10
Baseline
Post-Randomizaton
Control
Intervention
0
10
20
30
40
50
60
70
80
Time Since Randomization (weeks)

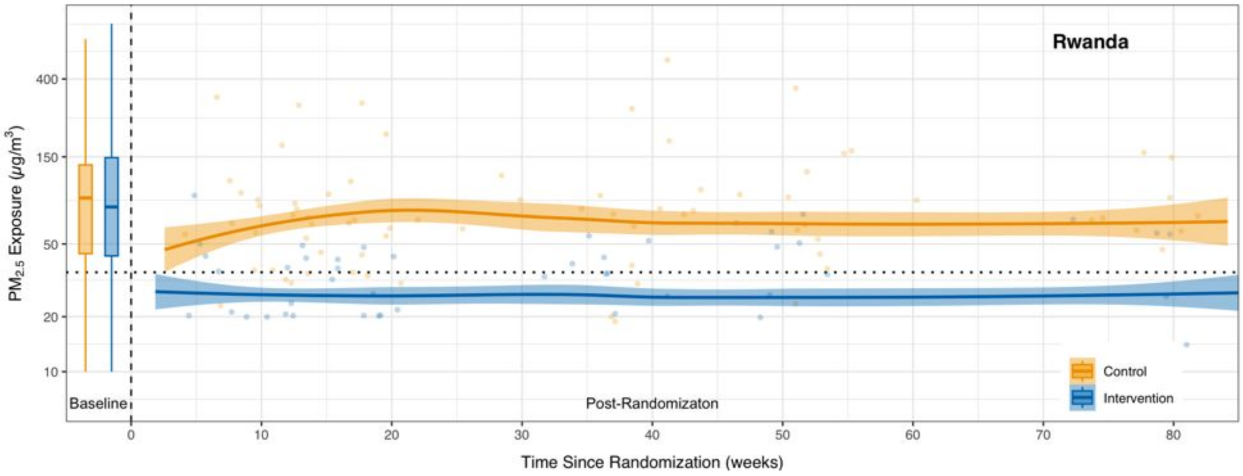

Figure S7. Trends in personal $PM_{2.5}$ exposure among the HAPIN non-pregnant adult women participants in each IRC.

The x-axis is the time since randomization (in weeks); time before 0 indicates the baseline period. Distribution of baseline exposures are presented as box plots. The lower and upper hinges correspond to the first and third quartiles (the 25th and 75th percentiles). The upper and lower whiskers extend 1:5 × IQR above and below the upper and lower hinges. Data beyond the whiskers are outliers. Solid lines are a locally weighted smoothing (LOWESS) function. Shaded areas are standard errors. Orange (lighter) points are individual data points from control households; Blue (darker) points are from intervention households. Note: IQR, interquartile range.

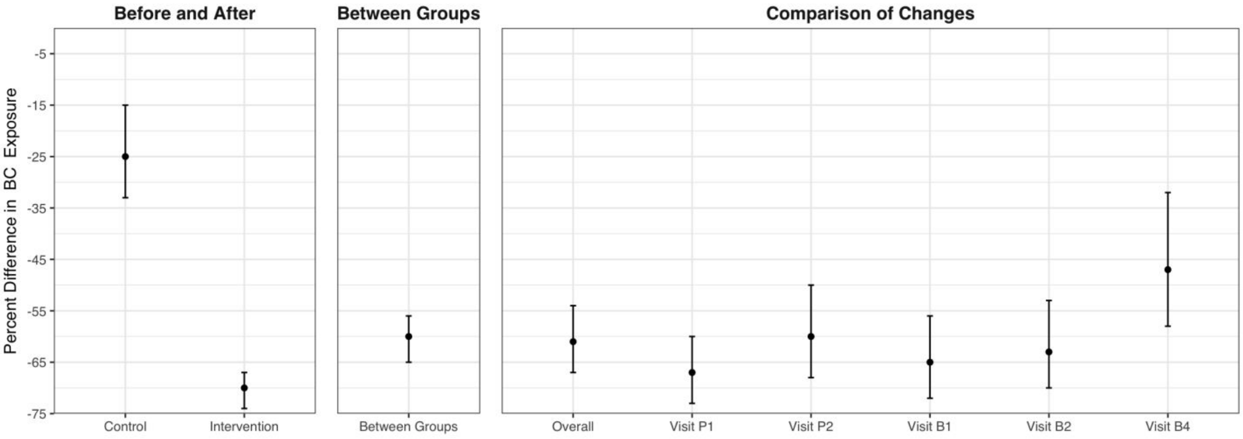

Figure S8. Estimated effects of the HAPIN LPG stove and fuel intervention on BC exposure.
All linear mixed-effects models used log-transformed BC as the dependent variable. Whiskers indicate the 95% confidence intervals.

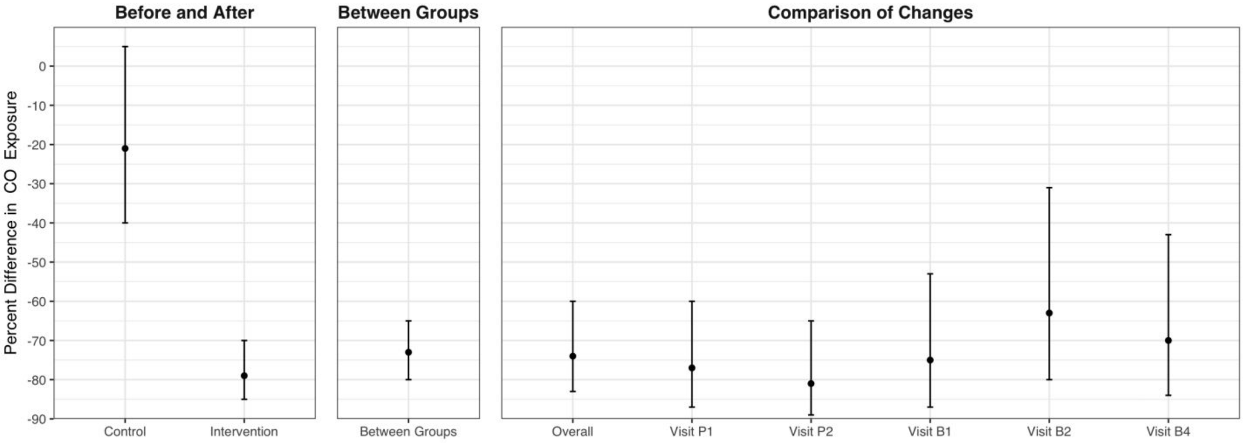

Figure S9. Estimated effects of the HAPIN LPG stove and fuel intervention on CO exposure.
All linear mixed-effects models used log-transformed CO as the dependent variable. Whiskers indicate the 95% confidence intervals.

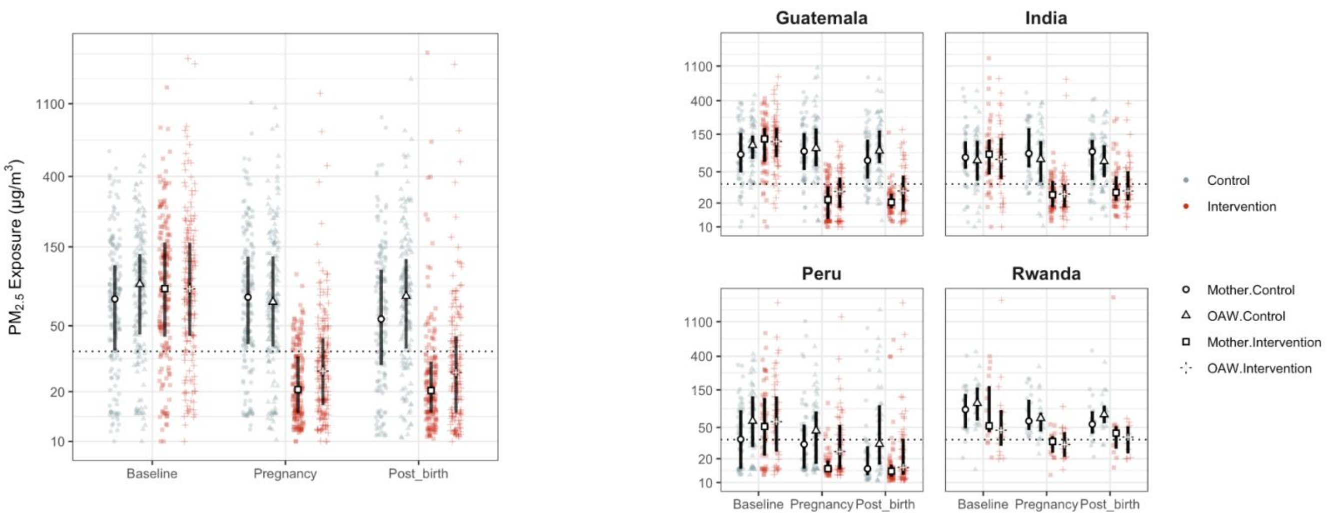

Figure S10. Trial-wide and IRC-specific comparison of personal exposures to PM2.5 between pregnant and non-pregnant adult women in the same households by arm and study period

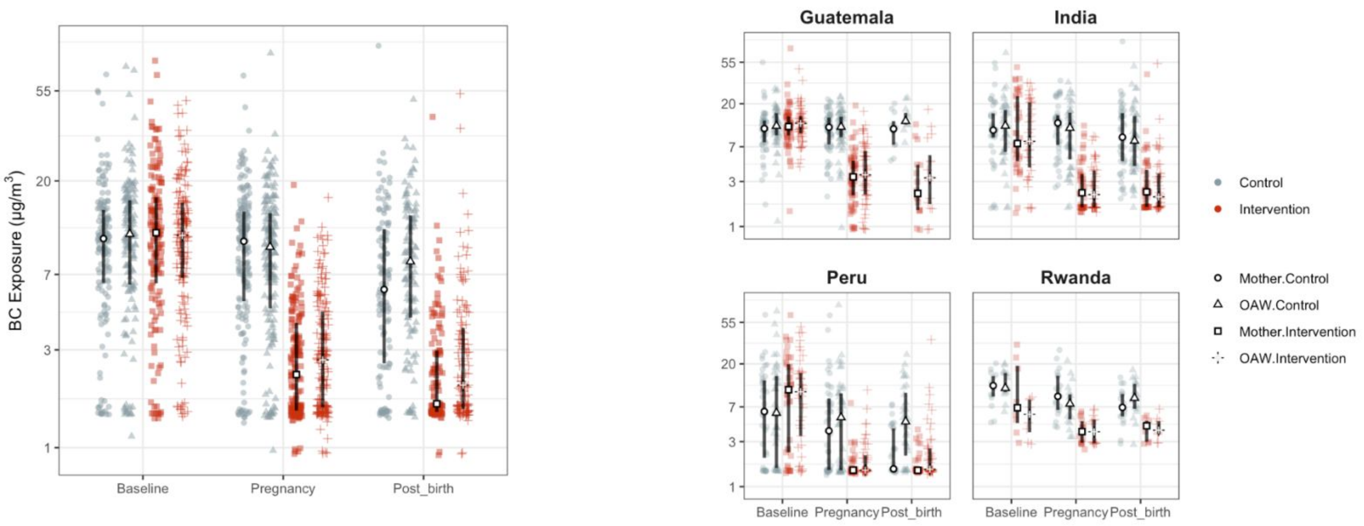

Figure S11. Trial-wide and IRC-specific comparison of personal exposures to BC between pregnant and non-pregnant adult women in the same households by arm and study period

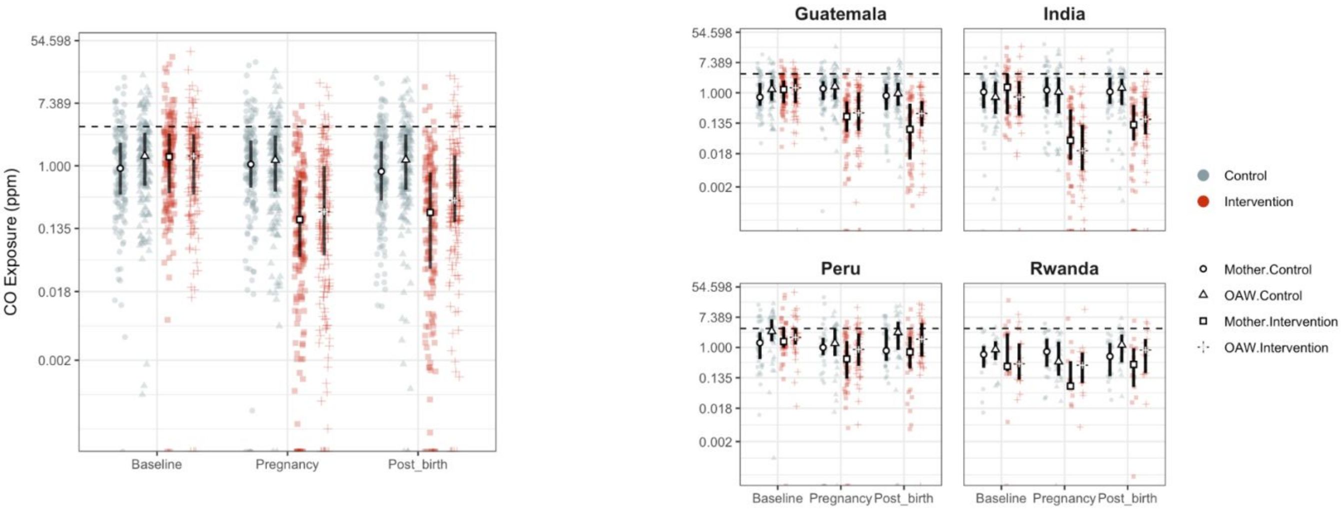

Figure S12. Trial-wide and IRC-specific comparison of personal exposures to CO between pregnant and non-pregnant adult women in the same households by arm and study period

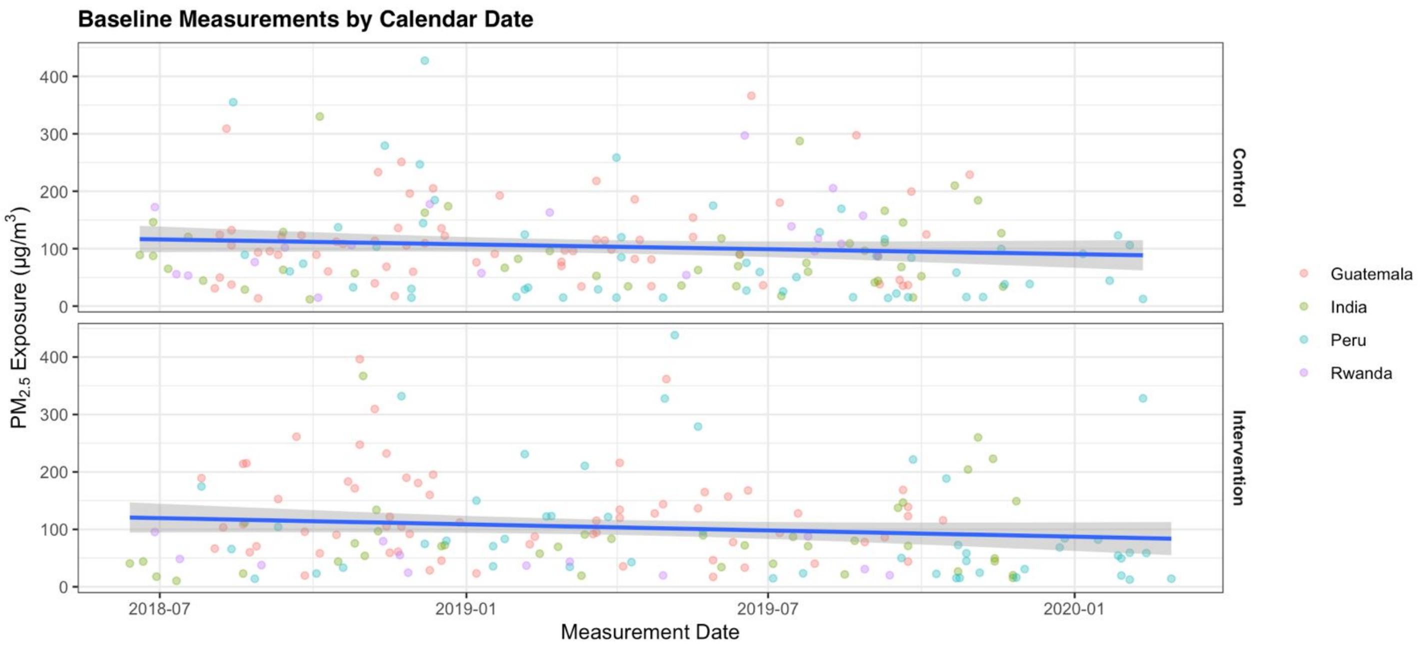

Figure S13. Baseline Measurements by Calendar Date.
The x-axis is the date of the exposure measurement; the Y axis is the measured PM2.5 level, and the line is a linear model evaluating time trends. Shaded areas are uncertainty bounds.

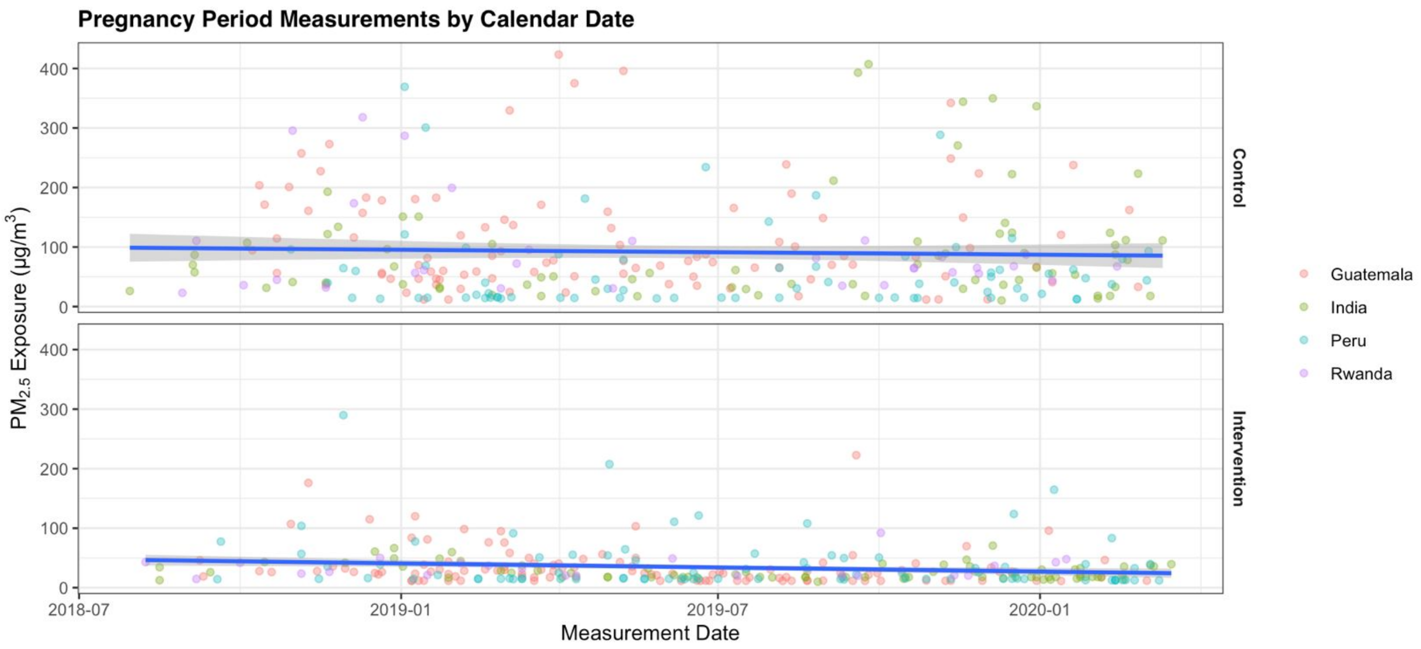

Figure S14. Pregnancy Period Measurements by Calendar Date.
The x-axis is the date of the exposure measurement; the Y axis is the measured PM2.5 level, and the line is a linear model evaluating time trends. Shaded areas are uncertainty bounds.

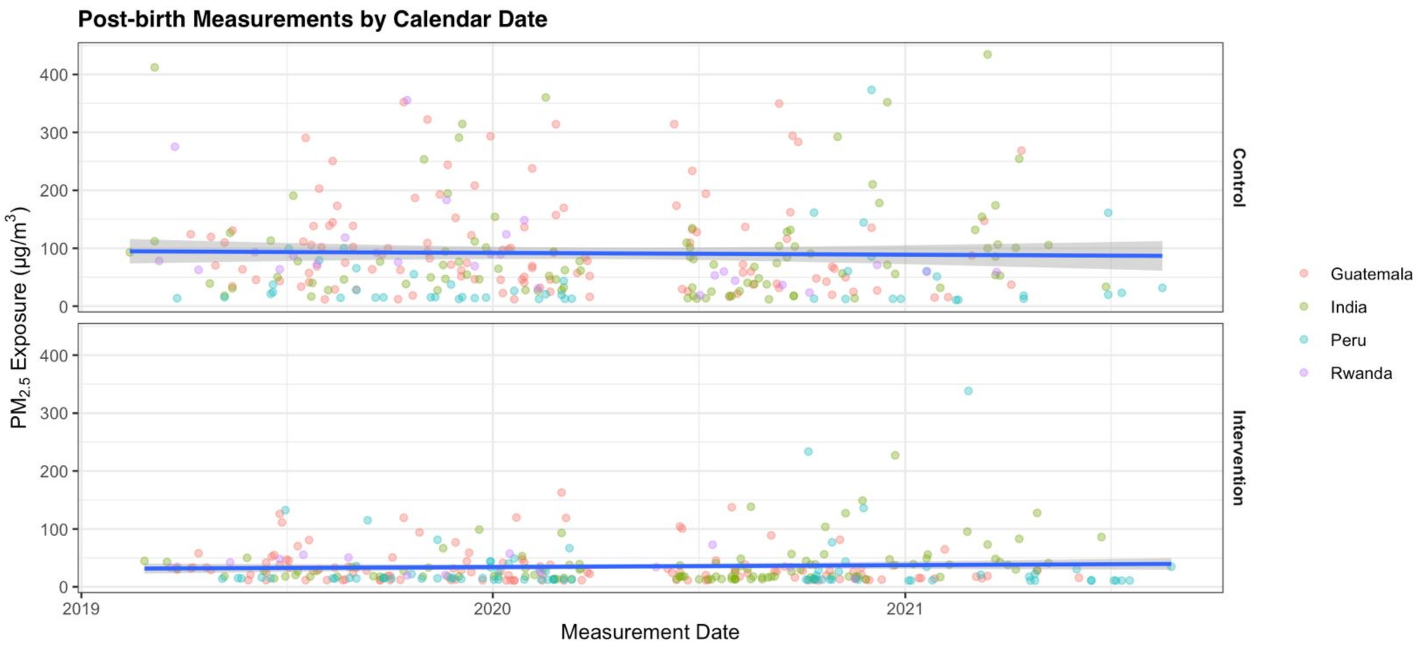

Figure S15. Post-birth Measurements by Calendar Date.
The x-axis is the date of the exposure measurement; the Y axis is the measured PM2.5 level, and the line is a linear model evaluating time trends. Shaded areas are uncertainty bounds.
